# Community Health Workers Linking Clinics and Schools and Asthma Control: A Randomized Clinical Trial

**DOI:** 10.1001/jamapediatrics.2024.3967

**Published:** 2024-10-21

**Authors:** Tyra Bryant-Stephens, Chen C. Kenyon, Colleen Tingey, Andrea Apter, Julie Pappas, Natalie Minto, Yvonne S. Stewart, Justine Shults

**Affiliations:** 1 Division of General Pediatrics, Community Asthma Prevention Program, Children’s Hospital of Philadelphia, Philadelphia; 2 PolicyLab and Division of General Pediatrics, Children’s Hospital of Philadelphia, Department of Pediatrics, University of Pennsylvania School of Medicine, Philadelphia; 3 Children’s Hospital of Philadelphia, Philadelphia, Pennsylvania; 4 Division of Pulmonary Allergy Critical Care, Perelman School of Medicine, University of Pennsylvania, Philadelphia; 5 Westat Inc, Rockville, Maryland; 6 West Philadelphia Asthma Control Collaborative (WEPACC), Philadelphia, Pennsylvania; 7 National Multiple Sclerosis Society, Philadelphia, Pennsylvania; 8 Restoring Health Ministries, Philadelphia, Pennsylvania; 9 Department of Pediatrics, Children’s Hospital of Philadelphia, Department of Biostatistics, Epidemiology, and Informatics, Perelman School of Medicine at the University of Pennsylvania, Philadelphia, Pennsylvania

## Abstract

**Importance:**

Childhood asthma is characterized by pervasive disparities, including 3-fold higher hospitalization rates and 7-fold higher death rates for Black children compared with White children. To address asthma disparities, one must intervene in all lived environments.

**Objective:**

To determine if a community health worker (CHW) intervention to connect the primary care, home, and school for low-income minoritized school-aged children with asthma and their caregivers improves asthma control.

**Design, Setting, and Participants:**

This study was a hybrid effectiveness/implementation trial using a 2 × 2 factorial, cluster randomized clinical trial design of 36 schools crossed with participant-level randomization into a clinic-based CHW intervention. The study was conducted from May 2018 to June 2022. The intervention took place in primary care offices, homes, and 36 West Philadelphia, Pennsylvania, public and charter schools. Children aged 5 to 13 years with uncontrolled asthma were recruited from local primary care practices.

**Interventions:**

Asthma management, trigger remediation, and care coordination occurred in school, home, and primary care settings. Children were followed up for 12 months. The Yes We Can Children’s Asthma Program, Open Airways For Schools Plus, and school-based asthma therapy were implemented.

**Main Outcomes and Measures:**

Improvement in asthma control, as measured by the Asthma Control Questionnaire, comparing the mean difference between groups 1 year after randomization with their baseline (difference in differences). Both primary care and school interventions were dramatically disrupted by the COVID-19 pandemic; therefore, stratified analyses were performed to assess per-protocol intervention efficacy before the pandemic disruptions.

**Results:**

A total of 1875 participants were approached, 1248 were excluded, and 1 was withdrawn. The 626 analyzed study participants (mean [SD] age, 8.7 [2.4] years; 363 male [58%]) self-identified as Black race (96%) and non-Hispanic ethnicity (98%). Although all groups had statistically significant improvements in asthma control from baseline to 12 months (P− group: −0.46; 95% CI, −0.58 to −0.33; P+ group: −0.57; 95% CI, −0.74 to −0.44; S− group: −0.47; 95% CI, −0.58 to −0.35; S+ group: −0.59; 95% CI, −0.74 to −0.44), none of the difference-in-differences estimates from the primary prespecified models showed a clinically meaningful improvement in asthma control. Analysis from the prepandemic interval, however, demonstrated that children in the combined clinic-school intervention had a statistically significant improvement in asthma control scores compared with control (−0.79; 95% CI, −1.40 to −0.18).

**Conclusions and Relevance:**

This randomized clinical trial provides preliminary evidence that connecting all lived environments for care of children can be accomplished through linkages with CHWs.

**Trial Registration:**

ClinicalTrials.gov Identifier: NCT03514485

## Key Points

**Question:**

Does the participation of community health workers in an intervention to connect home, school, the health care system, and community for underserved school-aged children with asthma and their caregivers improve asthma control?

**Findings:**

In this randomized clinical trial with 626 children, all groups had statistically significant improvements in asthma control from baseline to 12 months with no differences between groups.

**Meaning:**

The findings of this study support that connecting all lived environments for care of children with asthma is feasible through linkages by community health workers.

## Teaser

This randomized clinical trial investigates if the participation of community health workers in an intervention to connect home, school, the health care system, and community for underserved school-aged children with asthma and their caregivers improves asthma control.

## Introduction

Childhood asthma in the US is characterized by persistent disparities in asthma morbidity and mortality. For instance, Black children have a hospitalization rate 3 times higher, and a death rate 7 times higher, than their White counterparts.^1,2^ These disparities have been attributed to a multitude of socioecological factors including less frequent receipt of guideline-concordant, coordinated care,^3,4,5,6,7^ lower controller medication use,^3,4,5,6,7^ suboptimal housing^8^ and school environmental conditions,^9,10,11,12^ and neighborhood exposures.^13,14^ School-aged children with asthma encounter many daily environments—home, school, and community—yet often, their asthma management is fragmented, and families must navigate asthma care in each area independently. This results in more school days missed, reduced adherence to controller medications, and poor academic performance.^15^ Previous interventions for this population have focused on individual environments or 2 environments, neglecting the important connections between home, school, health care system, and community environments. Creating connections and coordinating asthma care management across all of these settings is a promising approach to improve asthma outcomes.

Ample evidence supports the impact of community health worker (CHW) interventions in improving pediatric asthma care and outcomes.^16,17^ In 2016, we conducted a mixed-methods analysis to determine the best approach to coordinating care in West Philadelphia, Pennsylvania, where 25% of children are diagnosed with asthma, to improve asthma control.^18^ Parents reported frustration with fragmentation of care. School nurses reported lack of connection to parents and primary care physicians. Primary care practitioners reported a desire to have 2-way communication with school nurses. The findings, previously described,^19^ led to the West Philadelphia Asthma Care Implementation study, in which CHWs implement evidence-based interventions to close gaps in asthma care by connecting home, health care, school, and community.^19^ Our intervention thus focused on connecting home, school, clinic and community. We report the results of this randomized clinical trial that used a factorial design to compare usual care with CHW interventions conducted in primary care alone, school alone, and combined primary care-school settings. Our a priori hypothesis was that children receiving CHW-delivered evidence-based interventions coordinated across primary care and school settings would achieve better asthma control than those receiving usual care or interventions in either setting alone for children in low-income, poorly resourced communities.

## Methods

### Study Design and Setting

We used a 2 × 2 factorial design to evaluate the effectiveness of evidence-based interventions delivered by CHWs in primary care clinics, schools, and home settings over 12 months. However, unlike in a classical 2 × 2 factorial design, school-based asthma therapy (SBAT) was only delivered to the combination intervention group. Individual participants were enrolled during clinic visits and randomized to the clinic-based intervention, stratified by school and clinic site. School-level randomization was stratified by 5 predefined groups to ensure balance in baseline school characteristics. Details of this study’s design, setting, randomization, and consent procedures have been previously described (Supplement 1).^19^ The institutional review board at the Children’s Hospital of Philadelphia (CHOP) approved and reviewed the research protocol. This study followed the Consolidated Standards of Reporting Trials (CONSORT) reporting guidelines.

### Study Timeline and the COVID-19 Pandemic

The study was conducted from May 2018 to June 2022. The COVID-19 SARS-CoV-2 viral pandemic dramatically altered all environments in which the study was being conducted, and social distancing measures led to dramatic reductions in asthma morbidity locally.^20^ The greatest impact of the pandemic on the intervention was on the study team’s ability to deliver the school component as designed because the schools shut down in early spring 2020 and, when they reopened in the fall of 2020, the academic year was a combination of completely virtual and hybrid format. When children returned in person, school staff had many competing priorities, including high burdens of absenteeism, mental health crises, and recouping the educational losses. We attempted to conduct aspects of the school intervention virtually; however, each school had different challenges, resulting in a weakened and inconsistent delivery of the school interventions. A description of the COVID-19 pandemic–associated study adaptations is included in Supplement 1.

### Eligibility and Recruitment

Eligibility criteria included youth aged 5 to 13 years, having 1 systemic steroid for asthma in the past year or 1 emergency department visit, and residence in West Philadelphia. Participants were identified using CHOP’s electronic health record (EHR) and recruited from 4 participating primary care sites. Race and ethnicity were assessed because Black American and Hispanic or Latino children have higher asthma morbidity.^19^ Participant- or caregiver-identified races and ethnicities included Black or African American and Hispanic or Latino. Children were randomized to receive a primary care CHW (P+) or usual care (P−). Participating schools were randomized to receive the CHW-led school intervention (S+) or usual care in schools (S−). Children who did not attend a participating school were not eligible for school-level interventions. During the COVID-19 pandemic, we modified recruitment to be virtual rather than in person. Recruitment began in May 2018 and finished in June 2021 as per protocol.

### Primary Care Intervention

Children randomized to the child-level intervention were assigned a primary care CHW and enrolled in the Yes We Can Children’s Asthma Program^21^ and Community Asthma Prevention Program (CAPP) intervention.^22^ The primary care CHW, who was integrated into the primary care practice, conducted in-office asthma education about asthma medications, devices, and the asthma care plan; accompanied the caregiver to the child’s primary care appointments; and attended 4 home visits, each providing specific asthma education.

### School Intervention

Thirty-six participating West Philadelphia elementary schools were randomized to receive either the school intervention, facilitated by a school CHW, or usual school care. The intervention was designed to contain 4 components of the school-based asthma management program guidelines and included asthma management training for children using Open Airways for Schools Plus,^15^ school professional education, classroom asthma trigger remediation, and asthma care plans. SBAT^23^ was provided solely to the combination intervention group. Controller medications were delivered to the school by an independent pharmacy, and medication use was recorded by the school nurse or school CHW.

### Data Collection and Outcome Measures

#### Primary Outcome

The primary outcome of asthma control was measured using Juniper’s Asthma Control Questionnaire (ACQ), a validated instrument for children and adults.^24^ This is a 6-point scale with lower scores indicating better control and a threshold above 1.25 considered poor control for children.^25^ Based on existing literature, the minimal clinically important difference (MCID) is 0.5.^26^

#### Secondary Outcomes

Symptom-free days were collected by caregiver report recall of the last 2 weeks, consistent with previous studies.^27,28^ School absences were measured by school and parental report. Asthma-related emergency visits, hospitalizations, and oral corticosteroid prescriptions were obtained through CHOP’s EHR. Caregiver quality of life data were collected using the Juniper’s Pediatric Asthma Caregiver’s Quality of Life Questionnaire, which consists of 2 domains (activity limitations and emotional functioning), with higher scores indicating better quality of life.^29^

Data were collected for all individually randomized participants at baseline, 3, 6, 9, and 12 months by a member of the research team in person, virtually, or by telephone. Follow-up data collection was also attempted at 24 months. Participants were compensated $25 for each visit with data collection (baseline, 3, 6, and 9 months) and $45 for the 12-month end of study visit (time point for primary comparisons).

### Adverse Events

Adverse events were collected via EHR reporting. Severity and relatedness to the study procedures were determined by qualified study team members (T.B.S., C.K.) who were blinded to treatment group assignments. All data were reported and reviewed by an independent data safety and monitoring board, with no safety concerns identified.

### Statistical Analysis

Analyses were performed using Stata, version 18 statistical software (StataCorp), with 2-sided tests of hypotheses and a *P* value <.05 as the criterion for statistical significance. Sample size calculation and power are previously described.^19^ Some participants withdrew, and others were lost to follow-up for reasons including withdrawal of consent, discontinued interest in participation, and research burden. The statistical analysis plan (SAP) for the study is provided in Supplement 2. Unless specified otherwise, all analyses were performed according to the SAP.

#### Primary Outcome

Linear mixed-effects models were used to make comparisons of all combinations of primary care and school-based interventions with respect to asthma control. The models included random intercepts for the participant and school, to account for correlation within participants over time and within students in each school. Time was defined as the number of days since randomization (day of follow-up) and was modeled with splines, with knots at time 3, 6, and 9 months, the intended dates of measurements. Models that included time, treatment and time by treatment interactions were used to express the expected value for asthma control for a particular treatment group and time point as a linear combination of the model parameters. Contrasts across time and levels of the intervention were obtained by subtraction. Fitted values (and within and between treatment group differences, with 95% CI) were presented for baseline and 12 months.

#### Pandemic Modifications

We conducted several data safety monitoring board–approved additional analyses to assess the impact of COVID-19 pandemic restrictions on study outcomes. The restrictions start date was set at March 19, 2020, based on local mandates. First, the longitudinal models for the main analyses (the mixed-effects models with time modeled using splines) were modified to include an indicator variable for pre– (vs post–) COVID-19 restriction phase, with time by post–COVID-19 interaction and time by post–COVID-19 by intervention interaction terms. In these models, the pre–COVID-19 phase was included as a time varying-covariate within individuals. We used likelihood ratio tests to compare the models with the postpandemic phase by treatment group interactions terms to the models without the interaction terms. Fitted values are displayed for a linear generalized estimating equation model, with an indicator variable for pre– (vs post–) COVID-19 period.

Second, we performed a post hoc stratified analysis (not described in the SAP) in accordance with the following recommendation: “In some cases, the primary analysis would exclude intermittent intervals of calendar time that meet prespecified site specific criteria for severe disruption from the COVID-19 pandemic (eg, substantively reduced ability to deliver blinded study drug or to retain participants).”^30,31^ We therefore excluded the postshutdown time period, during which severe disruption from the COVID-19 pandemic (closure of all schools in Philadelphia) occurred (stratified by study completion before the COVID-19 pandemic phase).

#### Secondary Outcomes

Secondary outcomes were measured at baseline and 12 months. Fitted values were obtained using generalized linear mixed-effects models for each secondary outcome with distributions and link functions as described in the legend. Random effects for school and participant were initially included in the models, to account for intraschool and intraparticipant correlations. If the initial models failed to converge, simpler models were fitted. The Akaike and bayesian information criteria were used to compare fit of models, eg, to compare the Poisson with negative binomial distribution for count outcomes.

## Results

**Figure 1.**
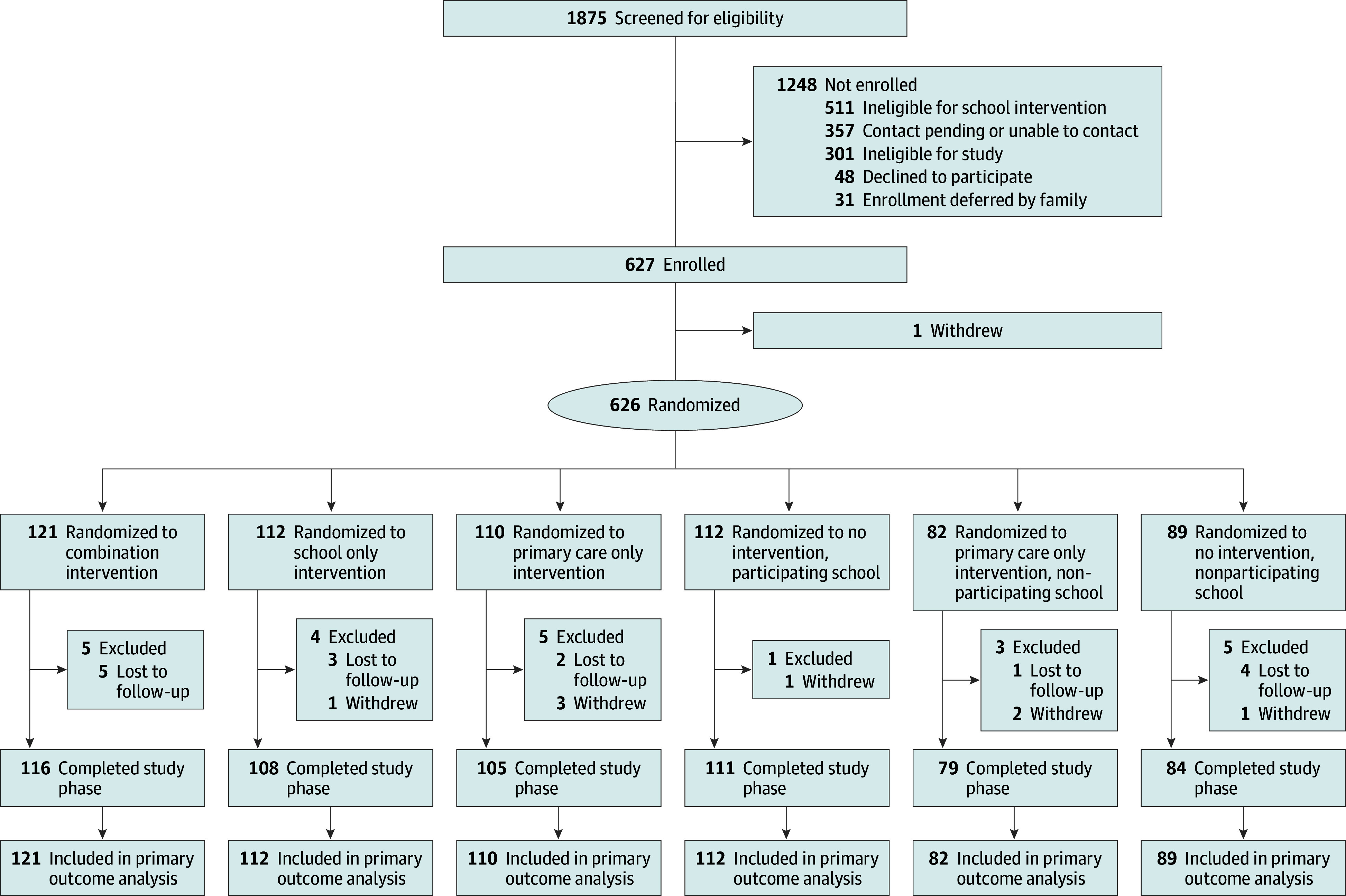
Participant Flow in the West Philadelphia Controls Asthma Randomized Clinical Trial

Of the 1875 participants screened for eligibility, 1248 were excluded, and 1 was withdrawn. A total of 626 participants (mean [SD] age, 8.7 [2.4] years; 263 female [42%]; 363 male [58%]) were randomized (Figure 1). Study participants were predominantly Medicaid insured (87%), 601 (96%) self-identified as Black race, 14 (2%) self-identified as having Hispanic or Latino ethnicity, and 612 (98%) identified as having non-Hispanic ethnicity. The mean (SD) baseline asthma control questionnaire score was 1.4 (1.2), with 47% of participants (294 of 626) having a score greater than or equal to 1.3, indicating poor control. In the year before enrollment, 52% of participants (326 of 626) had an asthma-related emergency department visit, 34% (213 of 626) had an asthma-related hospitalization, and all participants but one reported at least 1 asthma-related systemic steroid prescription. Baseline participant characteristics, including demographics and baseline asthma morbidity, were similar between intervention groups (Table 1).

**Table 1.** Baseline Characteristics by Treatment Group

Demographic variables	Groups, No. (%)					
	A: P+S+	B: P−S+	C: P+S−	D: P−S−	E: P+S^0^	F: P−S^0^
No.	121	112	110	112	82	89
Age, y						
Mean (SD)	9.3 (2.4)	8.8 (2.3)	8.7 (2.6)	8.4 (2.4)	8.4 (2.4)	8.5 (2.4)
Median (IQR) [range]	8.8 (7.2-11.3) [5.3-13.8]	8.7 (6.7-10.5) [5.2-13.9]	8.0 (6.4-10.7) [5.1-13.7]	7.7 (6.3-10.7) [5.1-13.6]	8.1 (6.3-10.3) [5.0-13.9]	8.1 (6.5-10.1) [5.1-13.7]
Sex						
Female	61 (50.4)	43 (38.4)	45 (40.9)	40 (35.7)	34 (41.5)	40 (44.9)
Male	60 (49.6)	69 (61.6)	65 (59.1)	72 (64.3)	48 (58.5)	49 (55.1)
Insurance						
Medicaid or CHIP	111 (91.7)	101 (90.2)	101 (91.8)	101 (90.2)	75 (91.5)	72 (80.9)
Private	4 (3.3)	6 (5.4)	3 (2.7)	6 (5.4)	6 (7.3)	15 (16.9)
Other (includes other and unknown and refused and none)	6 (5.0)	5 (4.5)	4 (3.6)	4 (3.6)	0	2 (2.2)
Race						
Black or African American	119 (98.3)	103 (92.0)	106 (96.4)	106 (94.6)	79 (96.3)	88 (98.9)
Ethnicity						
Hispanic or Latino	3 (2.5)	2 (1.8)	5 (4.5)	3 (2.7)	1 (1.2)	0
BMI						
Below 85th percentile	57 (47.1)	64 (57.1)	46 (41.8)	59 (52.7)	34 (41.5)	45 (50.6)
85th to 94th percentile	12 (9.9)	15 (13.4)	15 (13.6)	15 (13.4)	18 (22.0)	13 (14.6)
At or above 95th percentile	50 (41.3)	31 (27.7)	44 (40.0)	37 (33.0)	29 (35.4)	30 (33.7)
Missing	2 (1.7)	2 (1.8)	5 (4.5)	1 (0.9)	1 (1.2)	1 (1.1)
Asthma Control Score						
Mean (SD)	1.3 (1.3)	1.4 (1.3)	1.5 (1.2)	1.3 (1.2)	1.3 (1.2)	1.4 (1.3)
Median (IQR) [range]	1.0 (0.3-2.1) [0.0-5.3]	1.2 (0.3-2.2) [0.0-6.0]	1.3 (0.3-2.2) [0.0-5.2]	1.2 (0.1-2.2) [0.0-4.3]	1.0 (0.1-2.2) [0.0-4.2]	1.2 (0.2-2.3) [0.0-5.0]
Range No.	120	112	109	112	80.0	89.0
Asthma Control Score groups						
0 to <1.25	68 (56.2)	58 (51.8)	51 (46.4)	59 (52.7)	43(52.5)	49 (55.1)
≥1.25	52 (43.0)	54 (48.2)	58 (52.7)	53 (47.3)	37 (45.1)	40 (44.9)
Missing	1 (0.8)	0	1 (0.9)	0	2 (2.4)	0
Hospitalized overnight for asthma						
Ever	78 (64.5)	73 (65.2)	79 (71.8)	71 (63.4)	46 (56.1)	61 (68.5)
Past 12 mo^a^	36 (29.8)	40 (35.7)	51 (46.4)	33 (29.5)	23 (28.0)	31 (34.8)
In the ICU for asthma						
Ever	17 (14.0)	12 (10.7)	17 (15.5)	9 (8.0)	9 (11.0)	11 (12.4)
Past 12 mo	0	1 (0.9)	5 (4.5)	1 (0.9)	1 (1.2)	0
Emergency department for asthma						
Ever	109 (90.1)	94 (83.9)	90 (81.8)	92 (82.1)	74 (90.2)	78 (87.6)
Past 12 mo^a^	66 (54.5)	60 (53.6)	64 (58.2)	46 (41.1)	43 (52.4)	49 (55.1)
Taking a prednisone burst for asthma						
Ever	121 (100)	112 (100)	110 (100)	112 (100)	82 (100)	89 (100)
Past 12 mo	120 (99.2)	112 (100)	110 (100)	112 (100)	82 (100)	89 (100)
Quality of life score overall						
Mean (SD)	5.5 (1.2)	5.6 (1.2)	5.2 (1.4)	5.4 (1.3)	5.7 (1.1)	5.5 (1.4)
Median (IQR) [range]	5.8 (4.9-6.6) [1.0-7.0]	5.8 (5.0-6.5) [1.0-7.0]	5.5 (4.3-6.4) [1.2-7.0]	5.6 (4.7-6.5) [2.2-7.0]	5.9 (4.8-6.7) [2.6-7.0]	5.8 (4.5-6.5) [1.7-7.0]
Range No.	120	112	108	111	80.0	88.0
Maternal education						
At least some college/trade school	55 (45.5)	41 (36.6)	44 (40.0)	46 (41.1)	46 (56.1)	53 (59.6)
At most high school graduate or equivalent	66 (54.5)	71 (63.4)	64 (58.2)	65 (58.0)	35 (42.7)	36 (40.4)
Missing	0	0	2 (1.8)	1 (0.9)	1 (1.2)	0

Abbreviations: BMI, body mass index; CHIP, Children’s Health Insurance Program; ICU, intensive care unit; P+, primary care community health worker; P−, usual care; S+, community health worker–led school intervention; S−, usual care in schools; S^0^, S− the group without school intervention.

^a^ Data reported from the electronic health records.

### Primary Outcome

**Figure 2.**
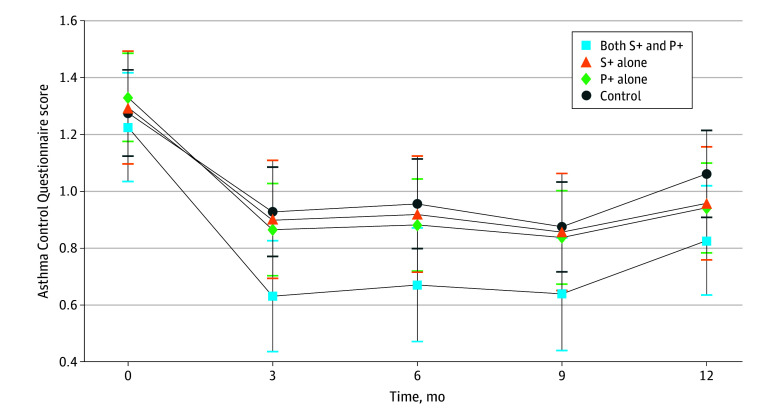
Graph of Fitted Values of Asthma Control vs Planned Month of Visit At baseline (month 0) the number of participants in each treatment group = 120 for community health worker (CHW)–led school intervention (S+) and primary care CHW (P+), 112 for S+ alone, 189 for P+ alone, and 201 for control. At month 3, the number of participants in each treatment group = 111 for S+P+, 97 for S+ alone, 166 for P+ alone, and 179 for control. At month 9, the number of participants in each treatment group = 104 for S+P+, 99 for S+ alone, 160 for P+ alone, and 178 for control. At month 12, the number of participants in each treatment group = 116 for S+P+, 108 for S+ alone, 182 for P+ alone, and 195 for control. This analysis excluded 1 measurement taken after 800 days of follow-up.

Figure 2 demonstrates the changes in ACQ score by study group over the 12-month active intervention period. All groups experienced the greatest absolute improvement in asthma control score in the first 3 months, with the combined intervention demonstrating the greatest absolute reduction. All groups had statistically significant improvements in asthma control from baseline to month 12 (P− group: −0.46; 95% CI, −0.58 to −0.33; P+ group: −0.57; 95% CI, −0.74 to −0.44; S− group: −0.47; 95% CI, −0.58 to −0.35; S+ group: −0.59; 95% CI, −0.74 to −0.44), but only the active intervention groups exceeded the MCID of 0.5 when compared with their baseline average (Table 2). Results for participants with all visits after the shutdown and for those with some visits before and after the shutdown are provided in eFigures 1 to 15 in Supplement 3.

**Table 2.** Fitted Values From a Mixed-Effects Model for Asthma Control at 12 Months^a^

Variable	Baseline (95% CI)	Month 12 (95% CI)	Difference [month 12 − baseline] (95% CI)
**No adjustment for COVID-19 pandemic phase**			
First comparison group: P− (n = 313)	1.38 (1.26 to 1.5)	0.92 (0.81 to 1.04)	−0.46 (−0.58 to −0.33)
Second comparison group: P+ (n = 309)	1.36 (1.24 to 1.48)	0.80 (0.69 to 0.90)	−0.57 (−0.74 to −0.44)
Difference (second comparison group − first comparison group)	−0.02 (−0.18 to 0.15)	−0.13 (−0.28 to 0.02)	−0.11 (−0.29 to 0.07)
First comparison group: S− (n = 390)	1.37 (1.27 to 1.48)	0.91 (0.81 to 1.00)	−0.47 (−0.58 to −0.35)
Second comparison group: S+ (n = 232)	1.38 (1.25 to 1.52)	0.79 (0.67 to 0.92)	−0.59 (−0.74 to −0.44)
Difference (second comparison group − first comparison group)	0.01 (−0.16 to 0.18)	−0.11 (−0.27 to 0.04)	−0.13 (−0.31 to 0.06)
Control (n = 201)	1.37 (1.22 to 1.51)	0.97 (0.84 to 1.03)	−0.39 (−0.55 to −0.23)
S+ alone (n = 112)	1.42 (1.23 to 1.62)	0.85 (0.67 to 1.03)	−0.57 (−0.78 to −0.36)
P+ alone (n = 189)	1.38 (1.23 to 1.53)	0.85 (0.72 to 0.99)	−0.53 (−0.69 to −0.37)
S+P+ both (n = 120)	1.35 (1.16 to 1.54)	0.74 (0.56 to 0.92)	−0.61 (−0.82 to −0.40)
S+P+ both vs S+ alone (difference)	−0.07 (−0.34 to 0.2)	−0.11 (−0.37 to 0.25)	−0.04 (−0.34 to 0.25)
S+P+ both vs P+ alone (difference)	−0.03 (−0.27 to 0.21)	−0.11 (−0.34 to 0.11)	−0.08 (−0.34 to 0.18)
S+ alone vs control (difference)	0.06 (−0.19 to 0.30)	−0.12 (−0.35 to 0.10)	−0.18 (−0.44 to 0.09)
P+ alone vs control (difference)	0.02 (−0.19 to 0.22)	−0.12 (−0.31 to 0.07)	−0.14 (−0.36 to 0.09)
S+P+ both vs control (difference)	−0.02 (−0.25 to 0.22)	−0.23 (−0.46 to −0.01)	−0.22 (−0.48 to 0.04)
**Stratified by study completion before COVID-19 pandemic phase**			
First comparison group: P− (n = 54)	1.72 (1.41 to 2.04)	1.27 (0.99 to 1.55)	−0.46 (−0.78 to −0.14)
Second comparison group: P+ (n = 60)	1.78 (1.48 to 2.08)	0.96 (0.69 to 1.22)	−0.82 (−1.13 to −0.51)
Difference (second comparison group − first comparison group)	0.05 (−0.38 to 0.48)	−0.31 (−0.69 to 0.07)	−0.36 (−0.81 to 0.08)
First comparison group: S− (n = 77)	1.64 (1.37 to 1.90)	1.15 (0.92 to 1.39)	−0.48 (−0.75 to −0.22)
Second comparison group: S+ (n = 37)	1.99 (1.61 to 2.37)	0.99 (0.64 to 1.33)	−1.01 (−1.40 to −0.61)
Difference (second comparison group − first comparison group)	0.36 (−0.11 to 0.82)	−0.17 (−0.58 to 0.25)	−0.53 (−1.00 to −0.05)^b^
Control (n = 40)	1.61 (1.25 to 1.97)	1.26 (0.94 to 1.78)	−0.35 (−0.72 to 0.01)
S+ alone (n = 14)	2.04 (1.43 to 2.65)	1.19 (0.61 to 1.78)	−0.85 (−1.51 to −0.20)
P+ alone (n = 37)	1.66 (1.29 to 2.04)	1.02 (0.69 to 1.36)	−0.64 (−1.02 to −0.26)
S+P+ both (n = 23)	1.97 (1.49 to 2.45)	0.83 (0.40 to 1.26)	−1.14 (−1.63 to −0.65)
S+P+ both vs S+ alone (difference)	−0.08 (−0.85 to 0.70)	−0.36 (−1.08 to .53)	−0.29 (−1.10 to 0.53)
S+P+ both vs P+ alone (difference)	0.30 (−0.30 to 0.91)	−0.20 (−0.74 to 0.35)	−0.50 (−1.12 to 0.12)
S+ alone vs control (difference)	0.43 (-0.28 to 1.14)	−0.07 (−0.73 to 0.60)	−0.50 (−1.25 to 0.25)
P+ alone vs control (difference)	0.05 (−0.47 to 0.57)	−0.23 (−0.70 to 0.23)	−0.29 (−0.82 to 0.24)
S+P+ both vs control (difference)	0.36 (−0.24 to 0.96)	−0.43 (−0.96 to 0.10)	−0.79 (−1.40 to −0.18)^b^

Abbreviations: P+, primary care community health worker; P−, usual care; S+, community health worker–led school intervention; S−, usual care in schools.

^a^ Results are provided for all participants (no adjustment for COVID-19 pandemic phase) and for participants who had their 12-month visit before shutdown (study completion before COVID-19 pandemic). Asthma control (primary outcome): mixed-effects model with random intercepts for school and child and a Markov correlation structure for the residuals. The models included time, indicator variables for each intervention, and time by intervention interaction terms. Time was modeled with splines, with knots at day of follow-up (current date minus date of randomization) 91, 182, and 273 (the planned days of measurement). More information is available in eFigure 1 in Supplement 2.

^b^ *P* < .05.

Comparing the change from baseline to month 12 between comparison groups, participants in the active intervention groups experienced greater improvements in asthma control, but none of these difference in differences met thresholds for statistical significance or clinical meaningfulness (Table 2). The largest absolute difference in differences were between participants receiving the combined clinic- and school-level intervention group compared with the usual care group: −0.22 (95% CI, −0.48 to 0.04). Including a covariate to account for interventions delivered during the COVID-19 pandemic produced similar results to the primary linear mixed-effects model.

Primary outcome measurement occurred for 114 children before the local onset of the COVID-19 pandemic and the requisite modifications of study interventions. In stratified analysis of children who had their 12-month visit before the shutdown for the pandemic, all groups showed improved asthma control scores when comparing their 12-month asthma control to baseline, with active intervention groups exceeding the MCID (P− group: −0.46; 95% CI, −0.78 to −0.14; P+ group: −0.82; 95% CI, −1.13 to −0.51; S− group: −0.48; 95% CI, −0.75 to −0.22; S+ group: −1.01; 95% CI, −1.40 to −0.61) (Table 2). Comparing the differences in differences in asthma control of participants at 12-month from baseline between groups, only the combination intervention compared with control demonstrated a statistically significant change in asthma control scores (−0.79; 95% CI, −1.4 to −0.18), which exceeded the MCID.

None of the other difference in differences achieved statistical significance. For instance, those receiving the school intervention had a larger magnitude improvement in ACQ score (−0.5; 95% CI, −1.12 to 0.12) comparing those in the combined intervention with primary care intervention alone and −0.5 (95% CI, −1.25 to 0.25) when comparing school intervention alone with the control group. The comparable difference in differences for participants receiving the primary care intervention was −0.29 (95% CI, −1.1 to 0.53) when comparing those receiving the combined intervention with school intervention alone and −0.29 (95% CI, −0.82 to 0.24) when comparing the primary care intervention alone with control.

### Secondary Outcomes

Daytime symptom frequencies were significantly lower in participants receiving the primary care (−1.03; 95% CI, –1.39 to −0.67), school (−0.65; 95% CI, −1.06 to −0.23), and combined (−0.57; 95%, CI, −0.95 to −0.18) interventions compared with control. For nighttime symptoms, only participants in the combined intervention compared with control had a statistically significant lower nighttime symptom burden (−0.38; 95% CI, −0.72 to −0.04) (Table 3). In the stratified analysis of participants who received the intervention before the COVID-19 pandemic, participants who received the primary care intervention had fewer daytime symptoms, including the combined compared with school intervention (−2.31; 95% CI, −3.79 to −0.82), primary care compared with control (−1.54; 95% CI, −2.45 to −0.62), and the combined group compared with control (−2.04; 95% CI, −3.22 to −0.86). In this analysis, nighttime symptoms had a greater reduction in the combined intervention compared with school intervention (−1.55; 95% CI, −2.81 to −0.29) and the combined group compared with control (−1.33; 95% CI, −2.39 to −0.26) (Table 3). There were no statistically significant differences between groups in the other secondary outcomes of emergency department visits, hospitalizations, or asthma-related quality of life (Table 3 and eTables 1 and 2 in Supplement 3).

**Table 3.** Fitted Values for Secondary Outcomes at Baseline and 12 Months^a^^,^^b^

Group	Total No.	Baseline, d	Month 12, d	Month 12 − baseline, d	Difference (95% CI)	*P* value
**Daytime symptoms** ^c^						
P+	308	2.58	1.25	−1.33	−0.62 (−0.91 to −0.34)	<.001
P−	310	2.32	1.61	−0.71		
S+	231	2.43	1.34	−1.09	−0.10 (−0.40 to 0.19)	.49
S−	387	2.46	1.48	−0.99		
S+P+ to S+ alone	120 to 111	2.2 vs 2.69	1.15 vs 1.56	−1.05 vs −1.13	0.08 (−0.39 to 0.55)	.74
S+P+ to P+ alone	120 to 188	2.2 vs 2.82	1.15 vs 1.31	−1.05 vs −1.51	0.46 (0.04 to 0.88)	.03
S+ alone to control	111 to 199	2.69 vs 2.11	1.56 vs 1.63	−1.13 vs −0.48	−0.65 (−1.06 to −0.23)	.002
P+ alone to control	188 to 199	2.82 vs 2.11	1.31 vs 1.63	−1.51 vs −0.48	−1.03 (−1.39 to −0.67)	<.001
S+P+ alone to control	120 to 199	2.2 vs 2.11	1.15 vs 1.63	−1.05 vs −0.48	−0.57 (−0.95 to −0.18)	.004
**Nighttime symptoms** ^d^						
P+	309	1.58	0.84	−0.74	−0.23 (−0.46 to 0.01)	.06
P−	310	1.62	1.1	−0.52		
S+	232	1.67	0.93	−0.74	−0.18 (−0.42 to 0.07)	.16
S−	387	1.56	1.0	−0.57		
S+P+ to S+ alone	120 to 112	1.56 vs 1.79	0.75 vs 1.12	−0.81 vs −0.68	−0.13 (−0.53 to 0.28)	.54
S+P+ to P+ alone	120 to 189	1.56 vs 1.60	0.75 vs 0.89	−0.81 vs −0.71	−0.09 (−0.45 to 0.26)	.60
S+ alone to control	112 to 198	1.79 vs 1.52	1.12 vs 1.09	−0.68 vs −0.43	−0.25 (−0.60 to 0.10)	.16
P+ alone to control	189 to 198	1.60 vs 1.52	0.89 vs 1.09	−0.71 vs −0.43	−0.28 (−0.57 to 0)	.05
S+P+ alone to control	120 to 198	1.56 vs 1.52	0.75 vs 1.09	−0.81 vs −0.43	−0.38 (−0.72 to −0.04)	.03
**No. of emergency department visits** ^e^						
P+	313	1.21	0.58	−0.63	−0.07 (−0.29 to 0.15)	.55
P−	313	1.20	0.64	−0.57		
S+	233	1.22	0.52	−0.70	−0.16 (−0.39 to 0.06)	.16
S−	393	1.20	0.66	−0.54		
S+P+ to S+ alone	121 to 112	1.11 vs 1.35	0.50 vs 0.55	−0.61 vs −0.80	0.19 (−0.17 to 0.55)	.31
S+P+ to P+ alone	121 to 192	1.11 vs 1.28	0.50 vs 0.63	−0.61 vs −0.65	0.03 (−0.28 to 0.35)	.83
S+ alone to control	112 to 201	1.35 vs 1.12	0.55 vs 0.69	−0.80 vs −0.44	−0.36 (−0.69 to −0.03)	.03
P+ alone to control	192 to 201	1.28 vs 1.12	0.63 vs 0.69	−0.65 vs −0.44	−0.21 (−0.49 to 0.07)	.14
S+P+ alone to control	121 to 201	1.11 vs 1.12	0.50 vs 0.69	−0.61 vs −0.44	−0.18 (−0.48 to 0.13)	.26
**No. of hospitalizations** ^f^						
P+	313	0.45	0.15	−0.31	−0.01 (−0.13 to 0.12)	.90
P−	313	0.42	0.12	−0.30		
S+	233	0.40	0.16	−0.24	0.09 (−0.04 to 0.22)	.16
S−	393	0.46	0.12	−0.34		
S+P+ to S+ alone	121 to 112	0.37 vs 0.44	0.17 vs 0.14	−0.20 vs −0.30	0.10 (−0.10 to 0.30)	.33
S+P+ to P+ alone	121 to 191	0.37 vs 0.51	0.17 vs 0.13	−0.20 vs −0.38	0.18 (−0.001 to 0.36)	.05
S+ alone to control	112 to 201	0.44 vs 0.41	0.14 vs 0.11	−0.30 vs −0.30	0.001 (−0.18 to 0.19)	.97
P+ alone to control	192 to 201	0.51 vs 0.41	0.13 to 0.11	−0.38 vs −0.30	−0.08 (−0.24 to 0.09)	.36
S+P+ alone to control	121 to 201	0.37 vs 0.41	0.17 vs 0.11	−0.20 vs −0.30	0.10 (−0.07 to 0.27)	.24

Abbreviations: P+, primary care community health worker; P−, usual care; S+, community health worker–led school intervention; S−, usual care in schools.

^a^ Longitudinal models for the primary and secondary outcomes.

^b^ eFigures 2 to 15 in Supplement 2 visually display the results in this table. There has been no adjustment for the COVID-19 pandemic phase.

^c^ Daytime symptoms in past 2 weeks, at 0 and 12 months: mixed-effects generalized binomial model (n = 14) with random intercepts for child. The model with random intercepts for both child and school did not converge, and the models included time (day of follow-up), indicator variables for each intervention, and time by intervention interaction terms.

^d^ Nighttime symptoms in past 2 weeks, at 0 and 12 months: mixed-effects generalized binomial model (n = 14) with random intercepts for child. The model with random intercepts for both child and school did not converge, and the models included time (day of follow-up), indicator variables for each intervention, and time by intervention interaction terms.

^e^ Emergency department visits 1 year prior, at 0 and 12 months: mixed-effects negative binomial model (n = 365) with random intercepts for child. The model with random intercepts for both child and school did not converge, and the models included time (day = 0 or 365), indicator variables for each intervention, and time by intervention interaction terms.

^f^ Hospitalizations 1 year prior, at 0 and 12 months: mixed-effects generalized binomial model (n = 365) with random intercepts for child. The model with random intercepts for both child and school did not converge, and the models included time (day = 0 or 365), indicator variables for each intervention, and time by intervention interaction terms.

## Discussion

This was a large, randomized clinical trial designed to understand the potential benefits of CHW-led, evidence-based interventions implemented in the primary care, school, and combined primary care and school settings for children with asthma. In the context of a factorial design, CHWs implemented 4 evidence-based interventions, coordinated either in primary care or school settings, that had reach into the home and community settings. Although previous studies have shown success in using CHWs to implement interventions for asthma in single settings, our study offers insight into how and where CHWs can enhance seamless integration across primary care and school settings.^9,10,17,32^

Although all treatment groups showed improvement in asthma control at 12 months, with active intervention groups (primary care alone, school alone, and combined intervention) demonstrating statistically significant and clinically meaningful improvements over time, none of the differences between study groups over time (difference in differences) met these thresholds for our primary model. The subanalysis that compared the groups who received the interventions before the substantial COVID-19–related adaptations, however, demonstrated the greatest absolute improvements in asthma control, meeting criteria for both statistical significance and clinical meaningfulness for the combined intervention group when compared with control. Statistically significant improvements were also observed in secondary outcomes of daytime symptoms and nighttime symptoms for the primary care, school, and combined intervention groups when compared with controls, with the largest magnitude reductions in the subgroup with the 12-month visit before shutdown for the pandemic. The fact that the greatest improvements in asthma control were observed in the group who received the intervention as designed (pre–COVID-19 pandemic disruptions), with a greater impact on asthma control in the groups, which included the school interventions, suggests potential synergies in integrating health care, school, and home in order to improve asthma control. Asthma management is initiated in the health care setting, which is often disconnected from home and school environments where school-aged children with asthma spend most of their time.^10,23,33,34^ This study’s intention was to support best practices in asthma care in all environments, connecting health care through CHWs, reflecting real-world experiences of children with asthma. This study also demonstrated the value of facilitating medication delivery directly to schools by pharmacy. This was an innovative approach that allowed for adaptations to support our community.

### Limitations

The impact of COVID-19 pandemic on this study cannot be overemphasized. Given the centrality of a CHW-delivered school intervention, where CHWs taught asthma classes to identify classroom asthma triggers and conducted directly observed therapy delivered in school nurses offices, necessary study adaptations for virtual and hybrid schooling altered these interventions so dramatically that they should not be considered the evidence-based interventions the study was designed to evaluate. Although similar technology-enhanced adaptations were made to the primary care intervention, virtual home visits proved more feasible than conducting virtual asthma classes and virtually observed medication monitoring. Additionally, during the COVID-19 pandemic, acute care visits for asthma decreased dramatically for all ages and asthma control improved,^31,35^ which led to difficulties with identification of eligible children. This may have led to ceiling effects in our primary outcome analysis, as evidenced by 47% of children had well-controlled asthma at baseline.

An additional limitation is that health care utilization is only captured by the CHOP EHR and those that sought services elsewhere would not have been captured. We expect that this would be a relatively small number because we recruited children who lived in the proximal community surrounding CHOP. Finally, this study was completed in an inner-city, poorly resourced community and may not be generalizable to other populations.

## Conclusions

Despite interruptions in the interventions brought on by COVID-19 pandemic, the results of this randomized clinical trial provide initial evidence that connecting all environments in caring for children with asthma was both feasible and potentially effective. Having additional support in the school environment may enhance the benefits previously observed when CHWs deliver education and care coordination in the clinic and home environments. CHWs provide additional support in both primary care and school settings to help overcome barriers to medication administration, coordination of paperwork, and communications with clinical staff in the primary care and school settings. Our findings should encourage stakeholders—including health systems, schools, and payers—to engage in cross-sector CHW-based school-clinic linkages to assess design characteristics and implementation factors associated with improved asthma outcomes.

## Notes

Supplement 1.
Trial Protocol.

Supplement 2.
Statistical Analysis Plan.

Supplement 3. **eFigure 1.** Graphs of Fitted Values of Asthma Control vs Planned Month of Visit **eFigure 2.** Graph of Fitted Values of Daytime Symptoms in Past 2 Weeks vs Planned Month of Visit **eFigure 3.** Graph of Fitted Values of Nighttime Symptoms in Past 2 Weeks vs Planned Month of Visit **eFigure 4.** Graphs of Fitted Values of Number ED Visits in Previous 365 Days vs Planned Month of Visit **eFigure 5.** Graphs of Fitted Values of Number Hospital (HOSP) Visits in Previous 365 days vs Planned Month of Visit **eFigure 6.** Graphs of Fitted Values of Quality of Life (Emotional) vs Planned Month of Visit **eFigure 7.** Graphs of Fitted Values of Quality of Life (Activity) vs Planned Month of Visit **eFigure 8.** Graphs of Fitted Values of Number Steroid Courses in Previous 365 days vs Planned Month of Visit **eFigure 9.** Graph of Fitted Values of Daytime Symptoms in Past 2 weeks vs Planned Month of Visit **eFigure 10.** Graph of Fitted Values of Nighttime Symptoms in Past 2 weeks vs Planned Month of Visit **eFigure 11.** Graphs of Fitted Values of Number ED Visits in Previous 365 days vs Planned Month of Visit **eFigure 12.** Graphs of Fitted Values of Number HOSP Visits in Previous 365 days vs Planned Month of Visit **eFigure 13.** Graphs of Fitted Values of Asthma Control vs Planned Month of Visit **eFigure 14.** Graphs of Fitted Values of Quality of Life (Activity) vs Planned Month of Visit **eFigure 15.** Graphs of Fitted Values of Number Steroid Courses in Previous 365 days vs Planned Month of Visit **eTable 1.** Fitted Values for Secondary Outcomes at Baseline and 12 Months **eTable 2.** Fitted Values of Secondary Outcomes at 12 Months, Stratified by Study Completion Before the COVID-19 Pandemic Phase

Supplement 4.
Data Sharing Statement.
