# Supplementary material for: Community Health Workers Linking Clinics and Schools and Asthma Control: A Randomized Clinical Trial
Source: JAMA Pediatr. 2024 Oct 21;178(12):1260–9. doi: 10.1001/jamapediatrics.2024.3967 (PMC11581744; doi:10.1001/jamapediatrics.2024.3967)
Supplement: Supplement 1. — Trial Protocol. [file jamapediatr-e243967-s001.pdf]

Title:	**West Philadelphia Asthma Care Implementation Plan**
Short Title:	West Philadelphia Controls Asthma
Regulatory Sponsor:	NHLBI
eIRB Number:	17-013892
Protocol Date:	September 01, 2016 — Version #: 9.0

Amendment 1 Date: March 29 2017

Amendment 2 Date: April 14 2017

Amendment 3 Date: December 18, 2017

Amendment 4 Date: April 23, 2018

Amendment 5 Date: June 4, 2018

Amendment 6 Date: September 29, 2018

Amendment 7 Date: April 22, 2019

Amendment 8 Date: June 24, 2019

Amendment 9 Date: August 15, 2019

Amendment 10 Date: November 15, 2019

Amendment 11 Date: March 19, 2020

Amendment 12 Date: November 13, 2020

Amendment 13 Date: April 19, 2021

Funding Sponsor

NHLBI
National Heart, Lung, and Blood Institute
9000 Rockville Pike
Bethesda, Maryland 20892
United States

Principal Investigator

Tyra Bryant-Stephens, MD
Children's Hospital of Philadelphia
2716 South Street, 9th Floor
Philadelphia, PA 19146
Phone 215-590-5020
email: stephenst@email.chop.edu

TABLE OF CONTENTS

Table of Contents ii
Abbreviations and Definitions of Terms iv
Abstract v
Protocol Synopsis vi
Table 1: Schedule of Study Procedures ix
Figure 1: WEPACC Model Study Design x
Figure 2: Logic Model for Study Design xi
Figure 3: Risk Mapping xii

1 BACKGROUND INFORMATION AND RATIONALE 1
1.1 INTRODUCTION 1
1.2 RELEVANT LITERATURE AND DATA 4
1.3 COMPLIANCE STATEMENT 5

2 STUDY OBJECTIVES 5
2.1 PRIMARY AIM 5
2.2 SECONDARY AIMS 5

3 INVESTIGATIONAL PLAN 6
3.1 GENERAL SCHEMA OF STUDY DESIGN 6
3.1.1 Screening Phase *7*
3.1.2 Primary Care Based Intervention *7*
3.1.3 School Based Intervention *8*
3.2 ALLOCATION TO TREATMENT GROUPS AND BLINDING 20
3.3 STUDY DURATION, ENROLLMENT AND NUMBER OF SITES 9
3.3.1 Duration of Study Participation *9*
3.3.2 Total Number of Study Sites/Total Number of Subjects Projected *10*
3.4 STUDY POPULATION. 10
3.4.1 Inclusion Criteria *10*
3.4.2 Exclusion Criteria *10*

4 STUDY PROCEDURES 11
4.1 SCREENING VISIT 21
4.2 STUDY TREATMENT PHASE 21
4.2.1 Visit 1 (All Participants) *11*
4.3 PRIMARY CARE BASED INTERVENTION: 11
4.3.1 Study Visit 1 (Enrollment) *11*
4.3.2 Study Visit 2 (Home Visit 1): *23*
4.3.3 Study Visit 3 (Home Visit 2) *23*
4.3.4 Study Visit 4 (Home Visit 3) *13*
4.3.5 Study Visits 5, 6, and 7 (Primary Care Office Visits 1-3) *13*
4.3.6 Study Visit 8 (12-Month Follow-Up) *24*
4.3.7 Study Visit 9 (Post 12-Month Follow-Up) *14*
4.3.8 Unscheduled Visits *15*
4.4 SCHOOL INTERVENTION 15
4.4.1 Community Health Worker Coordination *15*
4.4.2 School Nurse Coordination *15*
4.4.3 Classroom Environmental Assessments *15*
4.4.4 Open Airways for Schools Curriculum *25*
4.4.5 School Based Asthma Therapy *25*
4.5 RESCUE MEDICATION ADMINISTRATION 16
4.6 SUBJECT COMPLETION/WITHDRAWAL 16

4.6.1 Early Termination Study Visit ... *16*

5 STUDY EVALUATIONS AND MEASUREMENTS ... **26**

5.1 INDIVIDUAL PARTICIPANT MEASURES ... 26
5.2 GROUP LEVEL PRIMARY CARE AND SCHOOL MEASURES ... 17
5.3 SCREENING AND MONITORING EVALUATIONS AND MEASUREMENTS ... 27
5.3.1 Medical Record Review ... *27*
5.3.2 Physical Examination ... *18*
5.3.3 Vital Signs ... *18*
5.4 EFFICACY EVALUATIONS ... 18
5.4.1 Diagnostic Tests, Scales, Measures, etc. ... *28*
5.5 SAFETY EVALUATION ... 28

6 STATISTICAL CONSIDERATIONS ... **19**

6.1 PRIMARY ENDPOINT ... 29
6.2 SECONDARY ENDPOINTS ... 29
6.3 STATISTICAL METHODS ... 29
6.3.1 Baseline Data ... *29*
6.3.2 Efficacy Analysis ... *21*
6.3.3 Subgroup Analysis: Mediator and Moderator Variables ... *21*
6.4 SAMPLE SIZE AND POWER ... 29
6.5 INTERIM ANALYSIS ... 30

7 STUDY MEDICATION (STUDY DEVICE OR OTHER STUDY INTERVENTION) ... **31**

7.1 DESCRIPTION ... 31

8 SAFETY MANAGEMENT ... **31**

8.1 CLINICAL ADVERSE EVENTS ... 31
8.2 ADVERSE EVENT REPORTING ... 23

9. STUDY ADMINISTRATION ... **31**

9.1 TREATMENT ASSIGNMENT METHODS ... 31
9.1.1 Randomization ... *31*
9.1.2 Blinding ... *31*
9.1.3 Unblinding ... *24*
9.2 DATA COLLECTION AND MANAGEMENT ... 32
9.3 CONFIDENTIALITY ... 32
9.4 REGULATORY AND ETHICAL CONSIDERATIONS ... 32
9.4.1 Data and Safety Monitoring Plan ... *32*
9.4.2 Risk Assessment ... *24*
9.4.3 Potential Benefits of Trial Participation ... *33*
9.4.4 Risk-Benefit Assessment ... *33*
9.5 RECRUITMENT STRATEGY ... 33
9.6 INFORMED CONSENT/ASSENT AND HIPAA AUTHORIZATION ... 33
9.7 PAYMENT TO SUBJECTS/FAMILIES ... 34
9.7.1 Payments to parent for time, effort and inconvenience (i.e. compensation) ... *34*
9.7.2 Gifts ... *34*

10 PUBLICATION ... **34**

11 REFERENCES ... **27**

Appendix ... **37**

ABBREVIATIONS AND DEFINITIONS OF TERMS

CAPP	Community Asthma Prevention Program
ED	Emergency Department (Admission)
IP	Inpatient Admission
CHW	Community Health Worker
EHR	Electronic Health Record
SC	Study Coordinator
PCP	Primary Care Provider
RA	Research Assistant
SBAT	School Based Asthma Therapy
EBI	Evidence Based Intervention
CBM	Center for Biomedical Informatics
HV	Home Visit
REDCap	Research Electronic Data Capture (Web-based Survey Platform)
NHLBI	The National Heart, Lung, and Blood Institute
DSMB	Data Safety Monitoring Board
DSMP	Data and Safety Monitoring Plan
FQHC	Federally Qualified Health Center
NIH	National Institutes of Health
PAMCOP	Pediatric and Adolescent Medicine Centers of Philadelphia

ABSTRACT

Context:

Childhood asthma is characterized by prominent, persistent and pervasive disparities. For instance, in addition to often cited disparities in specific health outcomes and global quality of life, students with asthma miss three times more school days than students without asthma with the highest proportion of missed days in low-income communities. To address asthma disparities in a patient-centered, sustainable manner, and consistent with a social ecological framework, one must intervene in all environmental contexts (i.e., home, school, healthcare system, community) of children with asthma. As a result of an assessment of local needs, resource mapping, and months of planning, an asthma care implementation program has been developed with the broad objective of integrating home, school, healthcare system, and community for school-aged asthmatic children in West Philadelphia.

Objective: To determine if the utilization of CHWs in a comprehensive community-based intervention to connect home, school, healthcare system and community for low-income minority school-aged asthmatic children and their caregivers improves asthma control and thereby reduces asthma disparities for this community.

Study Design: Randomized controlled trial

Setting/Participants:

640 asthmatic children (ages 5-13 years) will be recruited from CHOP Care Network sites (Cobbs Creek, Karabots, and South Philadelphia), CHOP ED, The Pediatric and Adolescent Medicine Centers of Philadelphia, Federally Qualified Health Centers and by community referral.

Other sites of intervention include: All School District of Philadelphia public elementary schools located in West Philadelphia will be invited to participate. Several public charter elementary schools in West Philadelphia some of which are served by school-based health centers managed by Education Plus, Inc. d/b/a Education Plus Health will also be invited to participate for a total of no greater than 40 elementary schools.

Study Interventions and Measures:

Interventions taking place at school, home and in the primary care setting to improve asthma control and reduce symptom days evaluated using main and simple effects from the factorial design.

PROTOCOL SYNOPSIS

**Study Title**	West Philadelphia Asthma Care Implementation Plan
**Funder**	NIH
**Clinical Phase**	Implementation Phase
**Study Rationale**	The overall purpose of care coordination is to identify and engage families in development of a care plan that links clinical, psychosocial and other services in order to provide best care to the child with asthma. It is essential that care coordination emphasizes the central role of the health of the child while recognizing family concerns and identifying strengths and gaps in care. In care coordination, it is important to establish and support a continuous relationship with clinical caregivers as well as empower families to advocate for their needs and satisfaction.<br><br>Through a combination of asthma management interventions for children including, Yes We Can™ Children's Asthma Program, School Based Asthma Therapy, and the American Lung Association's Open Airways For Schools®, utilizing CHWs as the connectors, families of children with asthma will receive services of a trained and knowledgeable liaison to proactively support the delivery of evidence based care and follow-up in the primary care office, school and at home.
**Study Objective(s)**	**Primary**<br>To improve asthma control through the use of Community Health Workers that provide a network of education and care coordination support, and to facilitate communication for families of children with asthma in West Philadelphia by integrating home, school, healthcare system, and community for school-aged asthmatic children in West Philadelphia.<br><br>**Secondary**<br>To reduce asthma symptom days and school absences through the use of CHWs, evaluate cost-effectiveness of study interventions, and to investigate moderators of intervention effectiveness to identify subgroups of children that may be more likely to benefit from the study interventions.
**Test Article(s)** *(If Applicable)*	N/A
**Study Design**	Randomized controlled trial

**Subject Population key criteria for Inclusion and Exclusion:**	**Inclusion Criteria**<br>1. Parents (guardians) and their children ages 5-13 years<br>2. Children with diagnosis of asthma<br>3. Uncontrolled Asthma (as evidenced by one or more asthma exacerbations requiring oral steroids within the past year, two ED visits for asthma or one inpatient admission for asthma within the past year)<br>4. Reside in one of the seven West Philadelphia zip codes: 19104, 19131, 19139, 19142, 19143, 19151, 19153<br>5. Enrolled in elementary school<br>6. Receive primary care at CHOP Care Network sites (Cobbs Creek, Karabots, and South Philadelphia), The Pediatric and Adolescent Medicine Centers of Philadelphia (PAMCOP) or a Federally Qualified Health Center (FQHC) in West Philadelphia<br>7. Parental/guardian permission (informed consent and if appropriate, child assent)<br>8. English speaker<br><br>**Exclusion Criteria**<br>1. Children with other chronic respiratory illnesses such as cystic fibrosis<br>2. Cyanotic congenital heart disease<br>3. MRCP-Mental retardation and/or cerebral palsy<br>4. Severe Neurological Disorder<br>5. Parents/guardians or children who, in the opinion of the Investigator, is likely to be non-compliant with study schedules or procedures.<br>6. Children who do not live in West Philadelphia (zip codes: 19104, 19131, 19139, 19142, 19143, 19151, 19153)
**Number Of Subjects**	640 patients will be recruited from CHOP Care Network sites, a private pediatrician office, Federally Qualified Health Centers (FQHCs), CHOP ED and by community referral.<br><br>Sites Include:<br>• CHOP Care Network sites: Cobbs Creek, Karabots and South Philadelphia<br>• CHOP Emergency Department<br>• Federally Qualified Health Centers (FQHCs) serving West Philadelphia residents<br>• The Pediatric and Adolescent Medicine Centers of Philadelphia (PAMCOP) serving West Philadelphia residents

	• School District of Philadelphia Public Elementary Schools located in West Philadelphia (including Southwest Philadelphia) who agree to participate<br>• Public Charter Schools located in West Philadelphia (including Southwest Philadelphia) who agree to participate
**Study Duration**	Active subject participation will last up to 24 months; Medical record review will continue for up to two years from the 12-month follow-up visit or until the data collection period for the study has ended, whichever occurs first. This study is expected to be conducted over six years.
**Study Phases**	Screening<br>Study Intervention<br>Study Follow-up
**Efficacy Evaluations**	Obtain baseline and follow-up measurements of asthma control, symptoms, patient and caregiver asthma knowledge, presence of environmental triggers, school and work day absenteeism, and healthcare utilization
**Safety Evaluations**	Adverse event reports will be collected at each visit. These reports will be reviewed at weekly meetings by the PI to determine if they are a result of the study.<br>A DSMB has been appointed by the funding sponsor to oversee safety of the interventions.
**Statistical And Analytic Plan**	Analysis to be conducted by biostatisticians at the University of Pennsylvania and data managers at the Biostatistics and Data Management Core at CHOP
**DATA AND SAFETY MONITORING PLAN**	The PI will be responsible for the study's Data and Safety Monitoring Plan with oversight provided by a DSMB appointed by the NIH.

TABLE 1: SCHEDULE OF STUDY PROCEDURES

**Study Phase**	**Screening**		**Treatment/Intervention**			**Follow-Up**					
		Baseline	Home Visits			Clinic Visits 3, 6, and 9 Mo			12 Mo	12 Mo	Post-12 Mo
Visit Number	0	1	HV1	HV2	HV3	2	3	4	HV4	5	6
Informed Consent/Assent	X										
Baseline Questionnaire with Asthma Symptom Recall		X									
Photograph of child's bedroom			X		X				X		
Clinical Assessment – Vital Signs and BMI		X				X	X	X		X	
Pulmonary Function Test		X				X	X	X		X	
Metered Dose Inhaler (MDI) Checklist		X		X		X	X	X		X	
Paediatric Asthma Caregiver's Quality of Life Questionnaire		X								X	
Asthma Control Questionnaire		X				X	X	X		X	X
Child Asthma Risk Assessment Tool (CARAT) modified		X								X	
Initial Home Environmental Assessment			X								
Asthma Knowledge Quiz			X		X				X	X	
Caregiver Needs Assessment		X									
Community Health Worker Goal Tracking		X	X	X	X	X	X	X	X	X	
Asthma Management Education			X	X	X	X	X	X	X		
Follow-Up Questionnaire with Asthma Symptom Recall						X	X	X		X	X
Follow-Up Home Environmental Assessment				X	X				X		
School Nurse Checklist		X								X	
Initial Classroom Environmental Assessment		X									
Follow-Up Classroom Environmental Assessment						X					
School Based Asthma Therapy Intake Survey		X									
CHW Caregiver Satisfaction Survey											X

x

FIGURE 1: WEPACC MODEL STUDY DESIGN

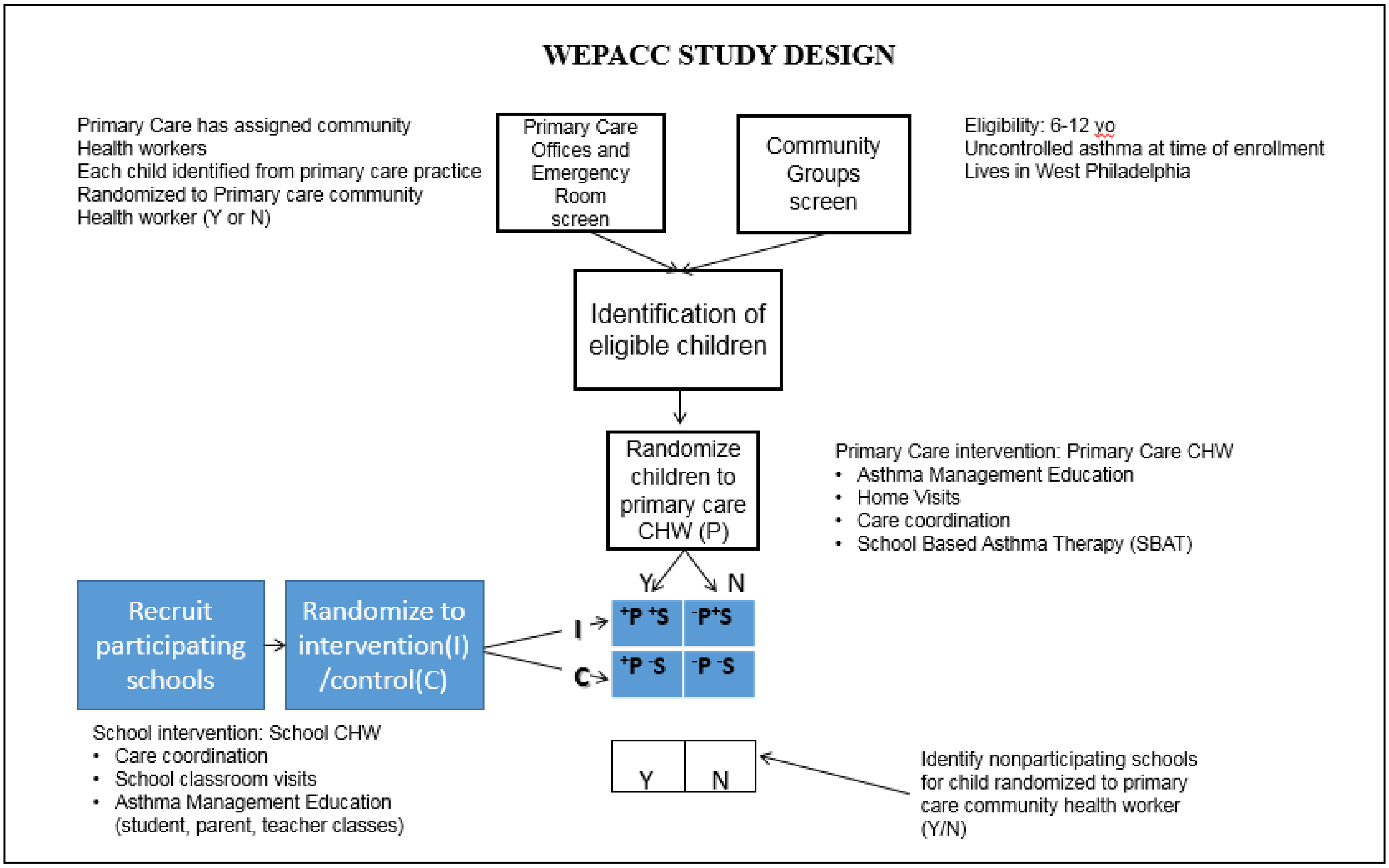

FIGURE 2: LOGIC MODEL FOR STUDY DESIGN

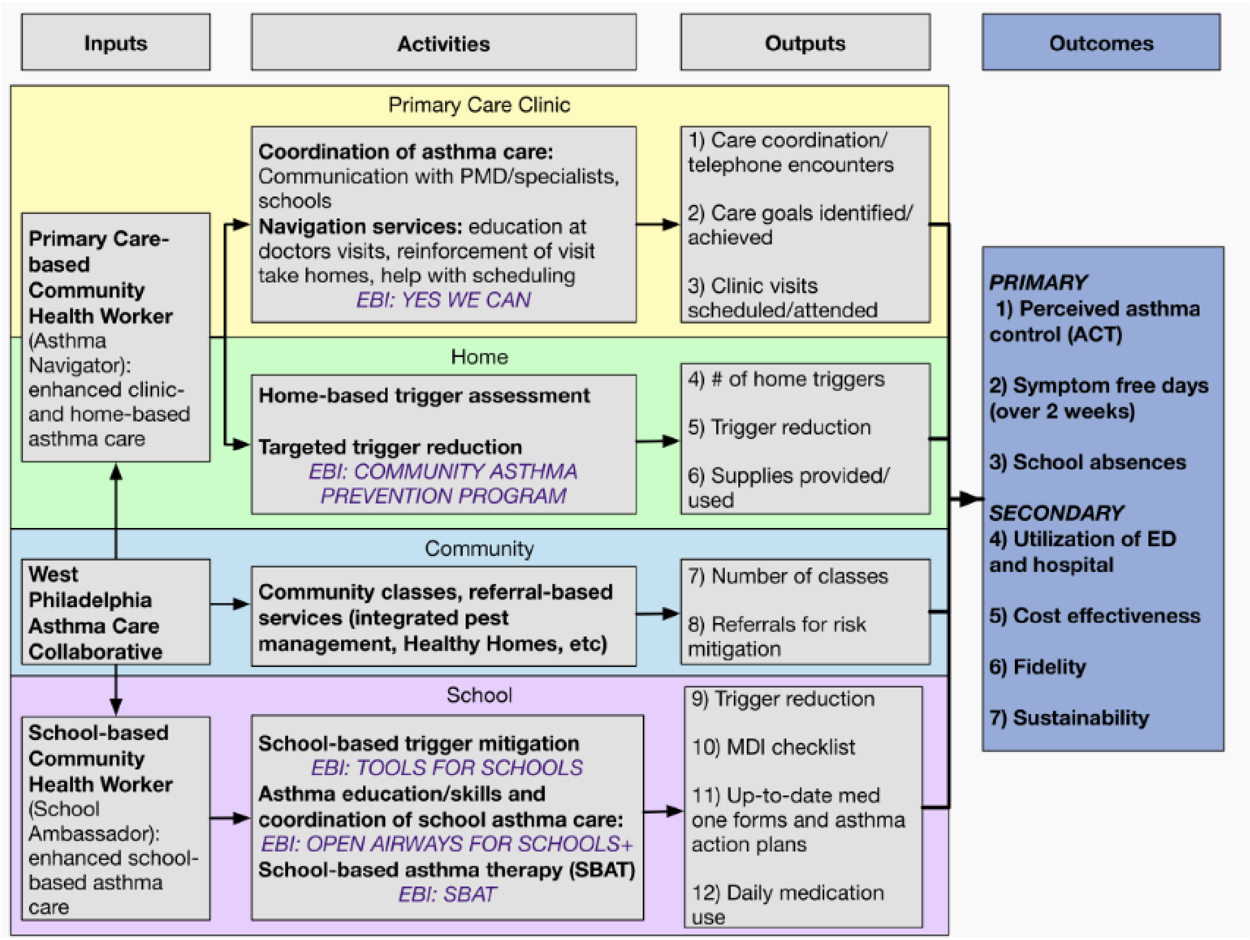

FIGURE 3: RISK MAPPING

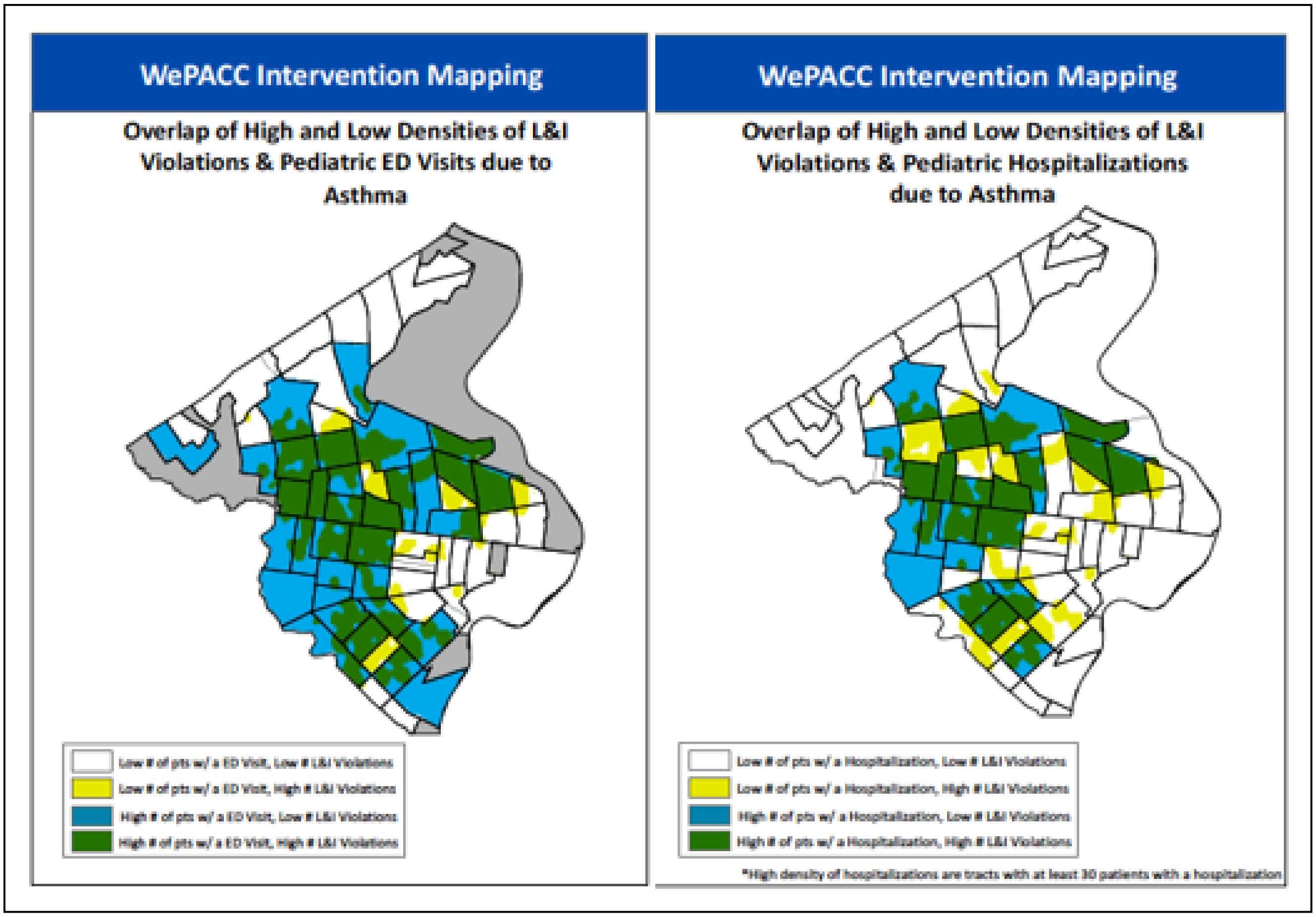

1 Background Information and Rationale

2 Introduction

Childhood asthma is characterized by prominent, persistent and pervasive disparities. For instance, in addition to often cited disparities in specific health outcomes and global quality of life[1–4], students with asthma miss three times more school days than students without asthma, with the highest proportion of missed school days in low-income communities[5]. To address asthma disparities in a patient-centered, sustainable manner, and consistent with an ecological framework, one must intervene in all environmental contexts (i.e., home, school, healthcare system, community) of children with asthma. Previous studies demonstrate that community health workers (CHWs) can effectively deliver evidence-based care coordination, environmental mitigation, and asthma education interventions in specific settings[6–8]. However, this work has not been delivered in an integrative manner to connect settings. Utilization of CHWs in a comprehensive community-based intervention to connect home, school, healthcare system and community for school-aged asthmatic children and their caregivers may eliminate asthma disparities.

The Community Asthma Prevention Program (CAPP) has a two decade history of utilizing CHWs to improve asthma outcomes of children in Philadelphia[9,10]. Over ten members of inner-city Philadelphia have been trained and employed as CHWs to effectively provide home asthma education and environmental mitigation for children who have asthma-related ED or hospital visits. Building on this foundation, we established a network of stakeholders, The West Philadelphia Asthma Care Collaborative (WEPACC), with representation from public housing, healthcare, community, and schools. As a result of local needs assessment, resource mapping, and months of planning, we designed an asthma care implementation program with the broad objective of integrating home, school, healthcare system, and community for school-aged asthmatic children in West Philadelphia. We will accomplish this goal using CHWs to deliver sustainable patient-centered evidence-based interventions. The evidence-based interventions include (1) a primary care-based Yes We Can™ Children's Asthma Program with home visitation and (2) a comprehensive and rigorously evaluated school-based intervention, Open Airways For Schools® and (3) School Based Asthma Therapy[11–13]. CHWs will function as the hub of each intervention, serving either as primary care CHWs or school CHWs to provide a network of education, care coordination support, and to facilitate communication for families of children with asthma between the four sectors. If proven effective, this care coordination program will assist caregivers in assessing resources, improving self-management skills and ultimately reducing asthma-related ED visits and hospitalizations.

In the face of best asthma practices, quality of care is also affected by communication between patients and providers, particularly across cultures. When people of color access routine care, physicians are often unknowingly biased in their care. This may partially be explained by different styles of communicating between providers and patients, different belief systems and unfamiliarity with the patient/caregivers social stressors which affect their ability to be adherent with treatment. Giving the caregiver a central health liaison who is also a resident in the community such as the CHW, will help to bridge communication gaps between caregiver and clinician, identify belief systems and enable caregivers to deal with social stressors.

Another example of current system limitations is that of our own CAPP program. Although many have benefited from these programs, high risk families need a higher intensity of service beyond the type of home visiting program CAPP provides which is focused on health education and environmental mitigation in the home without making a formal connection to health care and schools.

Strunk and colleagues found that diverse ethnic and racial minorities experienced disparities in access to asthma care that lead to greater morbidity and mortality[14]. Absence of regular care for urban African American children results in increased morbidity for chronic diseases like asthma. Urban children have more difficulty with self-management of their asthma for various reasons, which include poverty, stresses related to living in an urban environment, lack of knowledge about asthma and lack of family and community support. Many studies reveal that asthma education is effective in

reducing emergency department visits, hospitalizations and lost days of work. Improving access to care not only involves changing the structure of health care delivery, but also involves addressing educational needs, psychological issues, and cultural beliefs and practices in developing interventions to improve access to care.

Community Needs Assessment. In order to understand the impact of asthma in West Philadelphia, our needs assessment included both qualitative inquiry (i.e., focus groups and interviews) as well as identification of key local, regional and national data sources for secondary data analysis conducted by Philadelphia Health Management Corporation (PHMC) under the guidance of WEPACC. Knowledge of the neighborhoods relevant to WEPACC informed our intervention planning and implementation.

Results from secondary analysis of data. Population. West Philadelphia (zip codes 19104, 19131, 19139, 19143, and 19151) has 233,000 residents, including 49,000 ages 0-17. Seventy-four percent of West Philadelphia residents identify as Black/African American. Thirty-two percent of children live below the federal poverty level, 98% of children have health insurance, and 100% of parents reported their child receives regular healthcare[15]. This high access to healthcare suggests that an intervention involving partnerships with healthcare providers has the potential to reduce the impact of asthma in West Philadelphia. Twenty-five percent of the children in these zip codes have asthma[16]. CHOP is the primary source of pediatric emergency and hospital care for West Philadelphia and provides primary care for over 40,000 children in West Philadelphia.

Utilization. 1,353 children residing in West Philadelphia were hospitalized for asthma, and 3,186 children were admitted to CHOP's emergency room for asthma in 2014-2015[17]. The concentration of hospitalizations and emergency room discharges is relatively consistent across West Philadelphia. Notably, the WEPACC pilot zone, outlined on both maps, is interior to the overall WEPACC ZIP codes, and contains census tracts with both high numbers of asthma hospitalizations and ED discharges.

Community Resources. Community assets were mapped to inform the design of the intervention with input from WEPACC stakeholders.

Risk Factors. Since poor housing conditions are associated with asthma morbidity, we mapped housing code violations and overlapped them with CHOP hospitalization/emergency room data to determine areas of highest risk. The resulting map revealed that the highest risk areas centered around three primary care centers which are the sites of enrollment for our study (Figure 3).

Qualitative Assessment. Under the guidance of the West Philadelphia Asthma Care Collaborative (WEPACC), Philadelphia Health Management Corporation (PHMC) collected qualitative data from focus groups and key informant interviews in order to assess the needs of children with asthma in West Philadelphia. A total of five focus groups were convened between November 2015 and April 2016. Three groups consisted of parents of children with asthma (n=24), one group with children with asthma (n=7), and one group with school nurses (n= 7). A total of seven key informant (KI) interviews were conducted between May 2016 and July 2016. Participants included parents of children with asthma, health care providers, and an insurance company representative. Needs and concerns were identified from all environmental contexts, home, school, healthcare system, and community. Home concerns included landlord unresponsiveness, difficulty with controlling triggers such as second hand smoke exposure and mold, parental mental health issues, children living in multiple homes, and difficulty using medication. School nurse concerns included the lack of full time nurses, lack of asthma education, poor communication, indoor triggers in the school, missed school days due to asthma, and incomplete med-1 forms. Healthcare concerns were of lack of understanding about asthma, poor communication between parent and provider, missed appointments, medication use and controlling child's asthma. Lastly, lack of awareness about asthma, uncontrollable outdoor triggers like air pollution and secondhand smoke were identified as major concerns in the community setting.

Piloting the school intervention. As we conducted the needs assessment, we also began developing our plan for implementation strategies, or the "how" of implementation (i.e., the active methods to increase adoption, implementation and sustainment of evidence-based intervention)[18]. Intervention mapping is a systematic process for developing interventions to inform implementation strategies that comes out of a participatory design approach[19]. Mapping has been used to develop a number of effective health-related programs, including those addressing sex education, obesity, and cancer prevention[20–22]. We applied mapping to the development of the implementation strategies to be used to implement school-based asthma care.

CHOP Care Network Cobbs Creek, Karabots and South Philadelphia locations each employ a CHW who conduct care coordination and home visits. However, these three CHWs are not able to serve all persistent asthmatics in the practice and do not connect directly with schools to coordinator care for children with asthma. Since our needs assessment revealed caregiver and school nurse concerns regarding asthma management in schools, we focused our pilot on enhancing the connection of CHWs to a local elementary school in order to explore challenges and opportunities for CHWs in elementary school. Thus we piloted the feasibility of a CHW coordinating school asthma care for seven students receiving primary care through CHOP CN Cobbs Creek. After obtaining consent from the parents, the CHW worked directly with the school nurse, principal, and teacher in order to implement elements of the school intervention to improve communication between caregiver, clinical team and school. The CHW enrolled patients at CHOP CN Cobbs Creek and then coordinated with the primary care physician completion of all necessary forms and appropriate medication/device orders. Despite initial challenges, such as incomplete forms, poor communication between caregiver and nurse, no medications on site, our CHW was able to complete the initial intervention for all 7 children within three weeks of enrollment. Subsequent key informant interviews with the caregivers, school nurse and CHW were overall positive with caregivers and school nurses appreciative of the role of the CHW who coordinated with caregiver and school nurse to make sure that the school had forms, medication and devices needed to provide asthma care for enrolled students. With this success we believe our study design to put CHWs in each of the intervention schools is feasible and the best option for closing gaps and connecting asthma care for school-aged children.

3 Relevant Literature and Data

CHWs can improve outcomes for chronic diseases including asthma. Community Health Workers integrated into the health team is supported by the Affordable Care Act[23]. As community residents they are uniquely situated in the community and public health and can serve as the connectors between the two[24]. In the primary care setting, community health workers (CHWs) can enhance the three principles of the medical home: whole person orientation, care coordination, and quality and safety by (1) ensuring that the caregiver's voice is heard regarding asthma, medications, and stressors,[25–27] 2) creating opportunities for the caregiver to "teach back" 3) facilitating communication between the caregiver and primary care provider/specialist, and 4) helping caregivers make and reach goals for asthma management[28–30]. Primary care-based CHWs can promote adherence to asthma guidelines for asthma control, care plan completion and spirometry. CHWs can also facilitate communications when caregivers encounter barriers to scheduling and obtaining medications and devices, or need advocacy for psychosocial issues[31–33]. In addition CHWs delivering multi-component trigger reduction interventions effectively improve asthma symptoms[34]. CHW delivered asthma classes in schools have resulted in improved asthma knowledge and self-management skills[35,36].

There is great potential for CHWs to connect the four sectors where children live. The potential synergistic strength of integrating interventions across multiple sectors (home, school, community, healthcare system) has not been fully leveraged despite knowledge that multi-level interventions are most effective[34]. Currently asthma evidence-based interventions (EBI) are routinely implemented in either the home, school, or primary care setting. Even when caregivers of poor children are engaged in asthma interventions in one setting, they often have to navigate challenges in another setting, such as an under-resourced home, non-supportive school, or disengaged health care provider. In one study a

comprehensive school management program to promote care coordination and better asthma management conducted by school nurses did not succeed because it placed additional responsibilities on the school nurse and there was no buy in from the primary care practice[37]. CHWs providing the connection between the home, school and primary care practice may reduce the burden on caregivers and facilitate asthma management for school-aged children. Further, CHWs, as community residents, are knowledgeable about the public school system and can help parents connect to local resources. CHWs potentially provide the missing link to coordinate between multiple sectors to optimize interventions[36].

Asthma-trained community health workers have the potential to ensure that caregivers make and keep follow-up appointments post ED visits by conducting periodic telephone assessments. They are invariably equipped with asthma educational knowledge and resources that would be an invaluable asset to patients and their families. Not only are they trained in the nature of the disease, but they are often members of the same communities as patients and families, giving them an advantage on building bridges and establishing relationships between the family and primary care office.

4 Compliance Statement

This study will be conducted in full accordance all applicable Children's Hospital of Philadelphia Research Policies and Procedures and all applicable Federal and state laws and regulations including 45 CFR 46.

The investigators will perform the study in accordance with this protocol, will obtain consent and assent, and will report unanticipated problems involving risks to subjects or others in accordance with The Children's Hospital of Philadelphia IRB Policies and Procedures and all federal requirements. Collection, recording, and reporting of data will be accurate and will ensure the privacy, health, and welfare of research subjects during and after the study.

5 Study Objectives

6 Primary Aim

The primary aim of this study is to compare effectiveness of the primary care and school interventions to improve asthma control using main and simple effects from the factorial design.

- *Specific Aim 1a.* Compare the main effects of primary care CHW and school CHW intervention to control group.
- *Specific Aim 1b.* Compare the differences between children outcomes who receive both interventions (primary care AND school) to only one intervention (primary care OR school).
- *Specific Aim 1c.* Compare each of the active interventions groups (primary care, school, combination) to control.

7 Secondary Aims

The secondary aim of this study is to explore moderators and mechanisms of effectiveness of the interventions.

- *Specific Aim 2a.* Investigate moderators of intervention effectiveness outcomes to identify subgroups of children that may be more likely to benefit from the study interventions.
- *Specific Aim 2b.* Investigate mechanisms of the effect of the intervention, i.e. linkage of communications between school and primary care CHWs and daily participation in school-based asthma therapy, on asthma control, reduced symptom days, and decreased school absences.

8 Investigational plan

9 General Schema of Study Design

A factorial design will be used to understand the effectiveness of primary care and school CHWs in the implementation of Evidence Based Interventions (EBI) Yes We Can™ Children's Asthma Program in the primary care setting, and Open Airways For Schools® and school-based asthma therapy in the school setting. West Philadelphia children with uncontrolled asthma will be recruited in the Emergency Department or primary care clinic and randomized to a primary care CHW (P+) or usual care (P-), and identified as being in a participating school that has been randomized to a CHW-led

school intervention (S+) or none (S-). This study design demonstrates how each sector will be involved in this comprehensive implementation plan. The primary outcomes is asthma control. Secondary outcomes include symptom free days, school absences, hospital and emergency room utilization and cost savings.

This will be a randomized controlled clinical trial of children diagnosed with asthma, ages 5-13 years old randomized after consenting to primary care CHW (P+) or no primary care CHW (P). In the Primary Care Based CHW arm (P+) the CHW will provide one-on-one asthma education in the office and home, environmental trigger mitigation in the home, assistance in identifying care coordination goals with linkages to resources to address barriers and will facilitate communication between the health care provider and the family. The intervention group (P+) will have a total of four visits to the primary care office where they will receive asthma self-management education in the office, review of medications, asthma control assessment, pulmonary function assessment and care coordination. They will receive three intervention home visits where the CHW will reinforce asthma self-management skills, assess the home environment for asthma triggers, provide supplies for environmental remediation and educate caregiver regarding asthma trigger avoidance techniques. This arm will also be eligible for school based direct observed asthma treatment called school-based asthma therapy (SBAT). At twelve months the intervention group will receive an end of study assessment in the primary care office and in the home. The control group will not receive any of the aforementioned services for one year after enrollment. They will be referred to community asthma classes administered by CAPP. After one year the control group will receive one home visit at which time they will receive an environmental home assessment and environmental remediation supplies.

Children in both arms (P+ and P-) may attend an intervention school. Roughly 30 elementary schools have been identified by the School District of Philadelphia to participate in this project. Several public charter schools including three public charter schools served by school-based health centers managed by Education Plus Health have been identified to participate in this study. One half of the schools (S+) will be randomized to receive **Open Airways for Schools *Plus*** designed by Clark, et. al., an EBI asthma education and care coordination model for schools, and School Based Asthma Therapy (directly observed controller medication use).[11] The remaining half of the identified schools (S-) will be offered training on how to implement the two evidence-based programs in years 4 and 5 of the project.

10 Screening Phase

We will screen electronic health records to recruit 640 children ages 5-13 years old with uncontrolled asthma (evidenced by one or more asthma exacerbations requiring oral steroids within the past year -OR- two ED visits for asthma within the past year -OR- one hospitalization for asthma within the past year) from several primary care centers (CHOP Care Network – Cobbs Creek, CHOP Care Network - Karabots, CHOP Care Network – South Philadelphia, The Pediatric and Adolescent Medicine Centers of Philadelphia (PAMCOP) and Federally Qualified Health Centers) who live in seven West Philadelphia ZIP codes (19104, 19131, 19139, 19142, 19143, 19151, 19153). In addition, CHOP Emergency Department (ED) will screen and refer children for enrollment when they are seen in the ED. Community partners will also refer children at community events. All screening surveys will be faxed to a confidential fax line and caregivers of all referred patients will be asked if they would like to be screened for study eligibility and participation.

Participants that both meet eligibility criteria and are interested in participation, will be consented/assented using a CHOP IRB reviewed consent form prior to participation in the study. The parent/guardian and the child will have 10 minutes or greater to review the consent form and ask any questions about participation.

11 Primary Care Based Intervention

Children who are randomized to the primary care arm will be assigned a primary care community health worker (CHW). We will implement the ***Yes We Can***™ Children's Asthma Program [12] intervention which is a medical-social model based on a chronic care approach, including risk stratification, clinical care management, social care coordination by a community health worker, and primary care asthma champions. This intervention includes asthma education and trigger reduction

visits and care coordination. There will be five clinic visits and four home visits over 12 months implemented by the ***primary care CHW*** who is integrated into the practice.

Study Visit 1 (primary care office): Once consented and randomized, the CHW will collect baseline data (study questionnaires, weight, height, spirometry), administer a caregiver needs assessment and assist caregiver in choosing 2-3 care coordination goals such as keeping appointments, reduce secondhand smoke exposure or using medications correctly. The CHW will conduct in-office asthma teaching (medications/devices/asthma care plan), coordinate specialist visits and assist caregivers in voicing questions/concerns about medications or treatment plans to the primary care provider. They will personally schedule patients for follow-up visits with the primary care provider. Home Visits 1, 2 and 3: The first three home visits will occur within 2, 4 and 6 weeks after enrollment. At the first visit the CHW will conduct an environmental assessment of the living area and bedroom for common asthma triggers. Based on those findings the CHW will educate regarding asthma trigger avoidance and give supplies to reduce those triggers (mattress and pillow covers, roach bait, mice traps, tiles for carpeted floors) at the second visit. The CHW will also review medications available for the child at home and whether forms/prescriptions have been completed for school. They will coordinate with the primary care team as needed. Video conferencing through a HIPAA-compliant application licensed by CHOP, including but not limited to Vidyo telehealth visits in MyCHOP, may be used for home visits that cannot be conducted in person due to health or safety concerns. For telehealth home visits, supplies will be provided to families via U.S. postal mail with caregivers providing confirmation of receipt. Study Visits 2, 3, and 4 (primary care) will occur at 3, 6, and 9 months post-randomization. At these visits, the participants will receive asthma maintenance care by the provider/clinician and pulmonary function testing (spirometry) in addition to the education and care coordination activities by the CHW as described in the first visit. A member of the research team other than the assigned CHW (Home Visitor) will administer study questionnaires at these visits. At Home Visit 4 at 12 months post-randomization the CHW will conduct an environmental assessment to document trigger reduction techniques in the home and review medications. At Study Visit 5 at 12 months post-randomization, the asthma control questionnaire (ACQ)[38], additional study questionnaires, pulmonary function testing (spirometry) and care coordination goals will be completed. Study staff will administer a CHW satisfaction survey via telephone between 13 and 24 months post-randomization once all primary care and school interventions have been completed.

12 School Based Intervention

West Philadelphia elementary schools who have agreed to participate will be randomized to either receive the school intervention facilitated by a school CHW or usual school care (no intervention). Intervention schools will receive **Open Airways for Schools *Plus*** designed by Clark, et. al. to improve the disease management skills of children with asthma and enhance control of asthma in the school[11]. It contains four "essential components" of the recently published School-based Asthma Management Program (SAMPRO) summit guidelines and has been found to decrease both asthma symptom days and school absences[39–41]. The school intervention includes: (1) **Open Airways for Schools®** curriculum for all children with asthma. One class series will be conducted by the school CHW each semester. (2) Orientation to asthma and control strategies for principals and school personnel at the start of each school year led by the study team along with the school CHW, (3) For School District of Philadelphia public schools, walk-throughs for custodial personnel to address potential environmental asthma triggers. This will be managed and conducted by school district personnel, and (4) Assistance with obtaining asthma care plans and medication administration forms from primary care provider offices facilitated by the school CHW.

For participants assigned to both interventions, the school and primary care CHWs will collaborate to coordinate care across the school nurse and primary care offices. In addition to the primary care and school interventions above, this treatment group will receive enhanced coordination with **School-Based Asthma Therapy** for prescribed daily controller medication[42]. The primary care CHW will obtain a copy of the current asthma care plan, school medication administration form, a rescue inhaler prescription for school, and an asthma controller medication prescription to

conduct school based asthma therapy. Prescriptions for rescue inhalers and asthma controller medications will be dispensed before the child leaves the primary care office. Depending on the participant's health insurance coverage the prescriptions may be filled using an automated medication dispensing system (mobile pharmacy) at Cobbs Creek, Karabots, and South Philadelphia CHOP primary care practices or through an internal pharmacy at a Federally Qualified Health Center. Primary care CHWs will deliver current asthma care plans, medication administration forms, rescue inhalers (if applicable), and asthma controller medications (if applicable) directly to School CHWs at weekly team meetings. School CHWs will deliver these items to the school nurse to file/store in a designated location in the school nurse's office. The school nurse and/or School CHW will coordinate with teachers to schedule an intake appointment for children receiving school based asthma therapy at which the nurse will complete a metered dose inhaler (MDI) checklist with the child to assess ability to self-administer medications and to schedule a time for the child to return to the office for daily medication administration. Medication use (both rescue and controller) will be recorded by the school nurse or school CHW[42]. If nurse is not present, as per usual school protocol, the principal will be responsible for assigning this task. For this treatment group, asthma management skills will be reinforced at home, school and in the primary care office.

Families of children who are not randomized to the primary care intervention will be offered Community Asthma Prevention Program (CAPP) asthma classes, conducted at community sites, which are available to the general public. Data will be collected from the control arm at 3, 6, 9 months by phone or in person. At 12 months an office visit will be scheduled, pulmonary function testing (spirometry) performed and end-of-study data collected. After completing the study these patients will receive one home visit from a primary care CHW to complete an asthma trigger assessment and distribute asthma trigger reduction supplies.

Schools who are not randomized to intervention arm will be offered training after the study data collection period has ended and during the last year of grant funding in how to implement the school asthma interventions and will be provided with information on how to sustain these interventions by obtaining reimbursement from health insurance companies for school CHW services.

During the COVID-19 pandemic, the study activities detailed above will be modified as follows to preserve the health and safety of participants, healthcare professionals, study staff and the West Philadelphia community. Data collection for study visits may take place remotely by telephone or video conference using a HIPAA-compliant application licensed by CHOP. In person clinic visits solely for study purposes will take place as permitted by CHOP Research Institute Policy and Guidelines. Study activities designed to take place in a school setting may take place remotely by telephone or video conference using a HIPPA-compliant application licensed by CHOP.

Asthma control is the primary outcome of this study. Symptom-free days, and school absences are secondary outcomes in this study. Asthma control has been cited by the NAEPP guidelines and asthma outcomes workshop as an important goal for prevention of asthma morbidity. In several studies, poor asthma control, whether defined by the patient (using self-reported, validated instruments) or by national asthma guideline classifications, has been strongly associated with an increased risk of asthma exacerbations which may lead to emergency room visits, hospitalizations and even death[43,44]. Asthma control will be measured using Juniper's Asthma Control Questionnaire, a validated instrument for children and adults[38]. Symptom-free days will be based on parental report recall of the last two weeks, consistent with previous studies[45,46]. School absences will be measured by school and parental report. Additional secondary outcomes will include asthma-related emergency visits and hospital visits (obtained from CHOP EHR), and cost-savings. Moderator variables will be measured using the CARAT survey which is a **C**hild **A**sthma **R**isk **A**ssessment **T**ool designed to help clinicians, asthma counselors and parents determine potential risks for children with asthma[47,48].

Data will be collected for all individually randomized participants at baseline, 3, 6, 9 and 12 months by a member of the research team in person or by phone. Participants will be compensated $25 for each visit with data collection at baseline, 3, 6, and 9 months and $45 for the end of study visit at 12 months.

13 Allocation to Treatment Groups and Blinding

The overall design will randomize participating schools to the school intervention (S+) or usual care (S-) based on predefined strata (defined by size, location, public school or public charter school status, etc.) to enhance balance. When children with uncontrolled asthma and their caregivers present in the primary care offices and provide consent to participate, these patients will be randomized either to a primary care CHW (child-level intervention) (P+) or usual care (P-), and this randomization will be stratified by the child's current elementary school and clinic of recruitment to ensure balanced assignment in the factorial design.

14 Study Duration, Enrollment and Number of Sites

15 Duration of Study Participation

The study duration per subject will be up to 24 months, if participant has not completed the assigned study interventions within the first 12 months of participation.

16 Total Number of Study Sites/Total Number of Subjects Projected

The study will be conducted at approximately six healthcare sites, including CHOP primary care sites (Cobbs Creek, Karabots and South Philadelphia), CHOP ED and approximately two Federally Qualified Health Centers serving West Philadelphia, and no greater than 40 elementary schools according to the number of schools agreeing to participate. This includes as many as 30 public schools and as many as ten public charter schools located in the West Philadelphia area.

Recruitment will stop when 640 subjects are enrolled. It is expected that at least 600 children will be evaluable subjects.

17 Study Population.

18 Inclusion Criteria

1) Parents (guardians) and their children ages 5-13 years

   - Children will include those who:

     a) Have a diagnosis of asthma
     b) Have uncontrolled Asthma (as evidenced by one or more asthma exacerbations requiring oral steroids within the past year -OR- 2 ED visits for asthma within the past year -OR- 1 inpatient admission for asthma within the past year).
     c) Reside in one of seven West Philadelphia zip codes:(19104, 19131, 19139, 19142, 19143, 19151, 19153)
     d) Are enrolled in elementary school
     e) Are patients at primary care provider practices at CHOP CN Cobbs Creek, CHOP CN Karabots, CHOP CN South Philadelphia, PAMCOP or a Federally Qualified Health Center serving West Philadelphia residents.

2) Parental/guardian permission (informed consent) and if appropriate, child assent.

3) English Speaker

19 Exclusion Criteria

1) Subjects with other chronic respiratory illnesses such as cystic fibrosis

2) Cyanotic congenital heart disease

3) MRCP-Mental retardation and/or cerebral palsy

4) Severe Neurological Disorder

5) Cyanotic congenital heart disease

6) Parents/guardians or subjects who, in the opinion of the Investigator, may be non-compliant with study schedules or procedures.

7) Those who do not live in West Philadelphia (zip codes: 19104, 19131, 19139, 19142, 19143, 19151, 19153)

Subjects that do not meet all of the enrollment criteria may not be enrolled. Any violations of these criteria must be reported in accordance with IRB Policies and Procedures.

20 Study Procedures

21 Screening Visit

- Medical Record Review of the child
- Verbal consent/complete eligibility screening
- Identify child's elementary school
- Schedule first visit

22 Study Treatment Phase

23 Study Visit 1 (Enrollment)

All study participants will complete the following:

- Consent/Assent
- Baseline Questionnaire
- Clinical and vital sign measurements of child [weight, height, BMI, blood pressure, pulse, temperature, pulse oximetry (SpO2)]
- Pulmonary function test (spirometry) with child if not obtained from medical record
- 2-week parental recall of symptoms, school absences, and work days missed
- Asthma Control Questionnaire
- Child Asthma Risk Assessment Tool (CARAT)
- Caregiver's Quality of Life Questionnaire
- Caregiver Needs Assessment
- Cost Analysis Parent Questionnaire
- Observe MDI technique and complete MDI Checklist

- Assess possible adverse events from date of enrollment

24 Study Visits 2, 3, and 4 (Primary Care Office Visits)

Primary Care office visits will be scheduled for 3, 6 and 9 months post study enrollment. All study participants will complete the following:

- Asthma Control Questionnaire
- Clinical and vital sign measurements of child [weight, height, BMI, blood pressure, pulse, temperature, pulse oximetry (SpO2)]
- Pulmonary function test (spirometry) with child if not obtained from medical record.
- 2-week parental recall of symptoms, school absences, and work days missed
- Cost Analysis Parent Questionnaire
- Assess possible adverse events
- Review child's medical record for utilization of healthcare services
- Review care coordination goals and direct to community resources

25 Study Visit 5 (12-Month Follow-Up)

All study participants will complete the following:

- Pulmonary function test (spirometry) with child if not obtained from medical record.
- Clinical and vital sign measurements of child [weight, height, BMI, blood pressure, pulse, temperature, pulse oximetry (SpO2)]
- 2-week parental recall of symptoms, school absences, and work days missed
- Asthma Control Questionnaire
- Caregiver's Quality of Life Questionnaire
- Cost Analysis Parent Questionnaire
- Child Asthma Risk Assessment Tool (CARAT)
- Administer Asthma Knowledge Quiz
- Observe MDI technique and complete MDI Checklist

26 Primary Care Based Intervention:

27 Study Visit 1 (Baseline)

Additional components of Visit 1 will include:

- Care coordination goals developed with the parent/caregiver

- Coordinate school forms, medications and devices

28 Home Visit 1

Initial home visit scheduled within 2 weeks of enrollment. Primary care CHW will do the following with the child and parent/caregiver:

- Administer home environmental assessment survey
- Take photographs of child's bedroom and home's main living area (environmental remediation assessment)
- Educate about asthma basics and asthma trigger avoidance
- Review asthma medications and asthma care plan
- Administer Asthma Knowledge Quiz
- Observe MDI technique with either the child or the parent/caregiver
- Give asthma supplies tailored to environment and show caregiver how to use
- Assess possible adverse events
- Review child's medical record for utilization of healthcare services
- Establish care coordination goals and refer to community resources as needed

29 Home Visit 2

Second home visit is scheduled within 4 weeks of enrollment. Primary care CHW will do the following with the child and caregiver/parent:

- Complete follow-up home environmental assessment
- Educate about types of asthma medications, how they work and how to use them properly
- Review asthma medications and asthma care plan
- Give asthma supplies tailored to environment and show caregiver how to use
- Observe MDI technique with the child or the parent/caregiver
- Assess possible adverse events
- Review child's medical record for utilization of healthcare services
- Review care coordination goals and refer to community resources as needed

30 Home Visit 3

Third home visit is scheduled within 6 weeks of enrollment. Primary care CHW will do the following with the child and parent/caregiver:

- Complete follow-up home environmental assessment
- Take photographs of child's bedroom and home's main living area (environmental remediation assessment)
- Educate about communicating with teachers and family members about asthma
- Review asthma medications and asthma care plan
- Observe MDI technique with the child or the parent/caregiver
- Administer Asthma Knowledge Quiz
- Assess possible adverse events
- Review child's medical record for utilization of healthcare services
- Review care coordination goals and refer to community resources as needed

31 Study Visits 2, 3, and 4 (Primary Care Office Visits)

Additional components of Study Visits 2, 3, and 4 will include:

- Coordinate school forms, medications and devices
- Teach asthma care plan
- Review care coordination goals and refer to community resources as needed

32 Home Visit 4 (12-Month Follow-Up)

- Follow-up environmental assessment
- Take photographs of child's bedroom and home's main living area (environmental remediation assessment)
- Administer Asthma Knowledge Quiz

In between visits:

- Primary care CHW will ensure that caregiver has all forms needed for rescue medication administration at school. Participants attending an intervention school may have rescue and controller medications hand delivered to school nurse through transfer from primary care CHW to school CHW at weekly meetings.

33 Study Visit 6 (Post 12-Month Follow-Up)

- Administer CHW Caregiver Satisfaction Survey

- Administer Asthma Control Questionnaire
- 2-week parental recall of symptoms, school absences, and work days missed

34 Unscheduled Visits

Usual care includes the caregivers' ability to make clinical visits for their children whenever they feel that it is necessary. In addition, caregivers may contact the CHW for an unscheduled visit via cell phone for non-clinical issues to assist them in meeting their goals. CHW will assess goals on a case by case basis and make follow-up calls as needed. If the CHW is requested to accompany caregivers for unscheduled visits, the CHW will do their best to accommodate the caregivers' needs. CHWs are expected to contact families within 48 hours after receiving phone messages from families or patients to assess whether a home visit or a primary care office follow-up appointment is needed.

35 School Intervention

36 Community Health Worker Coordination

The primary care CHW will contact the school CHW to identify newly consented children present in each West Philadelphia elementary school participating in the study during weekly team meetings. The primary care CHW will then be responsible for contacting the child's caregiver to arrange a visit for collection of appropriate forms and medications.

37 School Nurse Coordination

School CHW will:

- Provide a portable locked filing cabinet that can be stored in the school nurse's office for study participants
- Help school nurse with filing medication administration forms (med1 forms) and attaining asthma medications for study participants.

38 Classroom Environmental Assessments

School CHW will:

- Visit participant classrooms to do an environmental assessment for asthma triggers within the first 2 weeks of enrollment in the study.
- Using environmental assessment findings, advise teacher of appropriate asthma trigger avoidance measures.
- Provide teacher with supplies to assist in decreasing asthma triggers in the classroom. Supplies may include white vinegar and a spray bottle for cleaning, electrostatic dust cloths (dusters), and storage bins. School CHW will notify appropriate school personnel of any pest problems identified in the classroom.

39 Open Airways for Schools Curriculum

Implementation of the Open Airways for Schools Curriculum will occur over three to six asthma management education sessions.

- The school CHW will conduct Open Airways for Schools classes at least once every semester for children with asthma facilitated by the school nurse or designated school staff by the principal.
- Classes with be separated into sessions by grade.

40 School Based Asthma Therapy

School Based Asthma Therapy (SBAT) involves the administration of controller or preventive asthma medication at school in addition to rescue asthma medication as needed. School CHW will coordinate with teachers/school administers for all children that attend a school with the asthma intervention and have a primary CHW. To do this, school CHW will:

- Schedule an initial intake for children receiving SBAT
- Utilize an MDI checklist for school nurse or school CHW to assess child's ability to self-administer his/her medication
- Schedule a time for the child to return to the school nurse's office for daily medication administration.
- Utilize a tracking system for the child's medication use (rescue and controller)

41 Rescue Medication Administration

Participants may use asthma rescue medication if there is an exacerbation in his/her asthma symptoms in the school, home and clinic as a part of their usual care.

42 Subject Completion/Withdrawal

Subjects may withdraw from the study at any time without prejudice to their care. They may also be discontinued from the study at the discretion of the Investigator for lack of adherence to study procedures. It will be documented whether or not each subject completes the clinical study. If the Investigator becomes aware of any serious, related adverse events after the subject completes or withdraws from the study, they will be recorded in the source documents and on the CRF.

Subjects who become a ward of the state during study participation will continue to have their medical records reviewed, but will no longer participate in study visits or the completion of questionnaires. The state agency assigned legal guardianship of the participant will be asked to provide consent for medical record review of the participant during the study follow-up period. If consent cannot be obtained the participant will be withdrawn.

In the event that a parent or legal guardian regains custody and a participant is no longer a ward of the state, the legal guardian may be approached to provide consent for study participation. Once consent has been obtained, study visits can resume for the duration of the follow-up period.

43 Early Termination Study Visit

If participants withdraw prior to completing the study, all previously collected data will be stored and used for analysis.

44 Study Evaluations and Measurements

45 Individual Participant Measures

- Children will be measured for **height and weight** (using standard scales and stadiometers), **blood pressure**, **pulse**, **temperature**, and **pulse oximetry** (SpO2) at baseline, 3, 6, 9, and 12 months following enrollment. This information may also be abstracted from the medical record.
- **Pulmonary function tests (spirometry)** will be abstracted from the electronic health record if possible. If spirometry is not completed by the child's healthcare team as a part of usual clinical care, it will be completed by a trained community health worker at baseline, 3, 6, 9 and 12 months post-enrollment. Each recording will be printed out to become a part of the child's medical record. Pulmonary function testing completed for clinical purposes within one month before or after a baseline or follow-up study visit will be abstracted from the medical record if testing is unable to be completed at the time of a study visit.
- Juniper's **Asthma Control Questionnaire** is a validated survey and will be administered by a member of the research team to the parent/caregiver by reading out loud to avoid any literacy barriers. This will be administered at baseline, 3 months, 6 months, 9 months and 12 months
- A home **environmental assessment** will be conducted by a CHW in the living room and child sleeping area to note common triggers such as dust, pests, pets, mold and tobacco smoke measured at Home Visits 1, 2, 3, and 4.
- CAPP's **asthma knowledge survey** will be used to assess knowledge at home visits before, during and after the primary care intervention. This survey may be administered out loud to the parent/caregiver to avoid any literacy barriers.

- CHW will ask parent/caregiver for a **2-week recall of daytime cough or wheeze, nighttime cough or wheeze, missed school days, missed parent workdays at the time of the visit.** This will be recorded on a tracking form in REDCap.
- The **Caregiver Needs Assessment** will be administered to the parent/caregiver at the baseline visit. The needs assessment is a validated instrument that focuses on the caregiver's knowledge about asthma avoidance measures and their comfort level with managing their child's asthma. For participants receiving the primary care intervention, this tool will assist the primary care community health worker with tailoring the educational component of the program to fit the needs of the individual family.
- **Adverse Event Forms** will be completed as needed throughout study participation.

46 Group Level Primary care and School Measures

- A REDCap tracking form will be used for documentation of time, date and dose of medication dispensed in **School Based Asthma Therapy**. This information will be collected and recorded by the school CHW.
- A **classroom environmental assessment** form will be completed by the school CHW at baseline and at end of semester for study participant classrooms.
- As a part of the Open Airways for Schools Curriculum, an **Open Airways Quiz** will be administered at the beginning and end of a school asthma management class series.
- Fidelity to the school EBI's will be monitored via observation by study research staff utilizing yes/no completion checklists of core components of each intervention. These checklists include the **MDI checklist**, **OAS lesson plan** and **classroom environmental survey** once each semester. It is expected that **90%** of the core components will be implemented for maintenance of fidelity to the intervention.

47 Screening and Monitoring Evaluations and Measurements

48 Medical Record Review

The following variables will be abstracted from the medical chart.

- Date of birth
- Weight
- Height
- Blood pressure
- Pulse
- Temperature
- SpO2 (Pulse Oximetry)
- Zip code of residence
- Gender
- Race/Ethnicity
- BMI
- Pulmonary function test results

- Asthma-related healthcare utilization (hospital, emergency, urgent care visits)

49 Physical Examination

No physical exam outside of usual care will be conducted in this study.

50 Vital Signs

Weight will be measured with a standardized scale.

Height will be measured with a standardized stadiometer.

Blood pressure, pulse, temperature and SpO2 will be measured by clinic staff using standard clinical procedures and equipment. This information will be abstracted from the medical record by study staff.

Pulmonary function will be measured using a Morgan Scientific spirometer.

51 Efficacy Evaluations

Primary Outcome:

1. Asthma Control

Secondary Outcomes:

1. Asthma Symptom Days
2. School Attendance
3. Utilization of ED/Hospital for Asthma

52 Diagnostic Tests, Scales, Measures, etc.

Primary Outcome:

- **Asthma control** will be measured with Juniper's Asthma Control Questionnaire at baseline, 3 month, 6 month, 9 month and 12 month study visits. We will assess for changes in the ACQ scores from enrollment to completion of the study.

Secondary Outcomes:

- **Asthma Symptoms** will be measured using a 2 week recall diary at the baseline, 3 month, 6 month, 9 month and 12 month study visits.
- **School Absences** will be measured by school and parental report
- **ED visits and hospitalizations** for an asthma-related cause (ICD-9 493.XX) in the 12 months preceding the start of the study and the 12 months subsequent to study initiation, abstracted from the Epic medical record. We will assess for changes in ED and hospitalization usage following study enrollment.

53 Safety Evaluation

Adverse event reports will be collected at each visit. These reports will be reviewed at weekly meetings by the PI to determine if they are a result of the study.

The Funding agency will assemble a DSMB to oversee safety of the interventions.

54 STATISTICAL CONSIDERATIONS

55 Primary Endpoint

The primary endpoint is the difference in asthma control between the intervention and the control group.

56 Secondary Endpoints

Secondary endpoints will include the following:

- The change in asthma symptom days between the intervention and the control group
- The change in school absences between the intervention and the control group
- The change in ED visits and inpatient hospitalizations for an asthma-related cause between the intervention and the control group.

57 Statistical Methods

58 Baseline Data

Baseline and demographic characteristics will be summarized by standard descriptive summaries (e.g. means and standard deviations for continuous variables such as age and percentages for categorical variables such as gender).

59 Efficacy Analysis

The primary analysis will be based on an intention to treat approach and will include all subjects randomized at Study Visit 1. The primary efficacy endpoint will be the change in asthma control between Study Visit 1 and Study Visit 5. Secondary efficacy endpoints will include the change in asthma symptom days and school absences between Study Visit 1 and Study Visit 5.

60 Subgroup Analysis: Mediator and Moderator Variables

Baseline variables including asthma control, BMI, school type (public/charter) and demographic characteristics which may serve as moderators of intervention effectiveness will be evaluated through subgroup analyses. These variables will be used to identify subgroups of children that may be more likely to benefit from the study interventions. Mediator variables such as communication between school and primary care and level of participation in school-based asthma therapy will be evaluated to better understand the mechanism of effect of the intervention on the study's primary outcomes.

61 Sample Size and Power

The factorial design has enhanced statistical power and can handle cluster randomization of schools. Our statistical methods will also estimate variation in the effect across schools over time. First, linear mixed effects models with random intercepts and slopes for school, and fixed effects for the school-level intervention, time, and time-by-intervention interaction (the estimate of interest) will be applied. Likewise, the main effect for the child-level intervention and a time*intervention interaction term will be included in the model, and variation across multiple measurements on a child within a school will be accounted for by child-level random effects. Second, marginal models using generalized estimating equations will produce robust estimates that adjust for clustering at the school level. Third, assumption-free, randomization-test-based (permutation test) methods do not rely on assumptions of parametric models. We will use conventional levels of statistical significance ($p=0.05$) for all pre-specified comparisons for these aims and shall report all results to confirm consistency of findings and their robustness to model specifications. Variability of the intervention effect across schools will reflect consistency of intervention effects and thus generalizability in new settings. Both mixed effects models and permutation-test methods will estimate variance components. All contrasts noted in specific Aims 1a through 1d are handled with the same modeling specifications. This approach will also be used to account for change over time across schools, and to assess sustainability of the proposed interventions.

Missing data on covariates will be handled using formal multiple imputation as needed. The proposed mixed effects models make limited assumptions of "missing at random" (MAR). To the extent that children are lost to follow-up, we shall include in our models covariates that are associated with the probability of dropout to limit confounding by dropout. We anticipate no school withdrawals, but in case of loss, we will use all data collected until time of dropout. The primary analysis will be "as randomized" (also called intention to treat). Reporting will follow the CONSORT guidelines for randomized studies and cluster randomized designs.

All power estimates are based on custom programmed simulations. The pre-specified contrasts from specific aims 1a, 1b, and 1c, appear in Table 2. All power estimates represent changes over time in the primary outcome (asthma control) in units of standard deviation of change. For translation into clinical terms, the minimally important difference (MID) for children on the Juniper's Asthma Control Questionnaire is 0.5 standard deviation units, which equates to 0.4 units on the questionnaire scale (for children ages 6 to 17 years). Estimates assumed a modest degree of variation in outcomes across schools, which equates to clustering of children within schools. For an overall effect size of 0.5 standard deviation units, we assumed that individual schools would vary from no effect (0.0) to a large effect (1.0 sd units). This degree of variation corresponds to a random cluster (school) effect of 0.025 sd units, and an intraclass correlation coefficient (ICC) of 0.06. With an average of 28 children per school, this variability equates to a design effect (variance inflation factor) of about 2.5. As the table shows, the contrasts for the primary care intervention are powerful to show even small effects, because they are within-cluster comparisons and therefore are influenced if at all only slightly by clustering of children within schools. Across school contrasts, of the effects of the school intervention, remain powerful for MID changes.

Table 2. Power for Contrasts of Interest

Specific Aim	Contrast	Contrast (Table cell)	Stipulated Effect size(sd)			Estimated Power
			P	S	P,S	
1a	S+ v S-	a,b vs c,d	0.5	0.5	0.1	0.90
1a	P+ v P-	a,c vs b,d	0.25	0.25	0.1	0.95
1b<br>1b	S+P+ v S-P+<br>S+P+ v S+P-	a vs c<br>a vs b	0.5<br>0.35	0.5<br>0.5	0.1<br>0.0	0.95<br>0.88
1b	S- P+ v S+P-	b vs c	0.75	0.25	0.0	0.92
1c	S+P+ v S-P-	a vs d	0.25	0.25	0.1	0.96
1c	S- P+ v S-P-	c vs d	0.35	0.5	0.0	0.90
1c	S+P- v S-P-	b vs d	0.5	0.5	0.1	0.90
20%	Subgroup					
2a	S+ v S-	a,b, vs c,d	0.5	0.6	0.1	0.83
2a	P+ v P-	a,c vs b,d	0.5	0.6	0.1	0.88

P=Primary care effect; S=School Effect; PS= interaction; "+" = , "-" = control

Cells e, f are not included in this table for simplicity and to be conservative.

62 Interim Analysis

Interim efficacy or safety analyses not expected.

63 Study Medication (Study DEVICE or OTher Study INTERVENTION)

64 Description

All medication taken by participating children will be prescribed by their primary care provider.

65 SAFETY MANAGEMENT

66 Clinical Adverse Events

Clinical adverse events (AEs) will be monitored throughout the study.

67 Adverse Event Reporting

Since the study procedures are not greater than minimal risk, SAEs are not expected. If any unanticipated problems related to the research involving risks to subjects or others happen during the course of this study (including SAEs) they will be reported to the IRB in accordance with CHOP IRB SOP 408: Unanticipated Problems Involving Risks to Subjects. AEs that are not serious but that are notable and could involve risks to subjects will be summarized in narrative or other format and submitted to the IRB at the time of continuing review.

68 STUDY ADMINISTRATION

69 Treatment Assignment Methods

70 Randomization

Allocation concealment will be preserved by randomly permuted block. This randomization will produce a 2-by-2 factorial design, partly clustered (by school).

TABLE 3

		Child-Level Intervention	
		P+	P-
School Level Intervention	S+	A	B
	S-	C	D
	none	E	F

The proposed design results in 6 groups of children. Children who do not attend a participating school will be randomized to the primary care CHW (child-level) intervention or not (groups E and F) and will be followed for the duration of the study. The cells A,B,C will permit comparison of any of the 3 combinations of school and child intervention against the group that has neither (D), and will permit marginal analysis of the effect of the school intervention (A+B vs C+D), and the marginal effect of the primary care intervention (A+C+E vs B+D+F). We will collect baseline data on children and then follow schools and children over time. This design avoids contamination and interference through cluster randomization, but benefits from added power of a longitudinal analysis. It permits estimation (and testing) of interaction between the school and individual interventions. All randomization schemes will be prepared by the project statistical team and implemented using opaque sealed envelopes or a similar process electronically via REDCap.

71 Blinding

The PI will be blinded to the intervention/treatment group to the extent possible.

72 Unblinding

Unblinding will occur if there is an adverse event that warrants investigation or if the DSMB instructs the PI to become unblinded.

73 Data Collection and Management

For the study intervention, data will be collected, accessed and stored by the CHWs and study research team. Any paper forms will be stored in a locked cabinets in the Community Asthma Prevention Program's (CAPP) badge-only accessible suite. Data collected using REDCap is accessible only by the CHWs and research study staff. Designated school administrators (i.e. principal), the school nurse and the child's primary care provider will only have access to participant health care information necessary to conduct asthma management in the schools and primary care office, but none of the research data collected by the CHWs and study staff. Information will be kept confidential under HIPPA guidelines and Institutional policies.

We will implement the following:

- A master list containing PHI and subject ID number separate from data forms (paper and electronic) that have only a study ID number.
- The master list and any other files with be password protected and kept on a secure CHOP computer to prevent double enrollments.

CHOP's Department of Biomedical and Health Informatics (DBHi) will work with the research team to extract variables from the EHR, and also provide support for the design and use of the REDCap database.

74 Confidentiality

All data and records generated during this study will be kept confidential in accordance with Institutional policies and HIPAA on subject privacy. The Investigator and other site personnel will not use such data and records for any purpose other than conducting the study. Hard data will be kept in locked file cabinets in the CAPP suite, and will be destroyed 10 years after the end of the study unless subjects consent to contact regarding future research. Any soft data or electronic data will be password protected and kept on a secure CHOP computer.

75 Regulatory and Ethical Considerations

76 Data and Safety Monitoring Plan

The study sponsor will appoint members to a Data and Safety Monitoring Board. Reporting on study conduct, safety measures, data collection and analysis will be provided to the DSMB.

77 Risk Assessment

Potential risks directly related to the study are minimal. Children may have difficulty with pulmonary function testing, which may be included as a part of their usual care depending on the child's healthcare team, or collected by a trained CHW. Risks indirectly related to the study, particularly in a group of children where no evidence-based interventions are being conducted may include challenges with asthma management which may lead to ED visits or hospitalizations.

Another risk is the potential breach of confidentiality for participant data. When possible, data will be coded by study personnel through the use of participant identification numbers. Any screening referrals from the community will be faxed to a confidential fax line. In addition, participants could potentially feel discomfort with engaging in any of interventions, as questions may be posed requiring personal information.

78 Potential Benefits of Trial Participation

Direct benefits: Community Health Workers providing the connection between the home, school and primary care practice may reduce the burden on caregivers and facilitate asthma management for school-aged children. Additionally, as community residents, CHWs are knowledgeable about the public school system and can help parents connect to local resources. Participants will also benefit from asthma management education.

Indirect benefits: This project utilizes a pragmatic, integrated and sustainable design with evidence-based interventions to reduce asthma disparities in poor, minority children. We have engaged stakeholders with experience in the community, school district, health plans and health care systems to implement community and evidence-based interventions. These interventions, if proven successful, will reduce the disproportionate impact of asthma on minority children through the number of missed school days, ED visits and hospitalizations due to asthma exacerbations and also create a sustainable care-coordination model that can be replicated in other communities.

79 Risk-Benefit Assessment

There are minimal risks due to the possibility of loss of confidentiality and possible discomfort from answering questions. There are significant benefits to be gained through participation in the primary care CHW program. We will, to the best of our capabilities, ensure minimal risks by 1) assigning study identification numbers to patient records and for data collection purposes. Only these numbers will be shared with third parties. 2) The caregiver will always have the option of refusing to answer or participate in any component of the study without change to usual care.

80 Recruitment Strategy

We will screen electronic health records to recruit 640 parent-child dyads with children ages 5-13 years old with uncontrolled asthma (evidenced by one or more asthma exacerbations requiring oral steroids -OR- one hospitalization within the past year -OR- two ED visits within the past year) from up to five primary care centers (CHOP CN – Cobbs Creek, CHOP CN - Karabots, CHOP CN – South Philadelphia, The Pediatric and Adolescent Medicine Centers of Philadelphia (PAMCOP) and Federally Qualified Health Centers) who serve families who live in the seven West Philadelphia ZIP codes (19104, 19131, 19139, 19142, 19143, 19151, 19153). In addition, CHOP Emergency Department (ED) will screen electronic health records and refer children for enrollment at the primary care sites when they are seen in the ED. The majority of eligible participants will be identified through medical record review and documented on the screening form completed by the CHW or research assistant in the primary care centers or CHOP ED. A waiver of consent will be used to facilitate this process. If additional information is needed to determine eligibility, the CHW will approach families to see if they are interested in being screened for any missing information on the screening form. The screening form will be reviewed by study staff (SC and PI) to confirm study eligibility.

We will also post flyers in our CHOP primary care centers, CHOP ED, and also in partnering community centers. These flyers will contain the CAPP main phone number and email address for families interested in participating in the study. Community partners can also refer children at community events for study screening by research staff.

The Recruitment Enhancement Core (REC) at CHOP provides assistance with recruitment plan development and may assist us in identifying and contacting potential participants using the Clinical Reporting Unit (CRU), the CHOP Recruitment Registry, social media and internal communication resources. The REC also engages community partners and facilitates outreach on behalf of the research Institute and CHOP research studies.

81 Informed Consent/Assent and HIPAA Authorization

For the screening process, a waiver of consent documentation and HIPAA will be used. For children and parents/caregivers interested in the study, they will be given explicit information regarding the purpose and procedures of the program, the family will then be asked if they have any questions. The family will be given a consent form that will be read to them by a member of the study team describing the study procedures, the purpose of the study, that their

consent to participate in the study is strictly voluntary, and that should they consent, they have the right to end study procedures at any time. Parents who agree to participate will sign an IRB-approved combined informed consent form and HIPAA authorization on paper using a handwritten signature or on a tablet device using an electronic signature. Consent discussions may be held remotely by phone or through videoconference using a HIPAA-compliant application licensed by CHOP including but not limited to BlueJeans and Skype. For consent discussions held remotely, through video conferencing or by telephone, parents will be provided with a REDCap survey link to the informed consent and HIPAA authorization to review and sign electronically. Alternatively, parents with limited internet access can provide verbal consent for participation. Electronic informed consent forms will be stored and managed in the study's REDCap database. Child assent will be handled in the same fashion. All participants will receive a copy of the signed informed consent form and HIPAA authorization. The electronic consent form will include the patient and caregiver's typed names and written signatures using a stylus pen. Participants who provide an electronic signature will receive a copy of the signed document by email or a printed copy will be provided at the conclusion of the consent meeting. Participants providing verbal consent will receive a copy of the informed consent and HIPAA authorization and documentation of verbal consent via U.S. postal mail.

82 Payment to Subjects/Families

83 Payments to subject for time, effort and inconvenience (i.e. compensation)

Data will be collected for all individually randomized participants at baseline, 3, 6, 9 and 12 months by the research assistant or primary care CHW in person or by phone. Participants will be compensated $25 for each visit with data collection at baseline, 3, 6, and 9 months and $45 for the end of study visit at 12 months.

84 Gifts

During the study, birthday and holiday cards will be sent by mail to enrolled participants. Annually, a small holiday gift such as a jump rope, sports ball or a similar item valued at no more than $10 may be provided to each enrolled child.

85 PUBLICATION

Data may be used for scientific papers and presentations at medical conferences. All data is reported in aggregate form and individuals are never identified.

86 References

1. Akinbami LJ, Moorman JE, Bailey C, et al. Trends in asthma prevalence, health care use, and mortality in the United States, 2001-2010. *NCHS Data Brief*. 2012;(94):1-8. http://www.ncbi.nlm.nih.gov/pubmed/22617340.
2. Apter AJ, Garcia LA, Boyd RC, Wang X, Bogen DK, Ten Have T. Exposure to community violence is associated with asthma hospitalizations and emergency department visits. *J Allergy Clin Immunol*. 2010;126(3):552-557. doi:10.1016/j.jaci.2010.07.014.
3. Gold DR, Wright R. Population disparities in asthma. *Annu Rev Public Health*. 2005;26:89-113. doi:10.1146/annurev.publhealth.26.021304.144528.
4. Wright RJ. Health effects of socially toxic neighborhoods: the violence and urban asthma paradigm. *Clin Chest Med*. 2006;27(3):413-21, v. doi:10.1016/j.ccm.2006.04.003.
5. Meng Y, Babey S, Wolstein J. Asthma-Related School Absenteeism and School Concentration of Low-Income Students in California. *Prev Chronic Dis*. 2012;9. doi:10.5888/pcd9.110312.
6. Canino G, Vila D, Normand S-LT, et al. Reducing asthma health disparities in poor Puerto Rican children: the effectiveness of a culturally tailored family intervention. *J Allergy Clin Immunol*. 2008;121(3):665-670. doi:10.1016/j.jaci.2007.10.022.

7. Krieger JW, Takaro TK, Song L, Weaver M. The Seattle-King County Healthy Homes Project: a randomized, controlled trial of a community health worker intervention to decrease exposure to indoor asthma triggers. *Am J Public Health*. 2005;95(4):652-659. doi:10.2105/AJPH.2004.042994.
8. Primomo J, Johnston S, DiBiase F, Nodolf J, Noren L. Evaluation of a community-based outreach worker program for children with asthma. *Public Health Nurs*. 23(3):234-241. doi:10.1111/j.1525-1446.2006.230306.x.
9. Bryant-Stephens T, Kurian C, Guo R, Zhao H. Impact of a household environmental intervention delivered by lay health workers on asthma symptom control in urban, disadvantaged children with asthma. *Am J Public Health*. 2009;99 Suppl 3:S657-65. doi:10.2105/AJPH.2009.165423.
10. Bryant-Stephens T, Li Y. Outcomes of a home-based environmental remediation for urban children with asthma. *J Natl Med Assoc*. 2008;100(3):306-316.
11. Clark NM, Brown R, Joseph CLM, Anderson EW, Liu M, Valerio MA. Effects of a comprehensive school-based asthma program on symptoms, parent management, grades, and absenteeism. *Chest*. 2004;125(5):1674-1679. doi:10.1378/chest.125.5.1674.
12. Graham K, Ellinger M , Miller J, Tsai C, Graham S, Stout J RL. Managing Children's Asthma, A Community-Focused, Team Approach Vol. 1 and 2. 2003. http://www.cdc.gov/asthma/interventions/yes_we_can_toolkit.htm.
13. JS H, PG S, SG F, et al. Randomized controlled trial to improve care for urban children with asthma: results of the school-based asthma therapy trial. *Arch Pediatr Adolesc Med*. 2011;165(3):262-268. doi:10.1001/archpediatrics.2011.1.
14. Strunk RC, Ford JG, Taggart V. Reducing disparities in asthma care: priorities for research--National Heart, Lung, and Blood Institute workshop report. *J Allergy Clin Immunol*. 2002;109(2):229-237. doi:10.1067/mai.2002.120950.
15. PHMC. *Public Health Management Corporation's 2014-2015 Southeastern Pennsylvania Household Health Survey*.; 2015.
16. Bryant-Stephens T, West C, Dirl C, Banks T, Briggs V, Rosenthal M. Asthma prevalence in Philadelphia: description of two community-based methodologies to assess asthma prevalence in an inner-city population. *J Asthma*. 2012;49(6):581-585. doi:10.3109/02770903.2012.690476.
17. CHOP. Children's Hospital of Philadelphia: internal data. 2016.
18. Powell BJ, Waltz TJ, Chinman MJ, et al. A refined compilation of implementation strategies: results from the Expert Recommendations for Implementing Change (ERIC) project. *Implement Sci*. 2015;10:21. doi:10.1186/s13012-015-0209-1.
19. Bartholomew LK, Parcel GS, Kok G. Intervention Mapping: A Process for Developing Theory and Evidence-Based Health Education Programs. *Heal Educ Behav*. 1998;25(5):545-563. doi:10.1177/109019819802500502.
20. Schaafsma D, Stoffelen JMT, Kok G, Curfs LMG. Exploring the development of existing sex education programmes for people with intellectual disabilities: an intervention mapping approach. *J Appl Res Intellect Disabil*. 2013;26(2):157-166. doi:10.1111/jar.12017.
21. Belansky ES, Cutforth N, Chavez R, Crane LA, Waters E, Marshall JA. Adapted intervention mapping: a strategic planning process for increasing physical activity and healthy eating opportunities in schools via environment and policy change. *J Sch Health*. 2013;83(3):194-205. doi:10.1111/josh.12015.
22. Fernández ME, Gonzales A, Tortolero-Luna G, Partida S, Bartholomew LK. Using intervention mapping to develop a breast and cervical cancer screening program for Hispanic farmworkers: Cultivando La Salud. *Health Promot Pract*. 2005;6(4):394-404. doi:10.1177/1524839905278810.
23. Katzen A, Morgan M. Affordable Care Act Opportunities for Community Health Workers. 2014:25. http://www.chlpi.org/wp-content/uploads/2013/12/ACA-Opportunities-for-CHWsFINAL-8-12.pdf.
24. Allen CG, Escoffery C, Satsangi A, Brownstein JN. Strategies to Improve the Integration of Community Health Workers Into Health Care Teams: "A Little Fish in a Big Pond". *Prev Chronic Dis*. 2015;12:E154. doi:10.5888/pcd12.150199.

25. Bender BG. Overcoming barriers to nonadherence in asthma treatment. *J Allergy Clin Immunol*. 2002;109(6 Suppl):S554-9. http://www.ncbi.nlm.nih.gov/pubmed/12063512. Accessed November 4, 2016.
26. Blaiss MS, Kaliner MA, Baena-Cagnani CE, Dahl R, Valovirta EJ, Canonica GW. Barriers to asthma treatment in the United States: results from the global asthma physician and patient survey. *World Allergy Organ J*. 2009;2(12):303-313. doi:10.1097/WOX.0b013e3181c81ea4.
27. Boudreaux ED, Emond SD, Clark S, Camargo CA. Acute asthma among adults presenting to the emergency department: the role of race/ethnicity and socioeconomic status. *Chest*. 2003;124(3):803-812. http://www.ncbi.nlm.nih.gov/pubmed/12970001. Accessed November 4, 2016.
28. Simon MA, Samaras AT, Nonzee NJ, et al. Patient Navigators: Agents of Creating Community-Nested Patient-Centered Medical Homes for Cancer Care. *Clin Med insights Women's Heal*. 2016;9:27-33. doi:10.4137/CMWH.S39136.
29. Auger KA, Kahn RS, Davis MM, Beck AF, Simmons JM. Medical home quality and readmission risk for children hospitalized with asthma exacerbations. *Pediatrics*. 2013;131(1):64-70. doi:10.1542/peds.2012-1055.
30. Ayala GX, Vaz L, Earp JA, Elder JP, Cherrington A. Outcome effectiveness of the lay health advisor model among Latinos in the United States: an examination by role. *Health Educ Res*. 2010;25(5):815-840. doi:10.1093/her/cyq035.
31. Wang ML, Gallivan L, Lemon SC, et al. Navigating to health: Evaluation of a community health center patient navigation program. *Prev Med Reports*. 2015;2:664-668. doi:10.1016/j.pmedr.2015.08.002.
32. Peretz PJ, Matiz LA, Findley S, Lizardo M, Evans D, McCord M. Community health workers as drivers of a successful community-based disease management initiative. *Am J Public Health*. 2012;102(8):1443-1446. doi:10.2105/AJPH.2011.300585.
33. Berenson RA, Hammons T, Gans DN, et al. A house is not a home: keeping patients at the center of practice redesign. *Health Aff (Millwood)*. 2008;27(5):1219-1230. doi:10.1377/hlthaff.27.5.1219.
34. Crocker DD, Kinyota S, Dumitru GG, et al. Effectiveness of Home-Based, Multi-Trigger, Multicomponent Interventions with an Environmental Focus for Reducing Asthma Morbidity. *Am J Prev Med*. 2011;41(2):S5-S32. doi:10.1016/j.amepre.2011.05.012.
35. Kintner E, Cook G, Allen A, Meeder L, Bumpus J, Lewis K. Feasibility and benefits of a school-based academic and counseling program for older school-age students with asthma. *Res Nurs Health*. 2012;35(5):507-517. doi:10.1002/nur.21490.
36. Horner SD, Fouladi RT. Improvement of Rural Children's Asthma Self-Management by Lay Health Educators. *J Sch Health*. 2008;78(9):506-513. doi:10.1111/j.1746-1561.2008.00336.x.
37. Bruzzese J-M, Evans D, Wiesemann S, et al. Using school staff to establish a preventive network of care to improve elementary school students' control of asthma. *J Sch Health*. 2006;76(6):307-312. doi:10.1111/j.1746-1561.2006.00118.x.
38. Juniper EF, O'Byrne PM, Guyatt GH, Ferrie PJ, King DR. Development and validation of a questionnaire to measure asthma control. *Eur Respir J*. 1999;14(4):902-907. doi:10.1034/j.1399-3003.1999.14d29.x.
39. Coffman JM, Cabana MD, Yelin EH. Do School-Based Asthma Education Programs Improve Self-Management and Health Outcomes? *Pediatrics*. 2009;124(2):729-742. doi:10.1542/peds.2008-2085.
40. Bruzzese JM, Evans D, Kattan M. School-based asthma programs. *J Allergy Clin Immunol*. 2009;124(2):195-200. doi:10.1016/j.jaci.2009.05.040.
41. Lemanske RF, Kakumanu S, Shanovich K, et al. Creation and implementation of SAMPRO™: A school-based asthma management program. *J Allergy Clin Immunol*. 2016;138(3):711-723. doi:10.1016/j.jaci.2016.06.015.
42. Halterman JS, Szilagyi PG, Fisher SG, et al. Randomized Controlled Trial to Improve Care for Urban Children With Asthma Results of the School-Based Asthma Therapy Trial. *Arch Pediatr Adolesc Med*. 2011;165(3):262-268.
43. Chipps BE, Zeiger RS, Borish L, et al. Key findings and clinical implications from The Epidemiology and Natural History of Asthma: Outcomes and Treatment Regimens (TENOR) study. *J Allergy Clin Immunol*. 2012;130(2):332-

42.e10. doi:10.1016/j.jaci.2012.04.014.
44. Chipps BE, Zeiger RS, Dorenbaum A, et al. Assessment of asthma control and asthma exacerbations in the epidemiology and natural history of asthma: outcomes and treatment regimens (TENOR) observational cohort. *Curr Respir Care Rep*. 2012;1(4):259-269. doi:10.1007/s13665-012-0025-x.
45. Morgan WJ, Crain EF, Gruchalla RS, et al. Results of a Home-Based Environmental Intervention among Urban Children with Asthma. *N Engl J Med*. 2004;351(11):1068-1080. doi:10.1056/NEJMoa032097.
46. Guilbert TW, Morgan WJ, Zeiger RS, et al. Long-term inhaled corticosteroids in preschool children at high risk for asthma. *N Engl J Med*. 2006;354(19):1985-1997. doi:10.1056/NEJMoa051378.
47. Warman K, Silver EJ, Wood PR. Asthma risk factor assessment: what are the needs of inner-city families? *Ann Allergy Asthma Immunol*. 2006;97(1 Suppl 1):S11-5. http://www.ncbi.nlm.nih.gov/pubmed/16892765. Accessed November 10, 2016.
48. Child Asthma Risk Assessment Tool (CARAT). http://carat.asthmarisk.org/. Accessed November 10, 2016.
49. Aarons GA, Hurlburt M, Horwitz SM. Advancing a conceptual model of evidence-based practice implementation in public service sectors. *Adm Policy Ment Health*. 2011;38(1):4-23. doi:10.1007/s10488-010-0327-7.

APPENDIX

A. Initial Home Environmental Assessment

B. Follow-up Home Environmental Assessment

C. Initial School Environmental Assessment

D. Follow-up School Environmental Assessment

E. Care Coordination Goals Form/Tracking Sheet

F. Asthma Control Tool

G. Asthma Knowledge Quiz (Pre/Post)

H. Caregiver Needs Assessment survey

I Caregiver Satisfaction Survey

J. MDI checklist

K. Paediatric Asthma Caregiver's Quality of Life Questionnaire (PACQLQ)

L. Pulmonary function test data collection form

M. Intake Survey

N. 3, 6, 9, 12 Month Follow-up Survey

O. CARAT Survey

P. You Can Control Asthma Curriculum

Date	Protocol Change
4/19/21	Recruitment increased to 640 children with at least 600 evaluable subjects.
11/13/20	Data collection for study visits may take place remotely by telephone or video conference using a HIPAA-compliant application licensed by CHOP. In person clinic visits solely for study purposes will take place as permitted by CHOP Research Institute Policy and Guidelines. Study activities designed to take place in a school setting may take place remotely by telephone or video conference using a HIPPA-compliant application licensed by CHOP.
4/10/20	Video conferencing through a HIPAA-compliant application licensed by CHOP, including but not limited to Vidyo telehealth visits in MyCHOP, may be used for home visits that cannot be conducted in person due to health or safety concerns. For telehealth home visits, supplies will be provided to families via U.S. postal mail with caregivers providing confirmation of receipt.
4/8/20	During the COVID-19 pandemic, the study activities detailed above will be modified as follows to preserve the health and safety of participants, healthcare professionals, study staff and the West Philadelphia community. Data collection for all study visits will take place remotely by telephone or video conference using a HIPAA-compliant application licensed by CHOP. In person clinic visits solely for study purposes will be cancelled. In person clinic visits scheduled by primary care providers will not be attended by study staff. Study activities designed to take place in a school setting will be paused while schools remain closed to students and staff. These activities can resume once schools are re-opened.<br><br>Implementation of the Open Airways for Schools Curriculum will occur over three to six asthma management education sessions.<br><br>Consent discussions may be held remotely by phone or through videoconference using a HIPAA-compliant application licensed by CHOP including but not limited to BlueJeans and Skype. For consent discussions held remotely, through video conferencing or by telephone, parents will be provided with a REDCap survey link to the informed consent and HIPAA authorization to review and sign electronically. Alternatively, parents with limited internet access can provide verbal consent for participation.<br><br>Participants providing verbal consent will receive a copy of the informed consent and HIPAA authorization and documentation of verbal consent via U.S. postal mail.
3/19/20	For consent discussions held remotely, parents will be provided with a REDCap survey link to the informed consent and HIPAA authorization to review and sign electronically.
11/15/19	All randomization schemes will be prepared by the project statistical team and implemented using opaque sealed envelopes or a similar process electronically via REDCap.<br><br>The Recruitment Enhancement Core (REC) at CHOP provides assistance with recruitment plan development and may assist us in identifying and contacting potential participants using the Clinical Reporting Unit (CRU), the CHOP Recruitment Registry, social media and internal communication resources. The REC also engages community partners and facilitates outreach on behalf of the research Institute and CHOP research studies.

8/15/19	Subjects who become a ward of the state during study participation will continue to have their medical records reviewed, but will no longer participate in study visits or the completion of questionnaires. The state agency assigned legal guardianship of the participant will be asked to provide consent for medical record review of the participant during the study follow-up period. If consent cannot be obtained the participant will be withdrawn.<br><br>In the event that a parent or legal guardian regains custody and a participant is no longer a ward of the state, the legal guardian may be approached to provide consent for study participation. Once consent has been obtained, study visits can resume for the duration of the follow-up period.
6/24/19	From recommendation of the DSMB, Secondary endpoints changed from<br>-Improved communication between caregiver and provider of the intervention group<br>-Improved caregiver and patient elf-management kills of the intervention group<br><br>To –<br>*Secondary endpoints will include the following:*<br>• *The change in asthma symptom days between the intervention and the control group*<br>• *The change in school absences between the intervention and the control group*<br>• *The change in ED visits and inpatient hospitalizations for an asthma-related cause between the intervention and the control group*.<br><br>2) Efficacy analysis. Initially the data collection visits dates were erroneously numbered to include the home visits as well. Since data was not collected during the home visits, the data collection visits were termed "study visits" and the last date of collection was study visit number 5.<br>*"The primary analysis will be based on an intention to treat approach and will include all subjects randomized at Study Visit 1. The primary efficacy endpoint will be the change in asthma control between Study Visit 1 and Study Visit 5. Secondary efficacy endpoints will include the change in asthma symptom days and school absences between Study Visit 1 and Study Visit 5*. "
4/19/19	Made 2 changes to eligibility criteria:<br><br>1)Changed age from 6-12 years to 5-13 and added enrolled in elementary school.<br><br>2)Changed eligibility from 2 to 1 exacerbation requiring oral steroids in past year.
9/29/18	Changed classroom environmental assessments.<br><br>1) Changed number of schools that are invited to participate to increase the number of schools from which we could recruit.<br><br>-from "20" to "all" public elementary schools located in West Philadelphia and from "two" public charter elementary schools to "several" public charter elementary schools in West Philadelphia for a "total of no greater than 40 elementary schools".

	2) Also added to description of Federally Qualified Health centers to signify that they had to serve West Philadelphia residents<br><br>3) added to Data and Safety monitoring plan that the DSMB would be appointed by NIH<br><br>4) expanded zip codes to include 19142 and 19153<br><br>5) added spirometry if possible at first clinical visit post-enrollment<br><br>6) added that CHW other than the assigned CHW would administer study questionnaires whenever possible<br><br>7) changed timing of CHW satisfaction survey from 1 week after visit 5 to within 13-24 months post-randomization once all primary care and school interventions are completed.
6/4/18	Changed data collector to 'member of research team' from 'research assistant or primary care CHW.'<br><br>Caregiver Needs Assessment changed from 'at baseline and at follow up visits' to 'at baseline' for participants receiving the primary care intervention.<br><br>Adverse Event Forms completion changed to "as needed throughout study participation" from at "each visit."<br><br>Consent changed to include signature on a tablet device as well as paper. Also added:<br><br>*"Participants who provide an electronic signature will receive a copy of the signed document by email or a printed copy will be provided at the conclusion of the consent meeting."*
6/4/18	Changed data collector to 'member of research team' from 'research assistant or primary care CHW.'<br><br>Caregiver Needs Assessment changed from 'at baseline and at follow up visits' to 'at baseline' for participants receiving the primary care intervention.<br><br>Adverse Event Forms completion changed to "as needed throughout study participation" from at "each visit."<br><br>Consent changed to include signature on a tablet device as well as paper. Also added:<br><br>*"Participants who provide an electronic signature will receive a copy of the signed document by email or a printed copy will be provided at the conclusion of the consent meeting."*
7/13/18	Informed Consent<br><br>Added:<br><br>1) Electronic informed consent forms will be stored and managed in the study's REDCap database

	2) The electronic consent form will include the patient and caregiver's typed names and written signatures using a stylus pen.
12/18/17	Added:<br><br>Medical record review will continue for up to two years from the 12-month follow-up visit or until the data collection period for the study has ended, whichever occurs first.<br><br>Changed number of participating schools from 23 to 22.<br><br>Added a post-12 month follow up visit to protocol at which time the CHW caregiver satisfaction survey, ACQ and symptom survey would be administered.
