# Supplementary material for: Community Health Workers Linking Clinics and Schools and Asthma Control: A Randomized Clinical Trial
Source: JAMA Pediatr. 2024 Oct 21;178(12):1260–9. doi: 10.1001/jamapediatrics.2024.3967 (PMC11581744; doi:10.1001/jamapediatrics.2024.3967)
Supplement: Supplement 2. — Statistical Analysis Plan. [file jamapediatr-e243967-s002.pdf]

WePACC (West Philadelphia Asthma Care Collaborative)

Statistical Analysis Plan
Final Version

September 13, 2022

Justine Shults
Russell Localio

Biostatistics Graduate Student Kebin Yan

Division of Biostatistics
Department of Biostatistics, Epidemiology, and Informatics
Perelman School of Medicine
University of Pennsylvania

This statistical analysis plan (SAP) governs the analysis of the WEPACC factorial cluster randomized controlled WEPACC study. This SAP attempts to cover all points outlined in the CONSORT guidelines for reporting cluster randomized trials. (See Appendix) (Campbell 2010).

This SAP will be updated regularly but will be finalized formally before any analysis of outcomes.

Date of Finalization of SAP: September 13, 2022

1. Questions of interest

Stated formally, the primary question of interest is as follows. If a group of children were alternatively assigned to the following exposure groups (a= attended a school at which the specified intervention was in place AND was exposed to the specified child-level intervention, b=attended an intervention school but did not have access to the child-level intervention, c=did not attend an intervention school put received no child-level intervention, and d=did not attend an intervention school and received only usual care, then are there important differences in outcomes across the four groups?

All outcomes for the primary analysis will be measured at the child level, but in some cases (see below) will be aggregated to the school level.

2 Design

The overall design will stratify participating schools into at least 5 groups to enhance balance at baseline in school characteristics (size, location, charter status). Randomization of schools to the school-level intervention (S+) or usual practice (S-) will be stratified by these pre-defined groups. At the time asthma patients present in the clinics and consent to participating, these patients will be randomized to navigator assistance (child-level intervention) (A+) or usual care (A-), and that randomization will be stratified by school and clinic of recruitment. Allocation concealment will be preserved by randomly permuted block, and this approach will also preserve balance of intervention arms within schools over time. This randomization plan will produce a 2-by-2 factorial design, partly clustered (by school). (Table 1) Schools and children are followed over time.

Table1		Child-level intervention	
		A+	A-
School Level	S+	a	b
	S-	c	d

	none	e	f

The proposed design results in 6 groups of children. The cells a,b,c,d will permit comparison of any of the 3 combinations of school and This randomization plan will produce a 2-by-2 factorial design, partly clustered (by school). (Table 1) Schools and children are followed over time. child intervention (a,b,c) against the group that has neither(d). At the same time, it will permit marginal analysis of the effect of the school intervention (a+b vs c+d), and the marginal effect of the navigator intervention (a+c+e vs b+d+f). For each of the cells of interest, we collect baseline data on children and then follow schools and children over time. This design avoids contamination and interference through cluster randomization, but benefits from added power of a longitudinal analysis. The factorial layout permits estimation (and testing) of interaction between the school and individual interventions. It also allows for use of data from children who are from nonparticipating schools.

3 Randomization

3.1 Randomization of Schools

Schools were randomized in several waves to meet the evolving requirements of the study design.

Randomization Wave 1

For the cluster randomization of the 22 schools, we adopted the following approach.
First, because two of the 22 schools were charter schools, we immediately paired them and randomly assigned one of the pair to the intervention and the other to control.

Second, for the remaining 20 schools we implemented covariate constrained randomization to achieve balance on the three key covariates: enrollment size, facility index(%), and percent with attendance>- 95% of the time. These 3 covariates are all continuous. For that reason, conventional stratification becomes problematic because it requires categorizing these continuous factors.

There is a fundamental contradiction between the realities of cluster randomized designs (few sites/clusters from which to sample) and the needs for balance in the resulting design (site pairings that lead to comparable groups of sites). For that reason, we used simulation methods as well described in the literature (Carter 2008; Nietert 2009; Ivers 2012; Moulton 2004;) also as "restricted randomization" or "covariate balanced randomization". This approach seeks to achieve balance for each of the specified characteristics of the clusters (schools).

In each simulation we assigned the 20 schools at random into 2 groups. Then we tested the resulting groups for differences in group means of enrollment, facility score, and attendance. These were the pre-specified school characteristics needed to balance. We then compared these inter-group differences in means to pre-set constraints. In our case we chose a mean difference constraint between schools groups of 10 students for enrollment, 3 for facility percent, and 3 for attendance percent. These constraints we regarded as narrow given the range of the distributions (See Table AX). With these constraints on means alone, however, we found in testing that the

distributions could differ in range (and variance). For that reason, for both enrollment and for attendance, we added two more limits on the differences in ranges by school group: 50 for enrollment and 5 for attendance. Altogether, there were 5 constraints.

In testing we found that the yield of balanced treatment allocations across schools was 3 in 1000. For that reason, we proceeded with 2000 simulations to be able to achieve at least one balance allocation of schools. From the resulting set of balanced allocations, we randomly selected one allocation of 20 schools into two groups.

We accomplished this simulation-based randomization with a custom-written program in Stata v 15.1 after finding that published alternatives were too slow and insufficiently flexible for our dual task of achieving balanced means and ranges. The final version of randomization proceeded with a program seed to ensure reproducibility.

Table AX1. School Characteristics for balancing. N=20 schools

School Feature	Mean	Median	Min	Max
**Enrollment**	463	487	242	644
**Attendance (% of students 95%+)**	33	30	20	79
**Facility Condition Index %**	38	39	<1	79

Wave #2 Second Wave of Public Schools (November 2018)
The second wave of public schools used the same approach as Wave #1. There were 8 public schools in this wave. Using the same program and criteria as in the first wave, we used the same program (modified for only 8 schools) to arrive at 4 schools assigned to intervention and 4 assigned to controls.

Wave #3 Charter School Randomization (February 2019)
Owing to the small number of schools, the lack of data on facility condition, and the need to balance on several factors, we paired the 6 candidate schools and then when the pairs were formed, we randomized one school in each pair to intervention and the other to control.

The primary criterion for pairing was % of days with 95% or greater attendance. That criterion led to the pairing of two of the 6 schools (high attendance). Of the four remaining schools, they were pair on the basis of facility condition and enrollment. Randomization assignment then was by pair. We generated 6 random numbers via a single program from a random uniform generator (using Stata v 15.1) and assigned in order two number to each of the 3 pairs. Once assigned number, each pair then had a school with a higher random number a school with a lower random number. The school with the higher random number became the intervention school in the pair. All decisions about pairing criteria and actual pairing based on those criteria were agreed upon at a meeting on 02/21/2019. Random numbers were generated on 02/22/2019.

3.2 Randomization of children within Schools

Randomization at the child level proceeded using randomly permuted blocks with vary block sizes, as implemented using the Stata program "ralloc" *Ryan 2000). This program used randomly permuted blocks with block sizes of 2 and 4. Randomization was stratified by school and by clinic within school. There were two waves of randomization. The first occurred with the first recruitment of schools and the second with the second wave of schools.

3.3 Randomization of schools to implementation interviews

Of the 20 original public schools selected to participate – 18 school principals agreed to participate and two declined Overbrook Elementary and McMichael Elementary are both ineligible to participate in staff surveys. In addition, 3 charter schools were selected for convenience for preliminary work and test.

Across the remaining schools, the goal was to select 2 more charter schools and 18 conventional public schools. To this end, we first assigned a random uniform (0,1) number to each school. Next, we ranked these random numbers within the charter schools and within the public schools. Third, we selected the 2 (charter) and the 18 schools (public) with the highest ranks (lowest rank numbers) for inclusion in the sample. This process was done by one of the statisticians, using a pre-selected seed for the random number generator and with no contact with any of the schools nor any information about the schools other than their names.

3.4 Allocation Concealment

For the individual child level randomization, all randomization lists were generated by one of the statisticians and then delivered to the REDCap database designers. Upon each child recruitment, REDCap then produced the treatment assignment for the next child and for the school of the child's attendance. All patients had to consent to the study (and sign consent forms) before the research coordinator could elicit from REDCap the treatment assignment for that child. By this method no one on the staff other than the database manager was aware of the next treatment assignment for the next child in the stratum of school*clinic.

4 Data Collection and Management

Data will be collected, accessed and stored by the CHWs and study research team. Any paper forms will be stored in locked cabinets in the Community Asthma Prevention Program's (CAPP) badge-only accessible suite. Data collected using REDCap is accessible only by the CHWs and research study staff. Designated school administrators (i.e., principal), the school nurse and the child's primary care provider will only have access to participant health care information necessary to conduct asthma management in the schools and primary care office, but none of the research data collected by the CHWs and study staff. Information will be kept confidential under HIPPA guidelines and Institutional policies. Analysis will proceed on

CHOP computers, either secure desktop machines or the CHOP virtual computing system based on VMWare, and all data will be stored on a CHOP server in directories created for this project. Access to each dataset with identifiers will be limited to the minimum necessary to accomplish the analytic tasks.

5 Analysis – Primary

5.1 Specific Aim 1. Compare the effectiveness of all combinations of clinic and school based interventions to improve asthma control and reduce symptom days.

We have selected a factorial design because of its enhanced statistical power and its ability to estimate contrasts of interest, even within the framework of a cluster randomized design. Chakraborty (2009). Collins (2014). The added complexity of a conventional within-school randomization (child-level intervention) crossed with cluster-randomization (school-level intervention) can be handled with the proposed model alternatives, each with a different set of assumptions, to demonstrate the robustness of the findings to model specification. (Peters 2003). Our statistical methods will also estimate variation across schools in the effect over time. (Fitzmaurice, Laird, & Ware, 2011; Gelman & Hill 2007; Hayes & Moulton, 2017).

We propose and pre-specify three alternative approaches, each of which has been used in conventional cluster randomized design. Each approach has assumptions, strengths and weaknesses. We intend to report the results of all three approaches, provided that models will converge to results, in order to assess robustness of findings to model choice. We do not consider use of the three approaches to be multiple comparisons. Rather, we consider the use of multiple approaches to assess this additional component of variability.

5.1.1 Outcomes

Outcome and their priority appear in Table 5.1.1

Table 5.1.1

Study Outcome- Listed in Order of Priority	Measurement times (month) 0	3	6	9	12
Asthma (Asthma control questionnaire) PRIMARY	X	X	X	X	X
Asthma quality of life	X				X
Number of hospitalizations	X				X
Number of emergency department visits	X				X

On or off controller medication	X				X
Courses of oral steroids	X				X
Number of school absences	X				X
ICD-10 J45.XX Diagnosis-based encounters- to define relevant office, hospitalization, ED visits	X				X

School absences. Each child will have two semesters (one years) before and after the start of the intervention from which to calculate absences. Absenteeism data will ideally come from the schools. If schools are unable to provide absenteeism data, self-reported data will be utilized. We propose to evaluate the reliability and completeness of school absences data before, and independently from, formal analysis of the association of this and other outcomes and treatment assignment.

5.1.2 Measuring outcomes over time - irregular measurement times

Longitudinal model

Although the protocol calls for regular measurement times (Juniper's **Asthma Control Questionnaire** is a validated survey and will be administered by the CHW at baseline, 3 months, 6 months, 9 months and 12 months), in all likelihood these measurements will have the following characteristics. (1) They will not be administered at 3,6, 9 and 12 months. For some the schedule will lag. (2) Some children will miss one or more of these measurements.

Although some RCTs assume that measurements occur at the protocol-defined time, even when they do not, there is no reason for, and we shall deliberately avoid, that ad hoc practice.

We shall model the time of each measurement as the number of months (including factional amounts) from date of randomization. Time we shall model flexibly with splines. [Here we can use cubic splines as needed).

Choices for knots. We shall assume knots at time 3, 6 and 9 months, the intended dates of measurements.

With time measured flexibly, and with a statistical model that includes time by treatment (4 level) interactions, the spline model will allow for estimate of differences (or ratios) of outcomes at any point along the time (x) axis by treatment group.

Covariates -- selection and balancing

In theory randomization at the child level should balance on observed and unobserved factors. With children spread over 36 schools, and with chance differences across school, imbalances in child characteristics are possible at the school level but are not likely at the treatment assignment level (the four cells a, b, c and d). In addition, dropout can undermine the balance achieved in theory and in practice by randomization.

Key child-level covariates appear in Table 5.1.2.

Table 5.1.2 Covariates – baseline descriptions

**Child level factors**	**Units of measurement or categories**
AGE	Subgroups: 5-11; 12-13
GENDER	Male<br>Female
Body mass index<br>(weight in kg)<br>(height in cm)	Collected and/or calculated by age-sex standardized percentile and/or z score.<br>Both percentile and z will be used
HOUSEHOLD CHARACTERISTICS (descriptive only)	(1) Single Parent/Caregiver vs Multiple Caregivers<br>(2)Number of Adults in household<br>(3) Number of Children in Household –
Home environment	Number of bedrooms
Smoking	Smokers or none in home
Caregiver Education --<br>Highest level achieved by primary caregiver	Less than HS<br>HS+<br>Graduate Level
Baseline Healthcare utilization	
Oral Steroid courses in the last year<br><br>Use as continuous – But describe in categories	None<br>1 course<br>2 courses<br>3-5 courses<br>>5 courses
ED Visits in the last year<br><br>Continuous – Describe in categories	0, 1,2, 3+
Inpatient visits in the last year<br>Continuous—Describe in categories	0,1,2, 3+

Data on height and weight, and BMI, and BMIz, will be downloaded from EPIC. BMI categories of interest are <85th percentile, 85th to 95th percentile, and 95% percentile or greater. Height is not done at each encounter. For that reason, height must be carried forward from the last available measurement time. This practice, though common, can result in a bias toward higher than actual BMI over time. Adjustments (e.g., interpolation) of height for measurement times between height measures is possible. If any interpolation of height, we shall do so without regard to measures of outcome.

For that reason, we shall first examine balance across the 4 treatment groups in child-level characteristics. To that end, we shall use conventional balancing diagnostics, such as comparison of percentages of patients by categorical factors and by standardized differences for continuous factors, and/or comparisons of distributions. All will be supplemented by graphical display. These diagnostics are programmed and explained in the R program "cobalt" (Greifer 2019).

Treatment assignment model

Covariates that do not demonstrate balance will be included in an initial multinomial logit model (with the 4 treatments as outcomes). Then using generalized propensity scores, we will develop inverse probability of treatment assignment weights. This approach will then result in estimates of average treatment effects in the response model.

Response model—to estimate expected values over time

The response model will be a weighted longitudinal model using splines for time and time by treatment interaction terms. This model will result in the estimation of expected values at any given time post randomization for any of the four treatment categories.

5.1.2 Patient-level analysis – mixed effects models

First, linear mixed effects models with random intercepts and slopes for school, and fixed effects for the school-level intervention, time, and time-by-intervention interaction (the estimate of interest) will be applied. Likewise, the main effect for the child-level intervention and a time*intervention interaction term will be included in the model, and variation across multiple measurements on a child within a school will be accounted for by child-level random effects. This approach then becomes a three-level model with explicitly modeled random effects at the school and child levels.

Synergy of the effect of the two interventions will be estimated by the 3-way interaction between time, the school-level intervention, and the child-level intervention along with all corresponding two-way interactions.

Models will use numerical quadrature with least 12 quadrature points. Models with 16 quadrature points will be used to check sensitivity of results to numerical integration.

This and other individual-patient-level methods can be more efficient (smaller confidence bounds) than cluster (school)-level models. (Hayes and Moulton, section 11.4.2)

We shall exploit mixed effects models to estimate the degree of variation of effects across the 36 schools, both the 18 intervention schools and the 18 control schools by estimating the random slope component of variance and asking whether the observed variation across schools is greater than that expected at random.

5.1.3 Patient-level analysis – marginal models

Second, marginal models using generalized estimating equations (Fitzmaurice 2011) will produce robust estimates that adjust for clustering at the school level. These methods tend to be robust for non-continuous outcomes (non-identify link models) and those outcomes will likely apply to our outcomes of asthma control (possibly a log gamma model) and number of symptom days (possibly a log link Poisson or negative binomial model). In contrast to mixed effects models, marginal models do not rely on numerical integration. In addition to generalized estimating equations, quasi-least squares regression (Shults and Hilbe, 2014) will also be implemented. Quasi-least squares regression is an approach for estimation of the correlation parameters that is in the framework of generalized estimating equations; it allows for application of correlation structures that were previously unavailable for generalized estimating equations and can sometimes be implemented when generalized estimating equations fails to converge.

Marginal models tend to work poorly for designs with small numbers of clusters. In our case, the number of clusters (36) is relatively large. Nevertheless, we shall implement at least one method for adjusting for a number of clusters that is not large, as implemented in Stata's program xtgeebcv program (Gallis 2019) and R's program "saws" (Fay and Graubard 2001).

5.1.4 School-level analysis -- randomization-test-based methods.

Third, assumption-free, randomization-test-based (permutation test) methods that do not rely on assumptions of parametric models will also be applied. (Berger, 2000; Good, 2005; Small, 2008; Tang 2009; Rosenbaum 2002). (Also see Hayes and Moulton 2017)

We will use conventional levels of statistical significance (p=0.05) for all pre-specified comparisons for these aims. We shall not apply Bonferroni corrections for estimates from different modeling methods that use the same specification.

We shall report all results to confirm consistency of findings and their robustness to model specifications. Variability of the intervention effect across schools will reflect consistency of intervention effects and thus generalizability in new settings. Both mixed effects models and permutation-test methods (Lee 2012) will estimate variance components to support generalizability. This approach will also be used to account for change over time across schools, and to assess sustainability of the proposed interventions.

5.2 Adjustments for sparse cells

In the event that one or more schools fails to fill the cells a, b, c , d with at least one child, we shall make the following adjustments in the definition of strata (clusters). Any school that contains zero cells will be joined with the "nearest" school assigned to the same intervention group, where nearest is determined by the same school randomization factors. This pooling of schools will be done without knowledge of the outcomes. At that time, and for all analyses of child outcomes, the pooled schools will be treated as one for analysis. Implications for analysis of association of implementation and outcome will require discussion.

If no children were recruited in any school, then that school will be dropped from the analysis.

If a school lacks a child in one of the two child-level randomization groups, then the school will be lumped with another school in the same treatment arm and in the same randomization stratum, or the closest stratum, and if there is more than one such school, then with the school that has the smallest number of recruited children.

5.3 Missing data

Missing data on covariates will be handled using formal multiple imputation as needed (Little 2002; 1992; Molenberghs 2007). The proposed mixed effects models make limited assumptions of "missing at random" (MAR). To the extent that children are lost to follow-up, we shall include in our models covariates that are associated with the probability of dropout, to limit confounding by dropout. We anticipate no school withdrawals. The primary analysis will be "as randomized" (also called intention to treat). Reporting will follow the CONSORT guidelines for randomized studies and cluster randomized designs. (Altman 2012; Elbourne and Campbell 2001).

5.4 Dropout and switching

All analyses will follow the principles of "intention to treat" (as randomized) for both the school and child level randomizations (S+ vs S- and A+ vs A-). The following special rules apply for unusual situations.

(1) Child dropout or loss to follow-up

If a child leaves the study, his or her data will be captured and used until dropout. There will be no change in the A+ A- assignment.

(2) Child remains in the study but leaves a named school

If the child remains in the study but leaves a named school (cells a,b,c,d), she will be moved to the cells e or f as appropriate. This change in designation will not affect the contrast of A+ vs A-

within a school designation. Data that accrues while the child is in cells a,b,c, or d will be linked to that school until the time of the switch for contrasts of S + vs S-.

After a switch from a,b,c or d, to e or f, the child-level observation will apply to the e and f cells.

(3) Child randomized while in e or f and moves into an enrolled school (cells a, b,c,d) then his or her follow-up is attributed to the e or f cell until the switch, and then to a,b,c or d cells thereafter. Both sets of longitudinal data can share the same baseline measures of outcome.

If this switch occurs, the child will be recoded separately according to the "S" allocation.

5.5 Original power estimates for planning purposes

Power calculations are for planning purposes only. The following paragraph is historical information only.

The following description and table report power calculations as originally planned for the study design. Alternative power calculations for fewer patients appear later.

All power estimates are based on custom programmed simulations. All power estimates represent changes over time in the primary outcomes (asthma control) in units of standard deviation of change. For translation into clinical terms, the minimally important difference (MID) for children on the Juniper's Asthma Control Questionnaire is 0.5 standard deviation units, which equates to 0.4 units on the questionnaire scale (for children ages 6 to 17 years). (Nguyen 2014). The power simulations assumed a modest degree of variation in outcomes across schools, which equates to clustering of children within schools. For an overall effect size of 0.5 standard deviation units, we assumed that individual schools would vary from no effect (0.0) to a large effect (1.0sd units). This degree of variation corresponds to a random cluster (school) effect of 0.025 sd units, and an intraclass correlation coefficient (ICC) of 0.06. With an average of 28 children per school, this variability equates to a design effect (variance inflation factor) of about 2.5. As the table shows, the contrasts for the navigator intervention are powerful to show even small effects, because they are within-cluster comparisons and therefore are influenced if at all only slightly by clustering of children within schools. Across school contrasts, of the effects of the school intervention, remain powerful for MID changes.

Table Y. Power for contrasts of interest.

Specific Aim	Contrast	Contrast (Table cell)	2/21/2023	Stipulated Effect size(sd)			Estimated Power
				A	S	A,S	
1a	S+ v S-	a,b vs c,d	a,b vs c,d,e,f	0.5	0.5	0.1	0.90
1a	A+ v A-	a,c vs b,d	a,c,e vs b,d,f	0.25	0.25	0.1	0.95
1b	S+A+ v S-A+	a vs c	a vs c,e	0.5	0.5	0.1	0.95
1c	S- A+ v S+A-	b vs c	b vs c,e	0.75	0.25	0.0	0.92
1d	S+A+ v S-A-	a vs d	a vs d,f	0.25	0.25	0.1	0.96
	S- A+ v S-A-	c vs d	c,e vs d,f	0.35	0.5	0.0	0.90
	S+,A- v S-A-	b vs d	b vs d,f	0.5	0.5	0.1	0.90

20%	Subgroup						
2a	S+ v S-	a,b, vs c,d	a,b vs c,d,e,f	0.5	0.6	0.1	0.83
2a	A+ v A-	A,c vs b,d	a,c,e vs b,d,f	0.5	0.6	0.1	0.88

A=Navigator effect; S=School Effect; AS= interaction; "+" = , "-" = control
Cells e, f are not included in this table for simplicity and to be conservative.

6 Analysis of subgroups

Specific Aim 2: Explore moderators and mechanisms of effectiveness of the asthma navigator and school-based asthma therapy interventions.

6.1 Heterogeneity of treatment effect -- Effect modification (moderators)

Effect modifiers are factors that reflect differences in the effect of the intervention across subgroups of children and/or schools. When taken together these child or school level factors can describe those children or schools that are predicted to do better or worse than the reference group of schools or children. “For a clinical trial that establishes treatment efficacy in its overall population, investigating congsistency of treatment effects across subgroups is important for interpreting the efficacy findings and consequently for determining the appropriate population for treatment use.” (Alosh 2017) Effect modification is to be distinguished from program interaction, which in this factorial-design trial will estimate the degree to which the combination of child and school level interventions achieves synergy (or antagonism).

As subgroup analysis is fraught with problems of multiple comparisons, we shall adhere to the following principles for analysis and reporting: pre-specification of subgroups of interest, focus on subgroups that are clinically important as suggested by prior research, and attention to considerations of statistical power for identifying subgroups.

6.1.1 School level characteristics

School characteristics appear in Table 6.1a. Because the number of factors when cross classified is large (3*3*4=36) relative to the total number of schools in the sample, effect modification by school will be based on post-hoc ordering of schools by their predicted level of improvement, standardized by child characteristics. With mixed effects models that include random intercepts and slopes for school, we propose to order each school in the intervention group by its “prediction” (the combination of expected value and random effects) at both baseline and at month 12. This ordering will (a) account for measurement error in the outcome of interest (the main outcome is asthma control), (b) adjust estimates by the number of observations at the child level over time, and (c) permit consideration of both the level at baseline and the trend over time of the degree of asthma control. By contrasting for each school the change over time in predicted asthma control levels for children enrolled in the study from that school, we can identify the schools with the most improvement and the least improvement. A description of the characteristics of the high (and low) performing schools will then support conclusion (and generate hypotheses) about school-level factors related to the success (or failure) of the intervention.

Table 6.1a

School Subgroups as candidate effect modifiers	HYPOTHESIS
(1) FCI,<br>(2) school absenteeism rate,<br>(3) poverty level	Children going to schools with poorer FCI, higher school absenteeism rate and greater # of households living below poverty level will demonstrate the least amount of improvement with school intervention
(1) Climate,<br>(2) leadership,<br>(3) positive attitudes	Schools will improve more from the intervention if at baseline they have more positive climate, stronger implementation leadership, and more positive attitudes towards evidence-based practice.
(1) Charter vs Non-charter<br>(2) < 500 students vs > 500 students<br>(3) Facility score Index<br>(4) Census tract	H1: students who attend charter schools will achieve greater reduction of missed school days during the intervention<br>H2: schools with lower FCI's will have more asthma triggers which will lead reduced improvement in missed school days<br>H3: improvement in school absences from the intervention will differ based on census tract of school (effect modification by census tract)

Descriptive analysis of schools:

Charter school performance remains of special interest. For other school characteristics, we will look at the schools in order of their performance and then describe any differences. Owing to the small number of children in some of the schools, the relatively small number of schools relative to the number of school characteristics of interest, this exercise in looking for effect modification by school must inevitably be entirely exploratory and descriptive.

Statistical analysis of effect modification by charter school status.

Nevertheless, the number of children recruited from charter schools as of 12/2019 has been substantial. For that reason, we shall revisit the feasibility of comparing charter vs non-charter schools formally once total counts of evaluable schools and children are complete. Decisions about feasibility will be made before considering outcomes. (12/21/2019)

6.1.2 Patient level subgroups

Owing to the number of children in the study, subgroups defined by child-level factors will have large sample sizes and more power to identify and estimate subgroups and treatment effects within those subgroups using statistical approaches. However, there are far too many patient-level factors to treat as levels to create subgroups. For that reason, we shall implement latent class analysis to group children into subclasses.

Table 6.1b. Child-level factors for identifying subgroups for exploring heterogeneity of treatment effects
[These will be reduced to a small set of groups using latent class models.]

Child-level variables that reflect subgroups	Groups	Alternative HYPOTHESIS	CRF AND REFERENCES
Child			
AGE	Subgroups: 5-11; 12-13	H1: Younger children will have better asthma control improvement from the interventions than older children.	
Sex	Male<br>Female	H1: Sex will not affect improvement from the intervention. h<br>(dropped this Nov 2019)	
BASELINE ASTHMA CONTROL	Well-controlled<br>Not well controlled	H1: patients with poor control at baseline will have more improved asthma control at 12 months as compared to patients who are well-controlled.	
ED visits (prior year at baseline)	0,1, 2 3+		
Inpatient visits (prior year at baseline)	0,1,2,3+		
Oral steroids COURSES IN PAST YEAR (at baseline)	0,1,2,<br>3-5, 6+	H1: patients with more than 3 prednisone in the previous year will have better response to the intervention for asthma control at 12 months.	
Home and caregivers			
HOUSEHOLD CHARACTERISTICS	(1) Single Parent/Caregiver vs Multiple Caregivers<br>(2) Rent vs. Own		

Primary Caregiver-HIGHEST LEVEL achieved	Less than HS<br>HS+<br>Graduate Level	H1: children of primary caregiver with less education will have poorer improvement asthma control than those with more education	
Triggers at home			
TRIGGER EXPOSURES AT HOME	0 exposures<br>1, 2, 3+ exposures	H1: Children with 3+ asthma triggers in their bedroom at baseline will be less susceptible to improvement over time from the intervention.	
HOME OWNERSHIP STATUS	Own<br>Rent	H1: There will be a difference in reduction of asthma triggers from the intervention by the status of home ownership	
Neighborhood and environment			
NEIGHBORHOOD CHARACTERISTICS	L & I violations<br><br>Childhood Opportunity Index	H1: children who live in neighborhoods with higher L& I violations will have more symptoms and will improve less from the intervention.<br>H2: children who live in neighborhoods with lower opportunities will have fewer reductions from the intervention of missed school days and symptoms	

6.1.2.1 Record linkage for patient's home address and ADI 12-digit FIPS code

Effective Spring 2020, CHOP EPIC system will geocode all patient's addresses into 12-digit FIPS codes. These codes will be downloaded from EPIC for each patient for the patient's address at the time of enrollment. Following changes in address will be too complex, and for that reason the address at the time of enrollment will be used. Record linkage with the ADI will proceed based on the 12-digit FIPS codes in EPIC and the ADI database.

Using this information, we will use the ADI index as a proxy for the neighborhood characteristics listed in Table 6.1b.

Patient utilization at baseline: Value in looking at by severity of exacerbation as the relevant outcome that is subject to modification. Whether to hospitalize a patient or to go to the emergency department are often judgment calls and therefore might be vary with the same level of exacerbation. Nevertheless, although baseline utilization might modify the effect of the intervention, the direction of that modification is uncertain. This analysis is therefore hypothesis generating.

We will reserve looking at outcomes until we have settled on candidate effect modifiers and using (or developing) indexes or summary measures.

We shall investigate treatment effect heterogeneity by using carefully pre-specified factors measured at baseline. Post baseline factors that might affect outcomes will fall under the topic of "mediation" (see below) and will be handled differently.

Because these key potential effect modifiers vary within the unit of randomization (school), these contrasts are essentially within-cluster comparisons with good statistical power (see below). Effect modification (moderation) can be estimated using the mixed effects and longitudinal models outlined previously without additional complexity. In all cases, effect modifiers (moderators) will be specified before the start of analysis to address concerns about multiple comparisons.

As of this writing, guidelines remain for publication on proper characterization of subgroups and the analysis of effects by subgroup. In brief, the following principles apply.

6.1.2.2 Estimating effect modification – scale dependency of interactions

Models for outcomes might be both additive (for asthma control) and multiplicative, as for use of log link generalized linear models. The contrasts of interest for permutation-test-based methods might also be additive (differences across the four treatment groups of differences in expected values over time).

Interaction (and effect modification) are scale dependent. A multiplicative model (log link) that has no interaction can give use to results that show effect modification on the additive scale. For that reason, we define "effect modification" to be on the additive scale for the key endpoints of

change in asthma control scores and number of days of symptoms.

Estimation will follow the same principles and procedures as for the main analysis. Longitudinal data analysis models with splines for time will allow for estimation of expected values at time of randomization and at any time post randomization. For a single subgroup, the effect of the intervention (4 levels) can be compared by estimating eight expected values: four at time 0 and four at the endpoint time of interest. Contrasts across levels of the intervention and over time are estimated by subtraction.

6.1.3 Estimating confidence bounds for subgroups of interest

For ease in implementation and clarity of reporting, all confidence intervals will be estimated by bootstrap resampling (at the level of the school), stratified by school level treatment to maintain constant the number of schools in each treatment category (eighteen). Resampling for longitudinal models will be done with 399 or 999 (if computational limits allow) samples to estimate percentile confidence bounds. (The 399th and 999th order statistics correspond to the 2.5^{th} and 97.5^{th} percentile bounds that form a 95% confidence interval). School level resampling will preserve the clustering and avoid overstating statistical significance.

For estimating confidence bounds to compare two subgroups will require a minimum of 16 expected values: four at time 0 and four at the endpoint time of interest for each of two subgroups.

6.1.4 Data reduction through use of latent class methods

Each of the patient level factors can be combined into latent classes to arrive at a tractable number of levels for use in estimating heterogeneity of treatment effect. (Lanza 2013).

Examples appear in Zhang (2018) on defining subgroups with different clinical outcomes.

6.1.5 Statistical software considerations

Methods for longitudinal analysis apply equally well to comparison of subgroups and to assess heterogeneity of treatment effects. Latent class analysis is available in SAS and more recently in Stata v15 et seq in “gsem”, a program that will allow for different types of variables using to create latent classes. Python may also be used in sensitivity or primary analyses (Couronne, Vidailhet, Corvol et al. 2019).

6.1.6 Statistical Power for effect modification:

Owing to the absence of software for testing interaction effects, these estimates are based on our simulations conducted during the study design phase using Stata v 14.1. For a subgroup as small as 20% of the expected students (n=600) or 120 children, our design has 88% power to detect the

effect of the navigator and 83% power to detect the effect of school for effect sizes of 0.5 for navigator, 0.6 for school, and a small interaction of 0.1.

This preliminary estimate does not anticipate issue of multiple comparisons. To the extent that the study reports differences in outcomes by baseline groups, simple Bonferroni corrections will apply.

6.2 Mediators and mechanisms -- Specific Aim 2b

Specific Aim 2b. We will investigate mechanisms- linkage of communications between all sectors, environmental (school and home) remediation steps, daily participation in SBAT, care coordination contacts achieved, correct MDI technique. A schematic of the mechanisms appears in Figure 6a

Figure 6a. Conceptual Framework

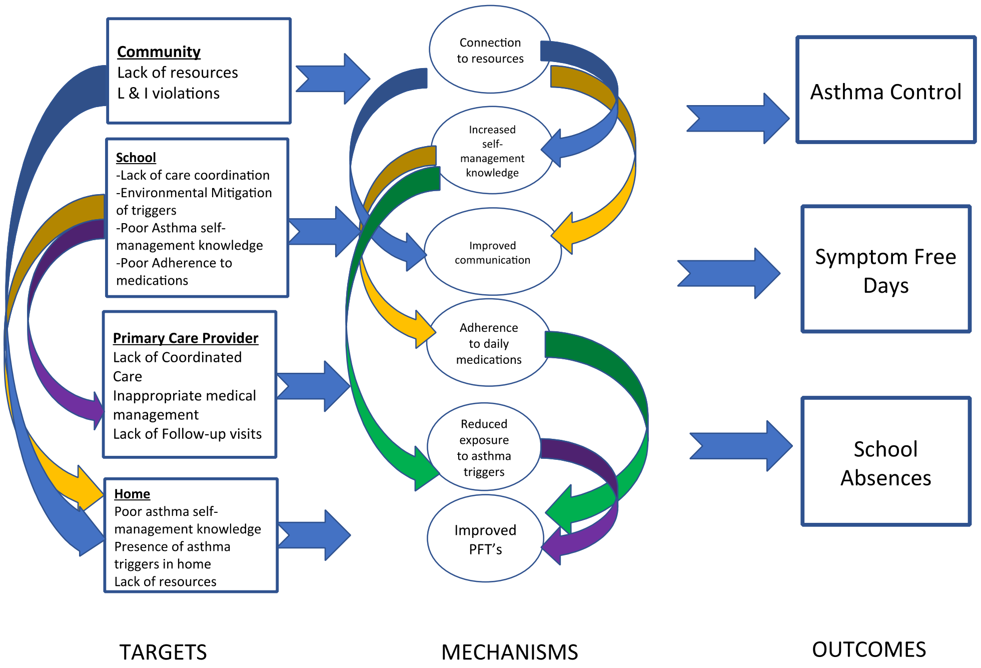

The intervention will in theory improve school, primary care provider, and home targets via the mechanisms: PFT, exposure to triggers, adherence to medications, self-management knowledge, and connection to resources.

6.2.1 Goals of mediation analysis

Mediation analysis can help to understand the etiology and mechanisms of change that give rise to the observed improvements from the intervention. It can also support measurement of direct effects of the intervention (on outcome without having an effect on the mediator) and indirect effects of the intervention (which produce outcomes through the mediating factor). (MacKinnon 2007) A key constraint for longitudinal designs is that candidate "mediators" should follow the sequence in time of exposure→mediator→outcome. If a candidate "mediator" occurs or could occur after the measured outcome, we will not consider that candidate. If the candidate mediator occurs contemporaneously with the outcome, and if its measurement is of interest, we shall consider it as "surrogate outcome".

6.2.2 School-level factors

The Consolidated Framework for Implementation Research (CFIR)

CFIR is an effort to synthesize the frameworks in implementation science. The five major domains that comprise the CFIR include:

(1) intervention characteristics (i.e., the features of an intervention such as School Based Asthma Therapy; SBAT);

(2) outer setting (i.e., the economic, political, and social context within which a school exists);

(3) inner setting (i.e., the organizational setting - school);

(4) characteristics of individuals involved in implementation (e.g., nurses, teachers); and

(5) the implementation process. In our specific example, at the nurse level, low self-efficacy or lack of knowledge about best practices for managing asthma in schools may contribute to low implementation of best practices. At the inner setting, challenges regarding availability of resources (e.g., forms related to asthma treatment, asthma medication) and organizational support (e.g., support from front-desk staff and management) may be present. Outer setting barriers may include lack of uniform policies to support implementation. Intervention characteristics such as ease of use are also relevant

6.2.3 Candidate mediation measures

Table 6.2.3

Mediating Factors	HYPOTHESES	Associated Variables and Instruments for Mediating Factors
Patient-level factors		
SELF-MANAGEMENT KNOWLEDGE	H1: The intervention will lead to improved caregiver self-management, which in turn will lead to improved child outcomes.	Asthma Knowledge Quiz Score (pre- and post-intervention)
SELF-EFFICACY	H1: The intervention will increase caregivers' self-efficacy, which will then lead to better child outcomes	Child Risk Assessment Tool
ADHERENCE	H1: The intervention will lead to greater chances of a child's receiving controller medications at least 80% of school days, which in turn will lead to better outcomes	SBAT Weekly Progress Form
CONNECTION TO RESOURCES	H1: The intervention will lead to more families making connections with resources, which in turn will le d to better outcomes.	Baseline Visit Checklist; Home Visit Follow-Up Checklist; Follow-Up Visit Checklist 3, 6, Months; 12-Month Follow-Up Checklist
Asthma TRIGGERS	H1: The intervention will reduce the number of triggers in childrens' bedrooms, leading to improved child outcomes	Initial Home Environmental Assessment; Follow-Up Home Environmental Assessment
Pulmonary Function Tests (PFTs)	H1: Children in the A+S+ will have improved PFT's, which will lead to better child outcomes.	Measured by spirometry every 3 months.
CARE COORDINATION GOALS	H1: The intervention will lead to more families who reach 80% care coordination goals, which will lead to better child outcomes.	Caregiver Goal Tracking Tool

Mediating factors, continued)	HYPOTHESES	Associated levels for Mediating factors
**School level factors**		
IMPROVED KNOWLEDGE	The school intervention will lead to improved knowledge of FOR SCHOOL STAFF (SCHOOL ADMINISTRATION EDUCATION) AND STUDENTS (OPEN AIRWAYS), and then to better child outcomes.	Asthma 101 pre- and post-test scores<br>OAS pre- and post-test scores
ADHERENCE	The school intervention will improve the quality of School Based Asthma Therapy, and in turn to better child outcomes.	Number of days school open
COMPLETED MED1 FORMS	The school intervention will lead to more ACCURATE PRESCRIPTIONS IN SCHOOL and in turn to better outcomes.	School Nurse Baseline Checklist; School Nurse Follow-Up Checklist
IMPROVED PRIMARY CARE-SCHOOL COMMUNICATION	The school intervention will lead to improve communication between school and primary care clinicians (HOW MEASURED?) and then to better child outcomes.	School Nurse Baseline Checklist; School Nurse Follow-Up Checklist

6.2.4 Mediation models

This aim explores potential mediating variables (M) on the effect of the intervention (A=navigator; S=School) on student/patient outcomes (Y) with a goal of understanding which components of the intervention, and one or more dimensions of the intervention, proves to be ineffective, then the mediation can help to identify the reasons. Mediation analysis requires many and much stronger assumptions. We shall follow the counterfactual approach (Valeri 2013) to mediation to take advantage of recent statistical developments in causal inference (Lynch 2008; MacKinnon 2007; Bind 2016; Ten Have and Joffe 2012; Imai Keele and Tingley 2010; Preacher 2015; VanderWeele 2015; VanderWeele 2016). Because children are not randomized to levels of M, and to avoid the resulting potential bias, we will adopt two approaches: (1) Marginal structural models, in which mediator variables are modeled as functions of baseline covariates, have been developed for parametric estimation of direct and indirect effects without the severe assumptions of prior methods (Preacher 2011; Petersen 2006; Sobel 2008; VanderWeele 2009); (2) Simulation-based methods (Imai 2010) offer practical alternatives. These methods apply to clustered designs as well as traditional randomized designs (Stapleton, 2015; Ten Have, 2004; VanderWeele 2010). Using the nomenclature of recent reviews, the school intervention of our factorial design is 2-1-1, in which the intervention applies at level 2 (schools) and mediators and outcomes are measured at level 1 (child; Talloen 2016). Using these methods, we can decompose total intervention effect into the two components: natural direct effect (NDE= the effect of the intervention on child outcomes not mediated), and the natural indirect effect (NIE= the component of the intervention

that seems to proceed through the measured mechanisms). As such, Total effect = NDE+ NIE (Peterson 2006; VanderWeele 2015). Within this framework, we can also estimate effects of multiple correlated mediators ((VanderWeele and Vansteelandt 2014)

6.2.5 Assumptions behind mediation models

Six assumptions must be satisfied for causal inferences for mediation and unbiased estimation of direct and indirect effects: (1) no A→Y and (2) no A→M confounding. These two assumptions will be met via design (randomization) and careful control of child-level covariates. Additional assumptions include: (3) no M→ Y confounding, and (4) no M→Y confounder that is affected by A (exposure). Although these last two assumptions are not required for estimates of overall effects of the intervention (A→ Y), they are essential for estimating direct/indirect effects even with randomized A and S. For that reason, our design will collect data on possible confounders: grade level, child gender, and comorbidities

Two additional assumptions are noninterference at both the individual and school levels (also called *no spillover effects)*. To limit noninterference at the school level (assumption 5: intervention at one school cannot influence the outcome at another (Sobel 2006)), we shall instruct each school to work alone and not to contact or share ideas with other schools. Noninterference at the child level (assumption 6) will require that children not share with each other their interactions with and information from navigators to lead to interference in the M→ Y relationship. Thus, we anticipate good compliance with key assumptions for unbiased mediation analysis.

6.2.6 Sensitivity analyses

Sensitivity analyses are absolutely essential for mediation analysis to assess the possible effects of confounding and interference. (1) For estimating the A→Y intervention effect, we shall use simulations to estimate how influential a confounder would need to be to produce a true A→Y association of zero, or to lead to a confidence interval that includes the null (non-significant result). (2) To examine sensitivity to violations of assumptions #3, #4 (no M→Y confounding) we shall assess how strong an unobserved confounder (or confounders) would need to be in order to: (a) eliminate (reduce to zero) the estimate for a direct effect, and (b) render non-significant that estimate. Methods are described by Imai (Statistical Sci 2010), VanderWeele (2016), VanderWeele (2015) and Ding (2016a; 2016b). Our team of investigators will continuously monitor emerging statistical methodological on mediation in complex randomized designs.

6.2.7 Statistical software

Statistical software for mediation continues to develop. Standard packages for both estimation and for sensitivity analyses are now available in Stata v 14.1 (Hicks 2011) and R (Tingley 2015; 2014) for the implementation of the methods of Imai (2010). Extensive macros in SAS for parametric methods are implemented by Ding and VanderWeele (2016). Stata's "gformula" package also

estimates natural direct and indirect effects (Daniel 2011) via simulations and this approach continues to find applications for mediation analysis (Wang 2015). The R package program 'medflex' supports mediation analysis to estimate natural effects. (Steen 2015)

To determine whether implementation fidelity and participant engagement are associated with outcomes, we propose to describe the impact of these factors on the outcomes in the intervention schools, with special attention to variation in outcomes across schools. These associations will not benefit from randomization (fidelity and engagement are post randomization interim process endpoints). For that reason, our assessments will be largely descriptive and qualitative, and will include follow-up contacts with the schools where school-level outcomes do not coincide with measured fidelity and engagement.

6.2.8 Qualitative methods for mediation

Our Implementation Aim specifies: *Specific Aim 3: Use mixed methods to explore implementation determinants and outcomes in the school intervention that promote effectiveness, fidelity and sustainability.* We use the CFIR to guide our implementation determinants of interest which we are measuring both quantitatively and qualitatively. With regard to quantitative measurement, we are collecting school climate, leadership, and attitudes towards SBAT which are all determinants within the CFIR framework during implementation and sustainment. We also used the CFIR to inform our interview guides in querying around barriers and facilitators to implementation.

6.3 Statistical power for mediation analysis

The original study design included the following power calculations:

For this Aim, we rely on two programs contributed to the R statistical package. We again assumed 600 evaluable patients across 23 schools, and a design effect of about 2.5. In brief, David Kenny's program "PowMedR" (Kenny 2014) suggests that power is adequate to detect an effect size (in terms of standard deviations) of 0.25 sd for both the effect of the navigator intervention (A) on the mediator (M) and the effect of M given A on Y (conditional effect of M on outcome). Likewise, for the effects of school (S), with a design effect (variance inflation) of 2.5 (see estimate above based on ICC), power remains adequate for detecting an effect size of 0.5, which is the minimal important difference for asthma control For confirmation, the R package "medssp" (VIttinghoff 2015) suggests adequate power (greater than 0.8) to detect effect sizes of 0.25 jointly and separately for both the association of A (navigator) and M (effect of intervention M) and the effect of M on Y, and to detect effect sizes of 0.6 for the association of S (school based intervention) and M, the difference owing to the large design effect (variance inflation) arising from the clustered randomization of schools.

These calculations do not consider the impact of multiple comparisons and the change in the critical alpha level for these estimates. Power will quickly degrade with more than a few candidate mediators (updated 07/16/2019)

7. Analysis for implementation

See Penn IRB. (IRP PROTOCOL#: 830021. Adoption and Implementation of West Philadelphia Asthma Care Implementation Plan)

8. Power Calculations - revised 04/2019

Primary outcomes

Initial designs included formal statistical power calculations. Based on initial recruitment rates, the initial power calculation (for design and planning purposes) has been replaced by the following update (dtd 04/14/2019). Power calculations were done for planning purposes only, and they are here for historical record keeping purposes only. They are not appropriate once the study has been done. What happens if the number of children to be recruited falls below originally planned?

.

Factorial design		Child-level Intervention	
		A+	A-
School Level	S+	a	b
	S-	c	d
	none	e	f

The proposed design results in 6 groups of children. The cells a,b,c,d will permit comparison of any of the 3 combinations of school (S+) and child (A+) interventions (a,b,c) against the group that has neither(d). At the same time, it will permit marginal analysis of the effect of the school intervention (a+b vs c+d), and the marginal effect of the navigator intervention (a+c+e vs b+d+f).

For each of the cells of interest, we collect baseline data on children and then follow schools and children over time. This design avoids contamination and interference through cluster randomization, but benefits from added power of a longitudinal analysis. The factorial layout permits estimation (and testing) of interaction between the school and individual interventions. It also allows for use of data from children who are from nonparticipating schools.

This report focuses on the project power of a somewhat lower number of recruits in the 4 cells: a,b,c, and d, with a current projection of about 100 per cell (children) as contrasted with the prior expectations of 150 per cell . The design has also changed in that we now have more sites (expected 36 sites, with 18 in S+ and 18 in S-, the school-level intervention and controls.

As before, we could not rely on commercial power programs because of the complexity of the design – a convention randomization scheme crossed with a cluster randomized trial (school intervention). For that reason, we again report the results of simulation of the expected power given a stipulated "effect size" for the treatments (A+ and S+) at the child and school levels.

Another reason for the simulations is that we are interested in particular contrasts of interest in the effects of S+ and A+

Reviewing the prior assumptions from 2016 design.

Earlier power calcs were done by ARL by simulation, which posed a 2 by 2 factorial design with A=patient level and S=school level intervention. Estimates assumed a continuous outcome (such as asthma control) and improvement over time (to simplify the longitudinal data design).

We assumed factorial terms of A and S main effects and A*S interaction.
The main effects of A and S take into account that any interaction will affect the main effects. If the interaction is synergistic, then the "main effects" will be larger than just the A term or the S term alone. Main effects in this context are the marginal effects (of A and S), and these marginal effects are larger if there is a synergistic interaction (if the interaction is negative, then of course, the marginal effects are smaller).

We assume a design effect for the S+ S- effect (cluster randomized, and now 36 clusters (18+ 18)). If we assume effect size variation across sites (schools) of 0.0 to 0.5, then sd of random intercept is about 0.125. That would mean that if sigmasq= 1.0, then sigmasq(b) = 0.125*0.125. =0.016. If we assume that the variation in effect size is broader (0 to 1.0), then sd= 0.25 and sigmasq(b) = 0.25* 0.25 = 0.0625. We used this broader assumption. Then ICC = 0.0625/(1.0625) = 0.06 and DEFF= 1+0.06*11 (assuming cluster size = 12) = 1+0.66= 1.66.

Plan is as follows.

Using program from Oct 2016, recreate power with 36 sites and 12 patients per site (n=432 overall). Results appear in the Table below.

Power for contrasts of interest. Effect sizes are in terms of Cohen's d nomenclature and represents the fraction of standard deviations of change over time.

Contrast	Contrast (Table cell)	Stipulated Effect size(sd)			Power (n=600)	Power (n=432)
		A	S	A,S		
S+ v S-	a,b vs c,d	0.35	0.5	0.0	0.96	0.97
A+ v A-	a,c vs b,d	0.35	0.5	0.0	0.99	0.95
S+A+ v S-A+	a vs c	0.35	0.5	0.0	0.89	0.88
S+A+ v S+A-	a vs b	0.35	0.5	0.0	0.87	0.75
S- A+ v S-A-	c vs d	0.35	0.5	0.0	0.85	0.71
S+,A- v S-A-	b vs d	0.35	0.5	0.0	0.89	0.83
		A	S	A,S		
S+ v S-	a,b vs c,d	0.35	0.5	0.25	0.99	0.99
A+ v A-	a,c vs b,d	0.35	0.5	0.25	0.99	0.99
S+A+ v S-A+	a vs c	0.35	0.5	0.25	0.99	0.99
S+A+ v S+A-	a vs b	0.35	0.5	0.25	0.99	0.99
S- A+ v S-A-	c vs d	0.35	0.5	0.25	0.85	0.71
S+,A- v S-A-	b vs d	0.35	0.5	0.25	0.89	0.83

A=Navigator effect; S=School Effect; AS= interaction; “+” = , “-“ = control
Cells e, f are not included in this table for simplicity and to be conservative.

Power to detect main effects

Power remains at acceptable levels for estimating the main effects and for some of the contrasts. Power is reduced, as one would expect, for contrasts that depend on the number of children enrolled.   Power is not changed for the contrasts of the school effect, likely because of the increase in the number of schools enrolled and randomized. If there is an interaction (synergy) of School and Child interventions, power to show main effects (school or child interventions) will increase as will power in which the S+A+ cell is one of the contrasted cells.

*Power to detect school*clinical synergy*

Power is diminished and does not approach conventional levels to demonstrate an interaction (a formal statistical tests of synergy) of school and child interventions, unless the synergy is substantial (not show), but that contrast might not be of clinical interest.

Sparse data considerations.

In addition, power will be reduced if some schools enroll insufficient children to be able to make a contrast within school over time or across child treatment groups, or if a school enrolls no one.

9. Ancillary and unresolved issues

Our overall goal is estimation of the effects of the intervention, and the quantification of mediation, where applicable.   To address conventional requests for tests of statistical significance, we will need to plan for statistical testing.

9.1 Multiple comparisons

Adjustments in critical levels for determining “statistical significance” when requested will follow these principles.

(a) If the contrast of interest is a primary or secondary outcome and pre-specified as such, we will make no adjustment

(b) All ad hoc, post SAP analyses and contrasts will be exploratory and hypothesis generating.

(c) All analyses for effect modification and mediation will consider the number of comparisons and make adjustments using simple Bonferroni correction for critical p-values.

9.2 Statistical power

It is very likely that all attempts to demonstrate heterogeneity of treatment effect and/or mediation will fail owing to inadequate numbers of patients spread over a large number of schools.

10. Update to Data Analysis Plan: Impact of Pandemic Restrictions. *<u>Presented to and approved by Data Safety Monitoring Board (DSMB) on 6/29/2021:</u>*

The original statistical analysis plan (SAP) includes the fitting of longitudinal models to evaluate the impact of study interventions on primary and secondary outcomes in models that model time as months from randomization, with splines (knots at 0, 3, 6, 9 and 12 months) and time by treatment interaction terms. Mixed-effects and population-averaged (GEE) models will be fitted.

The initial analyses will be conducted as planned. **Sensitivity analyses** will then be performed to evaluate the impact of pandemic restrictions (pre and post*, where *Post = Time Period after March 14, 2020).

Longitudinal models for the main analyses will be modified to include an indicator variable for post COVID-19 phase* with time by post Covid-19 interaction and time by post COVID-19 by intervention interaction terms. In these models, post Covid-19 phase will be included as a time varying covariate within individuals.

Post pandemic restrictions by time by intervention interaction terms: If significant, the impact of an intervention at a particular time point (e.g., at 3 months post-randomization) depends on post pandemic status (relative to pre).
Post pandemic restrictions by time terms: If significant, the expected value of the outcome at a particular time point depends on post pandemic status (relative to pre).

Post pandemic restrictions indicator variable: If significant, the expected value of the outcome variables depends on post pandemic status (relative to pre).

References:

Alosh M, Huque MF, Bretz F, D'Agostino RB S. Tutorial on statistical considerations on subgroup analysis in confirmatory clinical trials. Stat Med. 2017; 36(8):1334-60. [PMID: 27891631].

Altman DG, Moher D, Schulz KF. Improving the reporting of randomised trials: the CONSORT Statement and beyond. Stat Med. 2012; 31(25):2985-97. [PMID: 22903776]

Berger VW. Pros and cons of permutation tests in clinical trials. Stat Med. 2000; 19(10):1319-28. [PMID: 10814980]

Bind MAC, VanderWeele TJ, Coull BA, Schwartz JD. Causal mediation analysis for longitudinal data with exogenous exposure. Biostatistics. 2016. 17:122-134.

Campbell MK, Piaggio G, Elbourne DR, Altman DG. Consort 2010 statement: extension to cluster randomised trials. BMJ. 2012; 345

Carter BR, Hood K. Balance algorithm for cluster randomized trials. BMC Med Res Methodol. 2008; 8:65,2288-8-65. [PMID: 18844993]

Chakraborty B, Collins LM, Strecher VJ, Murphy SA. Developing multicomponent interventions using fractional factorial designs. Stat Med. 2009; 28(21):2687-708. [PMID: 19575485]

Collins LM, Dziak JJ, Kugler KC, Trail JB. Factorial experiments: efficient tools for evaluation of intervention components. Am J Prev Med. 2014; 47(4):498-504. [PMID: 25092122]

Couronne MR. Vidailhet JC, Corvol S, Lehéricy S, Durrleman D. Learning Disease Progression Models with Longitudinal Data and Missing Values. ISBI, April 2019

Daniel RM, De Stavola BL, Cousens SN. Gformula: Estimating causal effects in the presence of time-varying confounding or mediation using the g-computation formula. The Stata Journal. 2011. 11:479-517.

Ding P, Vanderweele TJ. Sharp sensitivity bounds for mediation under unmeasured mediator-outcome confounding. Biometrika. 2016; 103(2):483-90. [PMID: 27279672]

Ding P, VanderWeele TJ. Sensitivity Analysis Without Assumptions. Epidemiology. 2016; 27(3):368-77. [PMID: 26841057]

Elbourne DR, Campbell MK. Extending the CONSORT statement to cluster randomized trials: for discussion. Stat Med. 2001; 20(3):489-96. [PMID: 11180315]

Fay MP, Graubard B. (2001). Small-Sample Adjustments for Wald-Type Tests Using Sandwich Estimators. Biometrics . 2001. 57: 1198-1206.

Fay MP. Title Small-Sample Adjustments for Wald tests Using Sandwich Estimators Version 0.9-6.1 Date 2013-12-11.

Fitzmaurice GM, Laird NM, Ware JH. Applied Longitudinal Analysis. Second Edition. New York: Wiley; 2011

Gallis JA, Li F, Turner EL, XTGEEBCV: Stata module to compute bias-corrected (small-sample) standard errors for generalized estimating equations. Chestnut Hill. MA, Statistical Software Components S458623, Boston College Department of Economics; 2019.

Gelman A, Hill J. Data Analysis Using Regression and Multilevel/Hierarchical Models. Cambridge: Cambridge University Press; 2007

Good P. *Permutation, Parametric, and Bootstrap Tests of Hypotheses*. Third ed. New York: Springer-Verlag; 2005.

Greifer, N. (2019). cobalt: Covariate Balance Tables and Plots. R package version 3.7.0.

Hayes RJ, Moulton LH. Cluster Randomized Trials. Second Edition. Boca Raton, FL: Chapman and Hall/CRC; 2017. ISBN: 978-1-58488-816-1

Hicks R, Tingley D. Causal mediation analysis. The Stata Journal. 2011. 11:605-619.
Imai K, Keele L, Yamamoto T. Identification, Inference and Sensitivity Analysis for Causal Mediation Effects. Statistical Science. 2010. 25(1):51-71.

Imai K, Keele L, Tingley D. A general approach to causal mediation analysis. Psychological Methods. 2010. 15:309-334.

Ivers NM, Halperin IJ, Barnsley J, Grimshaw JM, Shah BR, Tu K, et al. Allocation techniques for balance at baseline in cluster randomized trials: a methodological review. Trials. 2012; 13:120,6215-13-120. [PMID: 22853820]

Kenny DA. PowMedR. http://www.davidakenny.net/progs/PowMedR.txt. Accessed July 24, 2016.

Lanza ST, Rhoades BL. Latent class analysis: an alternative perspective on subgroup analysis in prevention and treatment. Prev Sci. 2013 Apr;14(2):157-68. doi: 10.1007/s11121-011-0201-1. PubMed PMID: 21318625; PubMed Central PMCID: PMC3173585.

Lee OE, Braun TM. Permutation tests for random effects in linear mixed models. Biometrics. 2012; 68(2):486-93. [PMID: 21950470]

Little RJA. Regression with missing X's: a review. J Am Stat Assn. 1992;87:1227-37.

Little RJA, Rubin DB. Statistical Analysis with Missing Data. Second Edition. New York: John Wiley & Sons; 2002
Lynch KG, Cary M, Gallop R, Ten Have TR. Causal mediation analyses for randomized trials. Health Services Outcomes Res Method. 2008. 8:57-76.

MacKinnon DP, Fairchild AJ, Fritz JS. Mediation analysis. Annu Rev Psychol. 2007. 58:593-614.

Molenberghs, G, Kenward MG. Missing Data in Clinical Studies. New York: John Wiley & Sons; 2007

Moulton LH. Covariate-based constrained randomization of group-randomized trials. Clin Trials. 2004; 1(3):297-305. [PMID: 16279255]

Nietert PJ, Jenkins RG, Nemeth LS, Ornstein SM. An application of a modified constrained randomization process to a practice-based cluster randomized trial to improve colorectal cancer screening. Contemp Clin Trials. 2009; 30(2):129-32. [PMID: 18977314]

Nguyen JM, Holbrook JT, Wei CY, Gerald LB, Teague WG, Wise RA, et al. Validation and psychometric properties of the Asthma Control Questionnaire among children. J Allergy Clin Immunol. 2014; 133(1):91,7.e1-6. [PMID: 23932458]

Peters TJ, Richards SH, Bankhead CR, Ades AE, Sterne JA. Comparison of methods for analysing cluster randomized trials: an example involving a factorial design. Int J Epidemiol. 2003; 32(5):840-6. [PMID: 14559762]

Petersen ML, Sinisi SE, van der Laan MJ. Estimation of direct causal effects. Epidemiology. 2006. 17:276-284.

Preacher KJ. Advances in mediation analysis: a survey and synthesis of new developments. Annu Rev Psychol. 2015; 66:825-52. [PMID: 25148853]

Preacher KJ, Kelley K. Effect size measures for mediation models: quantitative strategies for communicating indirect effects. Psychol Methods. 2011; 16(2):93-115. [PMID: 21500915]

Rosenbaum PR. Covariance adjustment in randomized experiments and observational studies. *Statistical Science.* 2002;17:286-327.

Ryan P. Random allocation of treatments in blocks. Stata Technical Bulletin 54: 49–53; 50: 36–37; 49: 43–46. March 2000. Reprinted in Stata Technical Bulletin Reprints, vol. 9, pp. 353–358; vol. 9, pp. 352–353; vol. 7, pp. 297–300.

Shults, J., & Hilbe, J.M. (2014). Quasi-Least Squares Regression (1st ed.). Chapman and Hall/CRC. https://doi.org/10.1201/b16446

Sobel ME. What do randomized studies of housing mobility demonstrate? Causal inference in the face of interference. J Am Statist Assn. 2006.101:1398-1407. DOI 10.1198/01621-45060000000636.

Stapleton LM, Pituch K A. Dion E Standardized Effect Size Measures for Mediation Analysis in Cluster-Randomized Trials, The Journal of Experimental Education, 2015. 83:4, 547-582, DOI: 10.1080/00220973.2014.919569

Steen J, Loeys T, Moerkerke B, Vansteelandt S, Meys J, Lange T. Flexible mediation analysis using natural effect models. V. 0.6-0. 2015. https://gitbug.com/jmpsteen/medflex.

Talloen W, Moerkerke B, Loeys T, Naeghel JD, Van Keer H, Vansteelandt S. Estimation of indirect effects in the presence of unmeasured confounding for the mediator-outcome relationship in a multilevel 2-1-1 mediation model. J Educ & Behavioral Statistics. onlineFirst, March 30, 2016. Doi:10.2102/1076998616636855.

Tang L, Duan N, Klap R, Asarnow JR, Belin TR. Applying permutation tests with adjustment for covariates and attrition weights to randomized trials of health-services interventions. Stat Med. 2009; 28(1):65-74. [PMID: 18937226]

Ten Have TR, Joffe MM. A review of causal estimation of effects in mediation analyses. Stat Methods Med Res. 2012; 21(1):77-107. [PMID: 21163849]

Tingley D, Yamamoto J, Hirose K, Keele L, Imai K. mediation: R package for Causal Mediation Analysis. v 4.4.5. 2015. https://cran.r- project.org/web/packages/mediation/vignettes/mediation.pdf

Tingley D, Yamamoto T, Hirose K, Keele L, Imai K. mediation: R Package for Causal Mediation Analysis. Journal of Statistical Software. 2014. 59(5), 1-38. URL http://www.jstatsoft.org/v59/i05/

Valeri L, VanderWeele TJ. Mediation analysis allowing for exposure-mediator interactions and causal interpretation: theoretical assumptions and implementation with SAS and SPSS macros. *Psychological methods*. 2013;18(2):137-150. doi:10.1037/a0031034.

VanderWeele TJ, Vansteelandt S. Mediation Analysis with Multiple Mediators. Epidemiol Method. 2014; 2(1):95-115. [PMID: 25580377]

VanderWeele TJ. Direct and indirect effects for neighborhood-based clustered longitudinal data. Sociological Methods and Research. 2010. 38(4):5150544.
VanderWeele TJ. Mediation analysis: A practitioners guide. Annu Rev Public Health. 2016. 37:17-32.

VanderWeele TJ. Marginal structural models for the estimation of direct and indirect effects. Epidemiology. 2009; 20(1):18-26. [PMID: 19234398]

VanderWeele TJ. Explanation in Causal Inference. Methods for Mediation and Interaction. New York: Oxford University Press; 2015. ISBN 978-0-19-932587-0.

Vittinghoff E, Neilands T B. Sample Size for Joint Testing of Indirect Effects. Prevention Science. 2015. 16:1128—1135. doi=10.1007/s11121-014-0528-5.

Wang A, Arah OA. G-computation demonstration in causal mediation analysis. Eur J Epidemiol. 2013. 30:1119.1127. DOI 10.1007/s 10654-015-01

Zhang Z, Zhang G, Goyal H, Mo L, Hong Y. Identification of subclasses of sepsis that showed different clinical outcomes and responses to amount of fluid resuscitation: a latent profile analysis. *Crit Care*. 2018 Dec 18;22(1):347. doi: 10.1186/s13054-018-2279-3. PubMed PMID: 30563548; PubMed Central PMCID: PMC6299613.

Appendix: CONSORT Checklist for cluster randomized studies

The following two tables include items for reporting results from cluster randomized trials. With any update in the CONSORT checklist, this SAP will be amended.

Table 1: CONSORT 2010 checklist of information to include when reporting a cluster randomized trial

Section/Topic	Item No	Standard Checklist item	Extension for cluster designs	Page No *
**Title and abstract**				
	1a	Identification as a randomised trial in the title	Identification as a cluster randomised trial in the title	
	1b	Structured summary of trial design, methods, results, and conclusions (for specific guidance see CONSORT for abstracts)[i,ii]	See table 2	
**Introduction**				
**Background and objectives**	2a	Scientific background and explanation of rationale	Rationale for using a cluster design	
	2b	Specific objectives or hypotheses	Whether objectives pertain to the cluster level, the individual participant level or both	
**Methods**				
**Trial design**	3a	Description of trial design (such as parallel, factorial) including allocation ratio	Definition of cluster and description of how the design features apply to the clusters	
	3b	Important changes to methods after trial commencement (such as eligibility criteria), with reasons		
**Participants**	4a	Eligibility criteria for participants	Eligibility criteria for clusters	
	4b	Settings and locations where the data were collected		
**Interventions**	5	The interventions for each group with sufficient details to allow replication, including how and when they were actually administered	Whether interventions pertain to the cluster level, the individual participant level or both	
**Outcomes**	6a	Completely defined pre-specified primary and secondary outcome measures, including how and when they were assessed	Whether outcome measures pertain to the cluster level, the individual participant level or both	
	6b	Any changes to trial outcomes after the trial commenced, with reasons		
**Sample size**	7a	How sample size was determined	Method of calculation, number of clusters(s) (and whether equal or unequal cluster sizes are assumed), cluster size, a	

			coefficient of intracluster correlation (ICC or *k*), and an indication of its uncertainty	
	7b	When applicable, explanation of any interim analyses and stopping guidelines		
**Randomisation:**				
**Sequence generation**	8a	Method used to generate the random allocation sequence		
	8b	Type of randomisation; details of any restriction (such as blocking and block size)	Details of stratification or matching if used	
**Allocation concealment mechanism**	9	Mechanism used to implement the random allocation sequence (such as sequentially numbered containers), describing any steps taken to conceal the sequence until interventions were assigned	Specification that allocation was based on clusters rather than individuals and whether allocation concealment (if any) was at the cluster level, the individual participant level or both	
**Implementation**	10	Who generated the random allocation sequence, who enrolled participants, and who assigned participants to interventions	Replace by 10a, 10b and 10c	
	10a		Who generated the random allocation sequence, who enrolled clusters, and who assigned clusters to interventions	
	10b		Mechanism by which individual participants were included in clusters for the purposes of the trial (such as complete enumeration, random sampling)	
	10c		From whom consent was sought (representatives of the cluster, or individual cluster members, or both), and whether consent was sought before or after randomisation	
**Blinding**	11a	If done, who was blinded after assignment to interventions (for example, participants, care providers, those assessing outcomes) and how		
	11b	If relevant, description of the similarity of interventions		
**Statistical methods**	12a	Statistical methods used to compare groups for primary and secondary outcomes	How clustering was taken into account	

	12b	Methods for additional analyses, such as subgroup analyses and adjusted analyses	
**Results**			
**Participant flow (a diagram is strongly recommended)**	13a	For each group, the numbers of participants who were randomly assigned, received intended treatment, and were analysed for the primary outcome	For each group, the numbers of clusters that were randomly assigned, received intended treatment, and were analysed for the primary outcome
	13b	For each group, losses and exclusions after randomisation, together with reasons	For each group, losses and exclusions for both clusters and individual cluster members
**Recruitment**	14a	Dates defining the periods of recruitment and follow-up	
	14b	Why the trial ended or was stopped	
**Baseline data**	15	A table showing baseline demographic and clinical characteristics for each group	Baseline characteristics for the individual and cluster levels as applicable for each group
**Numbers analysed**	16	For each group, number of participants (denominator) included in each analysis and whether the analysis was by original assigned groups	For each group, number of clusters included in each analysis
**Outcomes and estimation**	17a	For each primary and secondary outcome, results for each group, and the estimated effect size and its precision (such as 95% confidence interval)	Results at the individual or cluster level as applicable and a coefficient of intracluster correlation (ICC or k) for each primary outcome
	17b	For binary outcomes, presentation of both absolute and relative effect sizes is recommended	
**Ancillary analyses**	18	Results of any other analyses performed, including subgroup analyses and adjusted analyses, distinguishing pre-specified from exploratory	
**Harms**	19	All important harms or unintended effects in each group (for specific guidance see CONSORT for harms[iii])	
**Discussion**			
**Limitations**	20	Trial limitations, addressing sources of potential bias, imprecision, and, if relevant, multiplicity of analyses	
**Generalisability**	21	Generalisability (external validity, applicability) of the trial findings	Generalisability to clusters and/or individual participants (as relevant)

**Interpretation**	22	Interpretation consistent with results, balancing benefits and harms, and considering other relevant evidence		
**Other information**				
**Registration**	23	Registration number and name of trial registry		
**Protocol**	24	Where the full trial protocol can be accessed, if available		
**Funding**	25	Sources of funding and other support (such as supply of drugs), role of funders		

** Note: page numbers optional depending on journal requirements*

Table 2: Extension of CONSORT for abstracts to reports of cluster randomized trials

Item	Standard Checklist item	Extension for cluster trials
**Title**	Identification of study as randomised	**Identification of study as cluster randomised**
**Trial design**	Description of the trial design (e.g., parallel, cluster, non-inferiority)	
**Methods**		
**Participants**	Eligibility criteria for participants and the settings where the data were collected	**Eligibility criteria for clusters**
**Interventions**	Interventions intended for each group	
**Objective**	Specific objective or hypothesis	**Whether objective or hypothesis pertains to the cluster level, the individual participant level or both**
**Outcome**	Clearly defined primary outcome for this report	**Whether the primary outcome pertains to the cluster level, the individual participant level or both**
**Randomization**	How participants were allocated to interventions	**How clusters were allocated to interventions**
**Blinding (masking)**	Whether or not participants, care givers, and those assessing the outcomes were blinded to group assignment	
**Results**		
**Numbers randomized**	Number of participants randomized to each group	**Number of clusters randomized to each group**
**Recruitment**	Trial status[1]	
**Numbers analysed**	Number of participants analysed in each group	**Number of clusters analysed in each group**
**Outcome**	For the primary outcome, a result for each group and the estimated effect size and its precision	**Results at the cluster or individual participant level as applicable for each primary outcome**
**Harms**	Important adverse events or side effects	
**Conclusions**	General interpretation of the results	
**Trial registration**	Registration number and name of trial register	
**Funding**	Source of funding	

[1] Relevant to Conference Abstracts

REFERENCES (to CONSORT 2010)

[i] Hopewell S, Clarke M, Moher D, Wager E, Middleton P, Altman DG, et al. CONSORT for reporting randomised trials in journal and conference abstracts. *Lancet* 2008, 371:281-283

[ii] Hopewell S, Clarke M, Moher D, Wager E, Middleton P, Altman DG at al (2008) CONSORT for reporting randomized controlled trials in journal and conference abstracts: explanation and elaboration. *PLoS Med* 5(1): e20

[iii] Ioannidis JP, Evans SJ, Gotzsche PC, O'Neill RT, Altman DG, Schulz K, Moher D. Better reporting of harms in randomized trials: an extension of the CONSORT statement. *Ann Intern Med* 2004; 141(10):781-788.
