# Supplementary material for: Community Health Workers Linking Clinics and Schools and Asthma Control: A Randomized Clinical Trial
Source: JAMA Pediatr. 2024 Oct 21;178(12):1260–9. doi: 10.1001/jamapediatrics.2024.3967 (PMC11581744; doi:10.1001/jamapediatrics.2024.3967)
Supplement: Supplement 3. — eFigure 1. Graphs of Fitted Values of Asthma Control vs Planned Month of Visit eFigure 2. Graph of Fitted Values of Daytime Symptoms in Past 2 Weeks vs Planned Month of Visit eFigure 3. Graph of Fitted Values of Nighttime Symptoms in Past 2 Weeks vs Planned Month of Visit eFigure 4. Graphs of Fitted Values of Number ED Visits in Previous 365 Days vs Planned Month of Visit eFigure 5. Graphs of Fitted Values of Number Hospital (HOSP) Visits in Previous 365 days vs Planned Month of Visit eFigure 6. Graphs of Fitted Values of Quality of Life (Emotional) vs Planned Month of Visit eFigure 7. Graphs of Fitted Values of Quality of Life (Activity) vs Planned Month of Visit eFigure 8. Graphs of Fitted Values of Number Steroid Courses in Previous 365 days vs Planned Month of Visit eFigure 9. Graph of Fitted Values of Daytime Symptoms in Past 2 weeks vs Planned Month of Visit eFigure 10. Graph of Fitted Values of Nighttime Symptoms in Past 2 weeks vs Planned Month of Visit eFigure 11. Graphs of Fitted Values of Number ED Visits in Previous 365 days vs Planned Month of Visit eFigure 12. Graphs of Fitted Values of Number HOSP Visits in Previous 365 days vs Planned Month of Visit eFigure 13. Graphs of Fitted Values of Asthma Control vs Planned Month of Visit eFigure 14. Graphs of Fitted Values of Quality of Life (Activity) vs Planned Month of Visit eFigure 15. Graphs of Fitted Values of Number Steroid Courses in Previous 365 days vs Planned Month of Visit eTable 1. Fitted Values for Secondary Outcomes at Baseline and 12 Months eTable 2. Fitted Values of Secondary Outcomes at 12 Months, Stratified by Study Completion Before the COVID-19 Pandemic Phase [file jamapediatr-e243967-s003.pdf]

Supplementary Online Content

Bryant-Stephens T, Kenyon CC, Tingey C, et al. Community health workers linking clinics and schools and asthma control: a randomized clinical trial. *JAMA Pediatr*. Published online October 21, 2024. doi:10.1001/jamapediatrics.2024.3967

eFigure 1. Graphs of Fitted Values of Asthma Control vs Planned Month of Visit

eFigure 2. Graph of Fitted Values of Daytime Symptoms in Past 2 Weeks vs Planned Month of Visit

eFigure 3. Graph of Fitted Values of Nighttime Symptoms in Past 2 Weeks vs Planned Month of Visit

eFigure 4. Graphs of Fitted Values of Number ED Visits in Previous 365 Days vs Planned Month of Visit

eFigure 5. Graphs of Fitted Values of Number Hospital (HOSP) Visits in Previous 365 days vs Planned Month of Visit

eFigure 6. Graphs of Fitted Values of Quality of Life (Emotional) vs Planned Month of Visit

eFigure 7. Graphs of Fitted Values of Quality of Life (Activity) vs Planned Month of Visit

eFigure 8. Graphs of Fitted Values of Number Steroid Courses in Previous 365 days vs Planned Month of Visit

eFigure 9. Graph of Fitted Values of Daytime Symptoms in Past 2 weeks vs Planned Month of Visit

eFigure 10. Graph of Fitted Values of Nighttime Symptoms in Past 2 weeks vs Planned Month of Visit

eFigure 11. Graphs of Fitted Values of Number ED Visits in Previous 365 days vs Planned Month of Visit

eFigure 12. Graphs of Fitted Values of Number HOSP Visits in Previous 365 days vs Planned Month of Visit

eFigure 13. Graphs of Fitted Values of Asthma Control vs Planned Month of Visit

eFigure 14. Graphs of Fitted Values of Quality of Life (Activity) vs Planned Month of Visit

eFigure 15. Graphs of Fitted Values of Number Steroid Courses in Previous 365 days vs Planned Month of Visit

eTable 1. Fitted Values for Secondary Outcomes at Baseline and 12 Months

eTable 2. Fitted Values of Secondary Outcomes at 12 Months, Stratified by Study Completion Before the COVID-19 Pandemic Phase

This supplementary material has been provided by the authors to give readers additional information about their work.

© 2024 Bryant-Stephens T et al. *JAMA Pediatrics*.

Section One. Figure of fitted values to accompany Table 2 of main manuscript.

eFigure 1. Graphs of fitted Values of Asthma Control versus planned month of visit. Fitted values are from a linear mixed-effects model with random intercepts for school and child. The models included time (indicator variables for planned month of visit), indicator variables for each intervention, and time by intervention interaction terms. For Participants with all visits prior to shutdown the number of participants in each treatment group = 23 for S+P+; 14 for S+Alone; 37 for P+Alone; 40 for Control. For Participants with some visits prior to shutdown and some visits after shutdown the number of participants in each treatment group = 64 for S+P+; 74 for S+Alone; 62 for P+Alone; 75 for Control. For Participants with all visits after shutdown the number of participants in each treatment group = 33 for S+P+; 24 for S+Alone; 90 for P+Alone; 86 for Control. This analysis excluded 1 measurement taken after 800 days of follow-up.

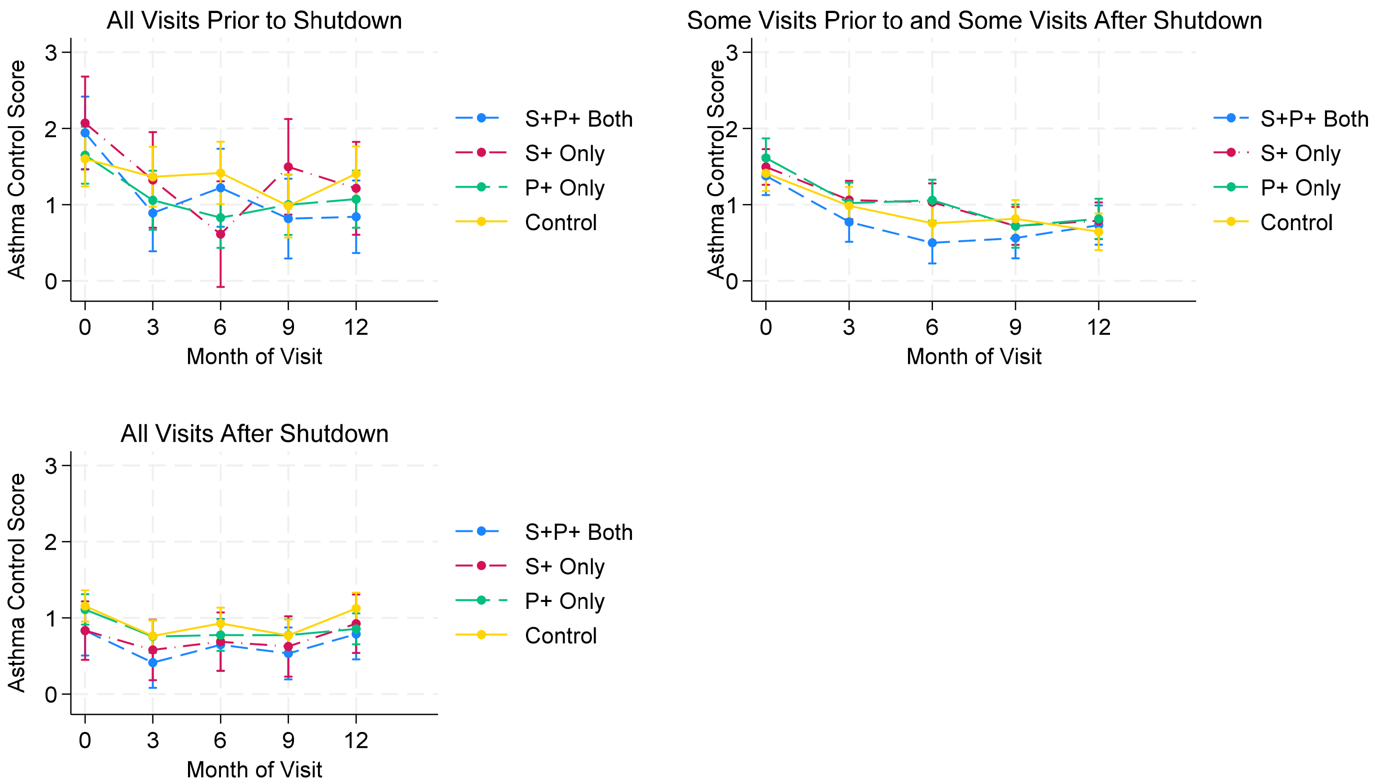

Section Two. Figures of fitted values to accompany Table 3 of main manuscript.

For the description of sample sizes in this section, within each treatment group define: nboth = the number of participants who have measurements at both baseline and 12 months; n0only = the number of participants who have measurements at baseline only; and n12only = the number of participants who have measurements at 12 months only.

© 2024 Bryant-Stephens T et al. *JAMA Pediatrics*.

eFigure 2. Graph of fitted Values of Daytime Symptoms in past 2 weeks versus planned month of visit. Fitted values are from a mixed-effects generalized binomial model (n=14) with random intercepts for child. (The model with random intercepts for both child and school did not converge.) The models included time (day of follow-up),indicator variables for each intervention, and time by intervention interaction terms. Number of participants in each treatment group = 120 for S+P+ (of which nboth = 115, n0only = 3, n12only = 2); 111 for S+Alone (of which nboth = 99, n0only = 7, n12only = 5); 188 for P+Alone (of which nboth = 175, n0only = 7, n12only = 6); 199 for Control (of which nboth = 188, n0only = 5, n12only = 6). This analysis excluded 1 measurement taken after 800 days of follow-up. (Number of ED Visits was obtained from the electronic health record at day of follow up = 0 and 365 (Month 0 and 12, respectively) and was available for the participants who were lost to follow-up for the other outcomes at Month 12.)

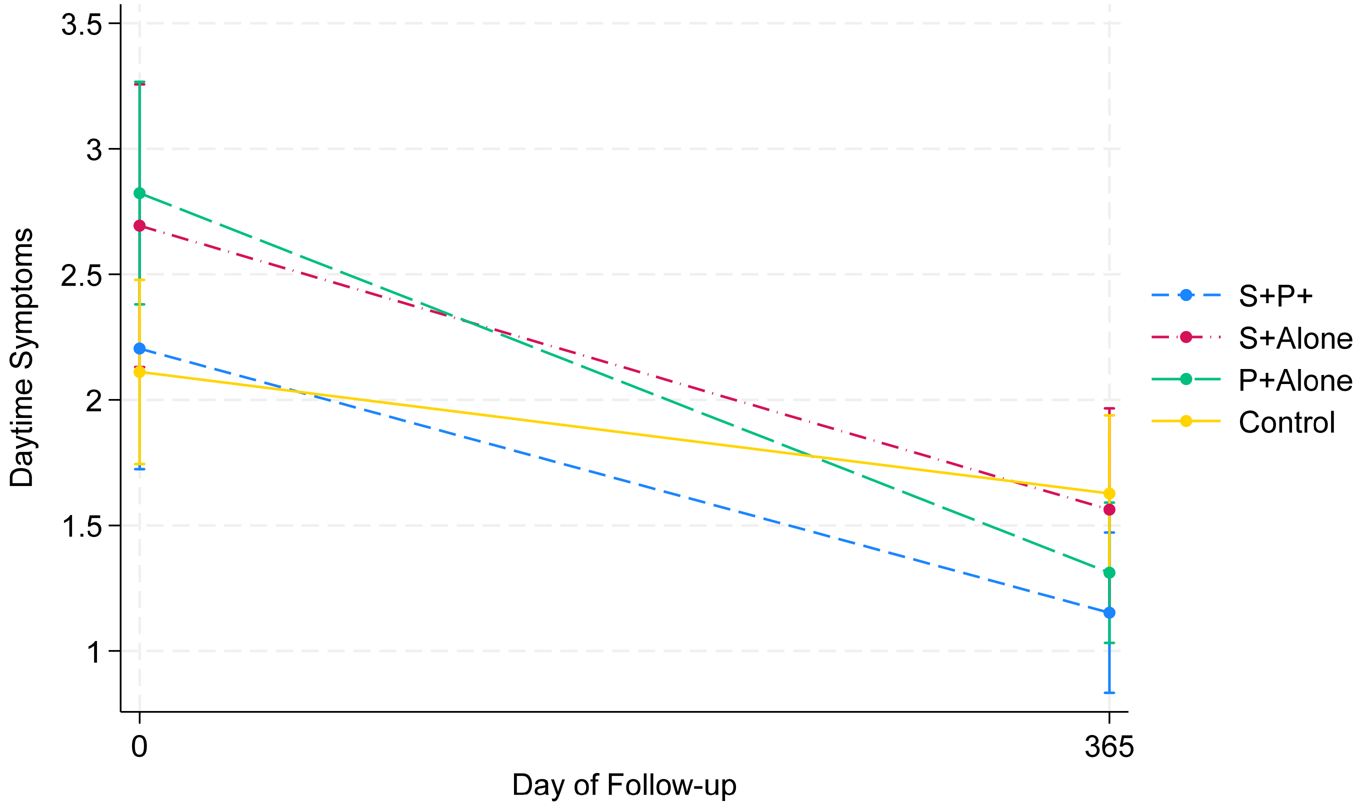

eFigure 3. Graph of fitted Values of Nighttime Symptoms in past 2 weeks versus planned month of visit. Fitted values are from a mixed-effects generalized binomial model (n=14) with random intercepts for child. (The model with random intercepts for both child and school did not converge.) The models included time (day of follow-up), indicator variables for each intervention, and time by intervention interaction terms. Number of participants in each treatment group = 120 for S+P+ (of which nboth = 114, n0only = 4, n12only = 2); 112 for S+Alone (of which nboth = 101, n0only = 6, n12only = 5); 189 for P+Alone (of which nboth = 172, n0only = 10, n12only = 7); 198 for Control (of which nboth = 189, n0only = 4, n12only = 5). This analysis excluded 1 measurement taken after 800 days of follow-up.

© 2024 Bryant-Stephens T et al. *JAMA Pediatrics*.

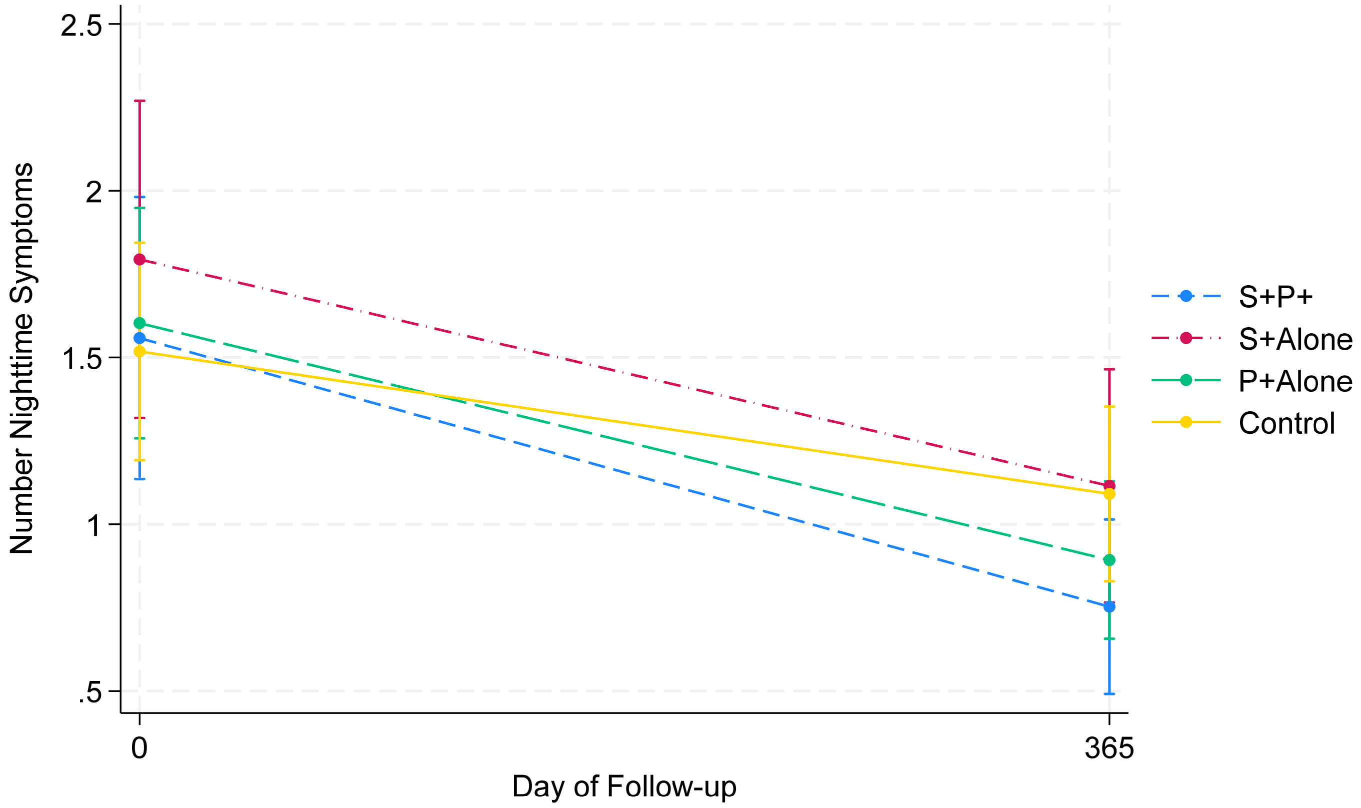

eFigure 4. Graphs of fitted Values of Number ED Visits in previous 365 days versus planned month of visit. Fitted values are from a mixed-effects binomial model (n=365) with random intercepts for child. (The model with random intercepts for both child and school did not converge.) The models included time (day of follow-up), indicator variables for each intervention, and time by intervention interaction terms. Number of participants in each treatment group = 121 for S+P+ (of which nboth = 121, n0only = 0, n12only = 0); 112 for S+Alone (of which nboth = 112, n0only = 0, n12only = 0); 192 for P+Alone (of which nboth = 191, n0only = 1, n12only = 0); 201 for Control (of which nboth = 201, n0only = 0, n12only = 0). The number of ED visits in the year prior is from EHR data and was measured at baseline and at day 365 (Month 12) on all participants, including those participants who did not have other outcomes measured at 12 months.

© 2024 Bryant-Stephens T et al. *JAMA Pediatrics*.

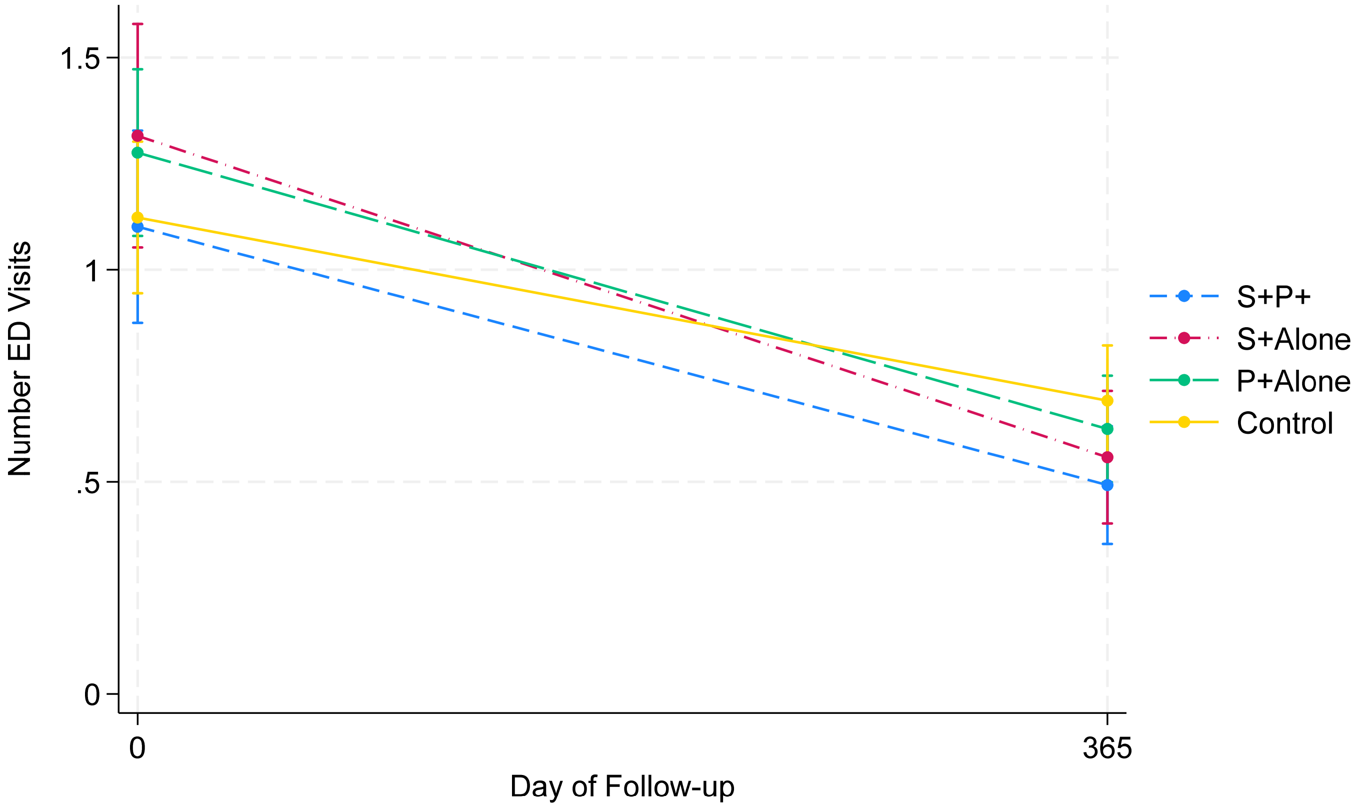

eFigure 5. Graphs of fitted Values of Number Hospital (HOSP) Visits in previous 365 days versus planned month of visit. Fitted values are from a mixed-effects binomial model (n=365) with random intercepts for child. (The model with random intercepts for both child and school did not converge.) The models included time (day of follow-up), indicator variables for each intervention, and time by intervention interaction terms. Number of participants in each treatment group = 121 for S+P+ (of which nboth = 121, n0only = 0, n12only = 0); 112 for S+Alone (of which nboth = 112, n0only = 0, n12only = 0); 192 for P+Alone (of which nboth = 192, n0only = 0, n12only = 0); 201 for Control (of which nboth = 201, n0only = 0, n12only = 0). The number of HOSP visits in the year prior is from EHR data and was measured at baseline and at day 365 (Month 12) on all participants, including those participants who did not have other outcomes measured at 12 months.

© 2024 Bryant-Stephens T et al. *JAMA Pediatrics*.

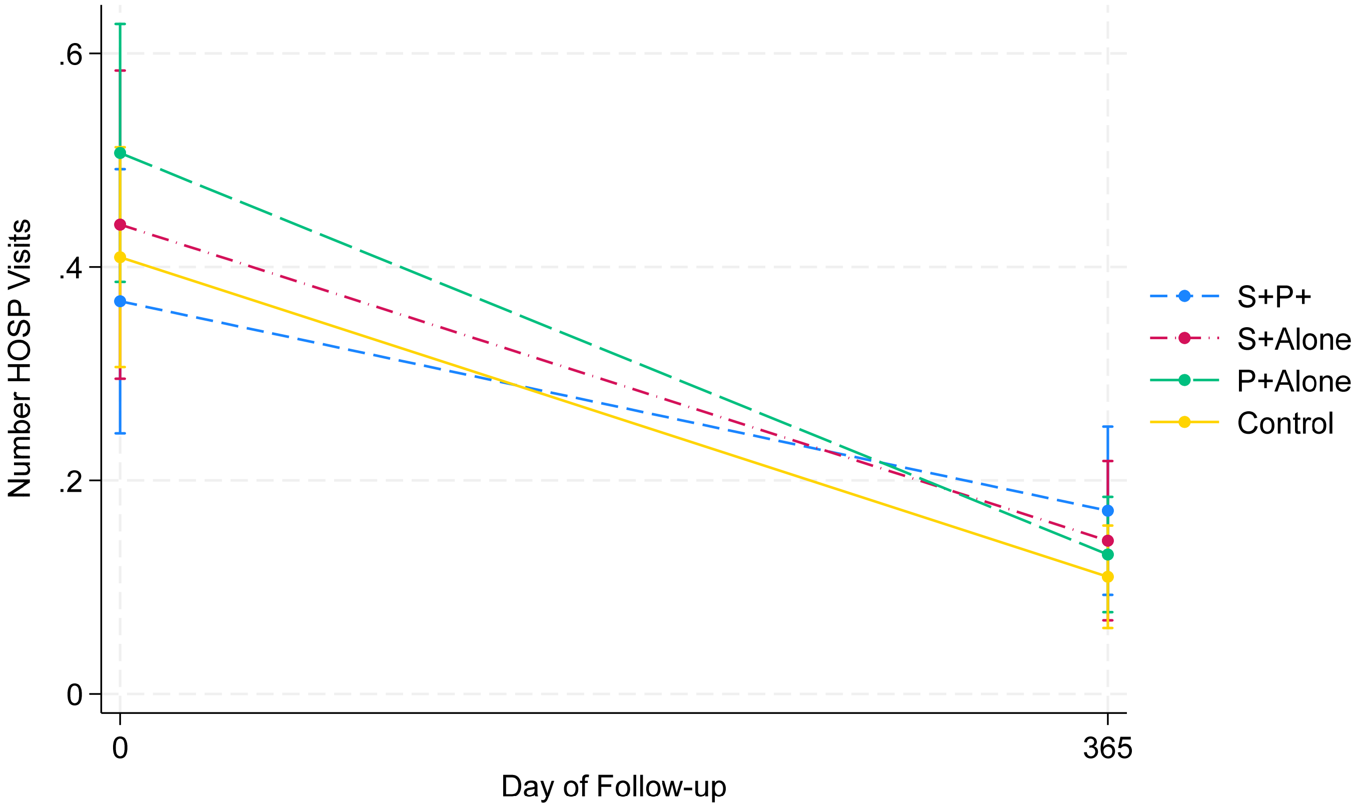

eFigure 6. Graphs of fitted Values of Quality of Life (Emotional) versus planned month of visit. Fitted values are from a linear mixed-effects model with random intercepts for school and child. The models included time (day of follow-up), indicator variables for each intervention, and time by intervention interaction terms. Number of participants in each treatment group = 120 for S+P+ (of which nboth = 117, n0only = 3, n12only = 0); 112 for S+Alone (of which nboth = 108, n0only = 4, n12only = 0); 188 for P+Alone (of which nboth = 182, n0only = 6, n12only = 0); 200 for Control (of which nboth = 193, n0only = 6, n12only = 1).

© 2024 Bryant-Stephens T et al. *JAMA Pediatrics*.

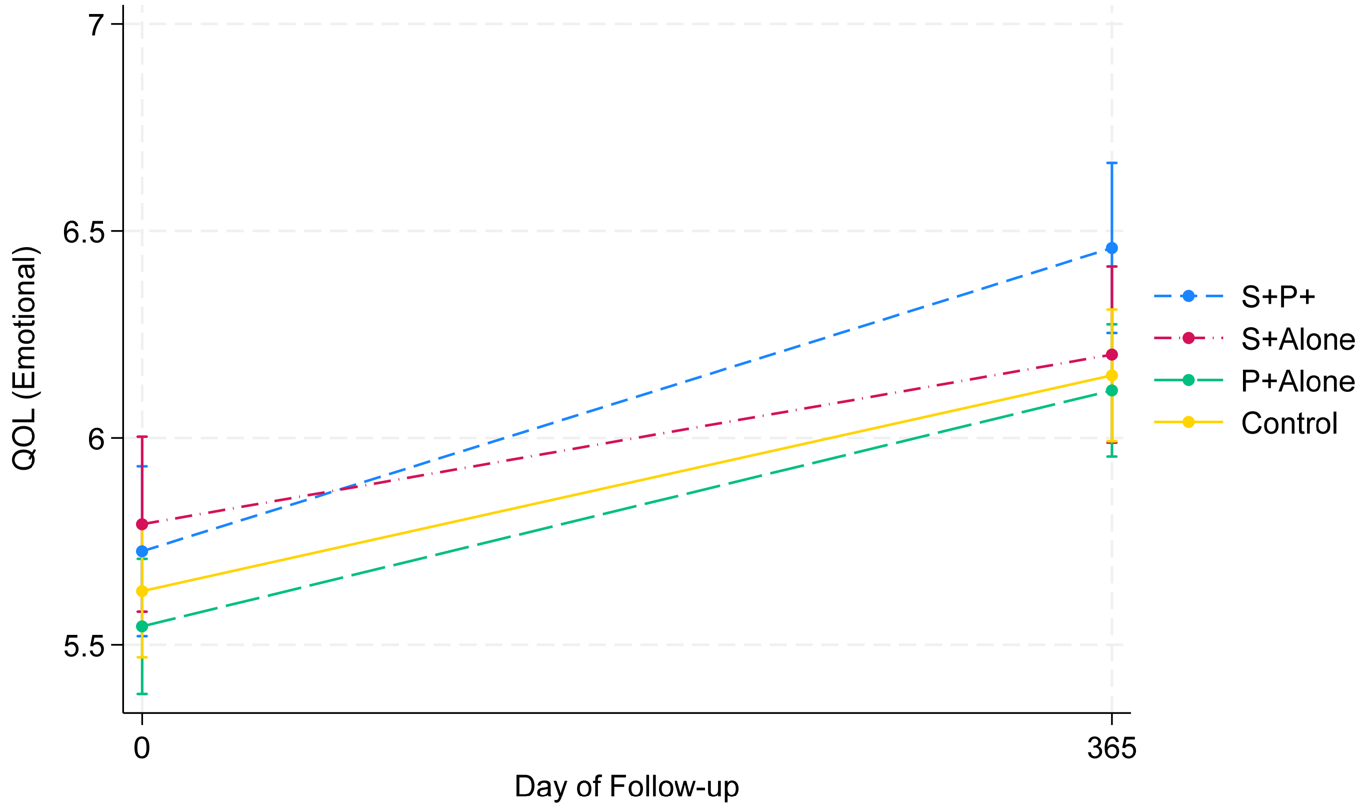

eFigure 7. Graphs of fitted Values of Quality of Life (Activity) versus planned month of visit. Fitted values are from a linear mixed-effects model with random intercepts for school and child. The models included time (day of follow-up), indicator variables for each intervention, and time by intervention interaction terms. Number of participants in each treatment group = 120 for S+P+ (of which nboth = 117, n0only = 3, n12only = 0); 112 for S+Alone (of which nboth = 108, n0only = 4, n12only = 0); 188 for P+Alone (of which nboth = 181, n0only = 7, n12only = 0); 200 for Control (of which nboth = 193, n0only = 6, n12only = 1).

© 2024 Bryant-Stephens T et al. *JAMA Pediatrics*.

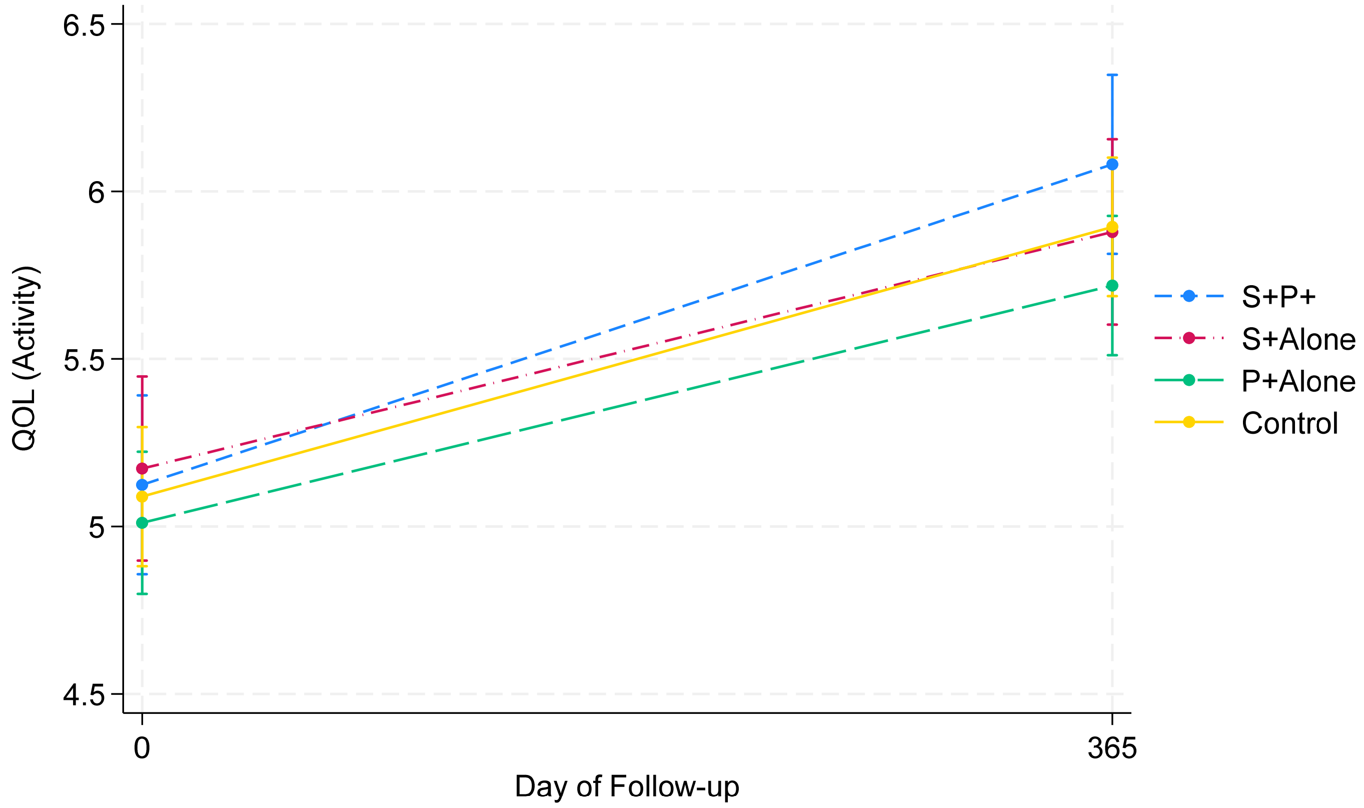

eFigure 8. Graphs of fitted Values of Number Steroid Courses in previous 365 days versus planned month of visit. Fitted values are from a mixed-effects Poisson model with random intercepts for child. (The generalized Poisson model with random intercepts for both child and school has AIC = 3230.5133 and BIC = 3281.8382. The generalized Poisson model with random intercepts for child only has AIC = 3230.8511 and BIC = 3277.0436. A generalized negative binomial models with random intercepts did not converge and the generalized negative binomial model with random intercepts for child and school had larger AIC and BIC values [AIC = 3232.5133 and BIC = 3288.9708]. We selected the generalized Poisson model with only child intercepts because it had the smallest BIC value, although it had slightly larger AIC than the generalized Poisson model with random intercepts for both child and school.) The models included time (day of follow-up), indicator variables for each intervention, and time by intervention interaction terms. Number of participants in each treatment group = 121 for S+P+ (of which nboth = 121, n0only = 0, n12only = 0); 112 for S+Alone (of which nboth = 112, n0only = 0, n12only = 0); 192 for P+Alone (of which nboth = 192, n0only = 0, n12only = 0); 201 for Control (of which nboth = 201, n0only = 0, n12only = 0). The number of Steroid visits in the year was available at baseline and at day 365 (Month 12) on all participants, including those participants who did not have other outcomes measured at 12 months.

© 2024 Bryant-Stephens T et al. *JAMA Pediatrics*.

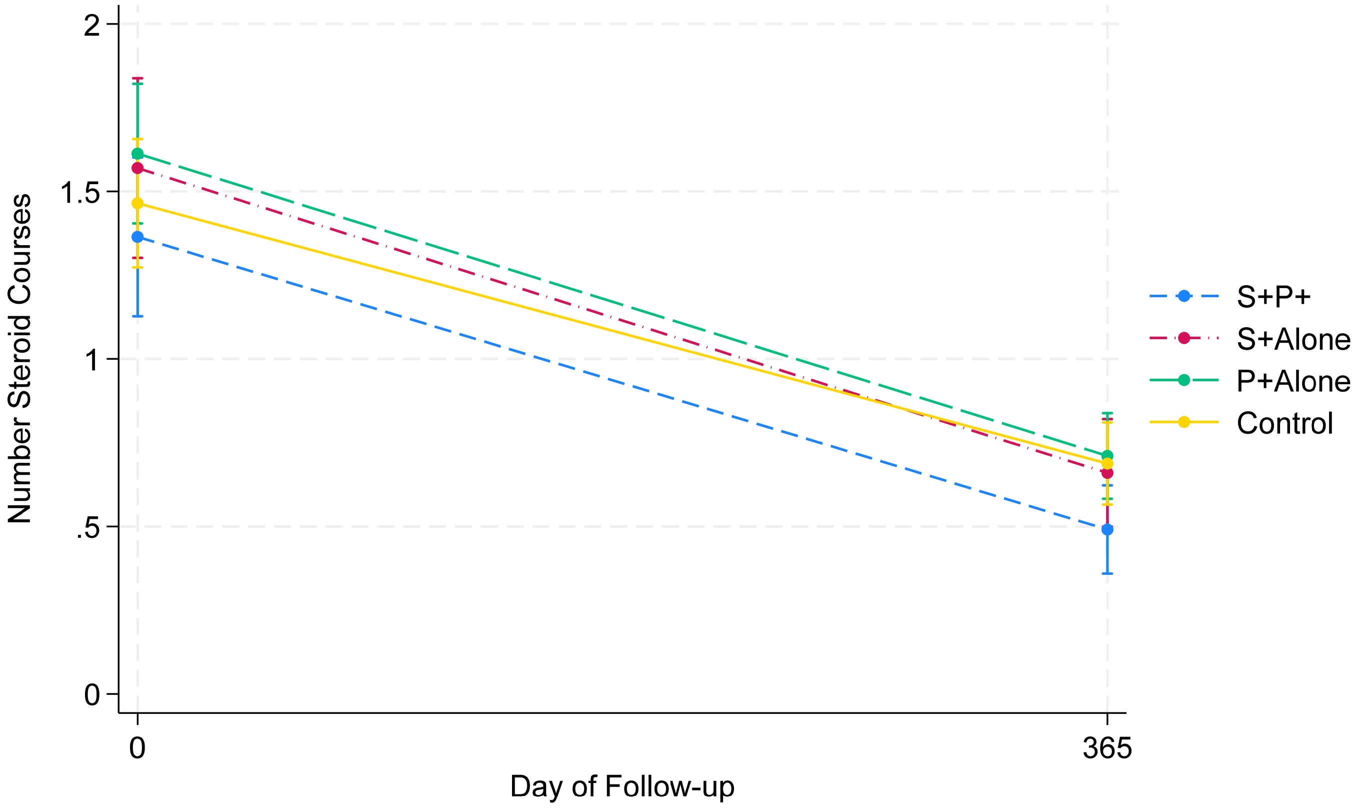

Section Two. Figures of fitted values to accompany Table 3 of main manuscript.

For the description of sample sizes in this section, within each treatment group define: nboth = the number of participants who have measurements at both baseline and 12 months; n0only = the number of participants who have measurements at baseline only; and n12only = the number of participants who have measurements at 12 months only.

eFigure 9. Graph of fitted Values of Daytime Symptoms in past 2 weeks versus planned month of visit. Fitted values are from a mixed-effects generalized binomial model (n=14) with random intercepts for child. (The model with random intercepts for both child and school did not converge.) The models included time (day of follow-up), indicator variables for each intervention, and time by intervention interaction terms. For Participants with all visits prior to shutdown: Number of participants in each treatment group = 23 for S+P+ (of which nboth = 23, n0only = 0, n12only = 0); 13 for S+Alone (of which nboth = 7, n0only = 3, n12only = 3); 36 for P+Alone (of which nboth = 33, n0only = 0, n12only = 3); 40 for Control (of which nboth = 35, n0only = 1, n12only = 4). For Participants with some visits prior to shutdown and some visits after shutdown: Number of participants in each treatment group = 63 for S+P+ (of which nboth = 59, n0only = 2, n12only = 2); 74 for S+Alone (of which nboth = 68, n0only = 4, n12only = 2); 62 for P+Alone (of which nboth = 55, n0only = 4, n12only = 3); 73 for Control (of which nboth = 68, n0only = 3, n12only = 2). For Participants with all visits after shutdown: Number of participants in each treatment group = 33 for S+P+ (of which nboth = 32, n0only = 1, n12only = 0); 24 for S+Alone (of which nboth = 24, n0only = 0, n12only = 0); 90 for P+Alone (of which nboth = 87,

© 2024 Bryant-Stephens T et al. *JAMA Pediatrics*.

n0only = 3, n12only = 0); 86 for Control (of which nboth = 85, n0only = 1, n12only = 0). This analysis excluded 1 measurement taken after 800 days of follow-up.

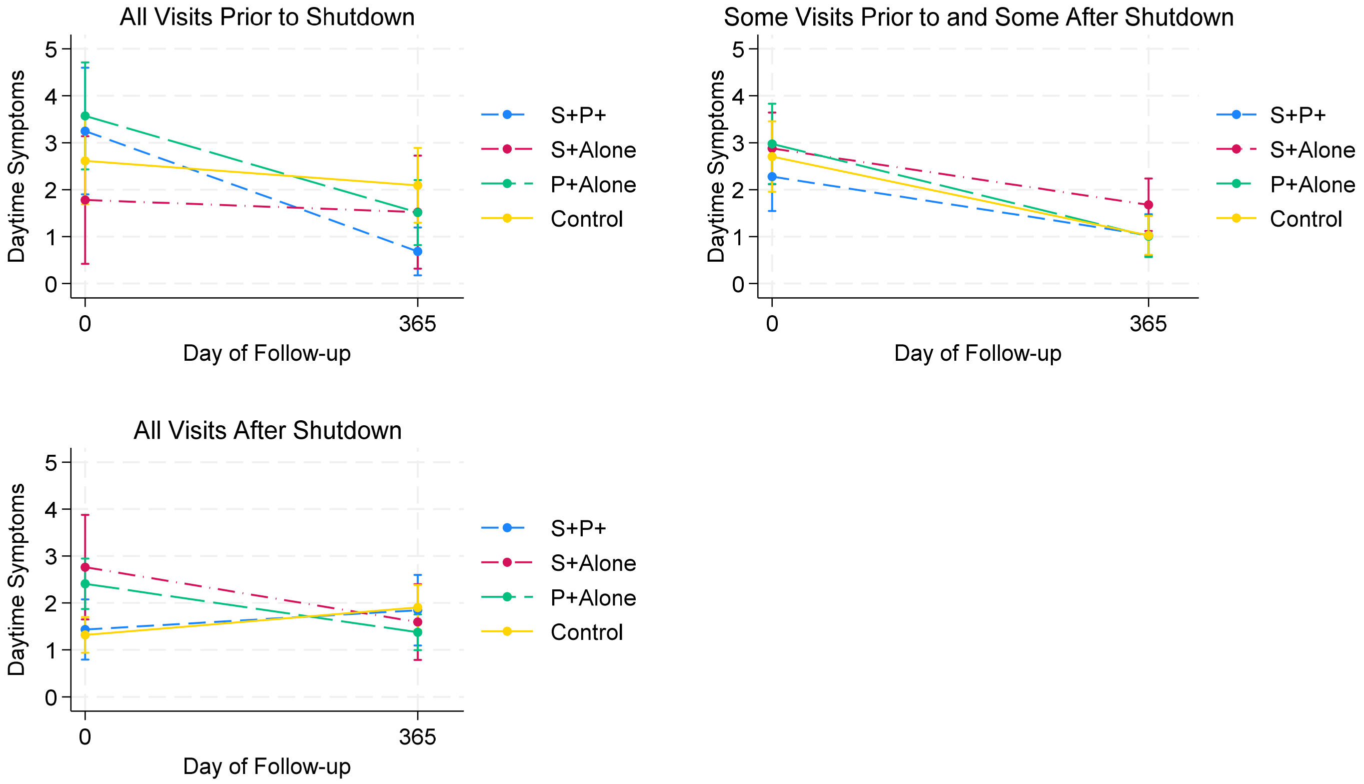

eFigure 10. Graph of fitted Values of Nighttime Symptoms in past 2 weeks versus planned month of visit. Fitted values are from a mixed-effects generalized binomial model (n=14) with random intercepts for child. (The model with random intercepts for both child and school did not converge.) The models included time (day of follow-up), indicator variables for each intervention, and time by intervention interaction terms. For Participants with all visits prior to shutdown: Number of participants in each treatment group = 23 for S+P+ (of which nboth = 21, n0only = 1, n12only = 1); 14 for S+Alone (of which nboth = 11, n0only = 1, n12only = 2); 37 for P+Alone (of which nboth = 30, n0only = 3, n12only = 4); 39 for Control (of which nboth = 37, n0only = 0, n12only = 2). For Participants with some visits prior to shutdown and some visits after shutdown: Number of participants in each treatment group = 63 for S+P+ (of which nboth = 60, n0only = 2, n12only = 1); 74 for S+Alone (of which nboth = 66, n0only = 5, n12only = 3); 62 for P+Alone (of which nboth = 56, n0only = 4, n12only = 2); 73 for Control (of which nboth = 67, n0only = 3, n12only = 3). For Participants with all visits after shutdown: Number of participants in each treatment group = 33 for S+P+ (of which nboth = 32, n0only = 1, n12only = 0); 24 for S+Alone (of which nboth = 24, n0only = 0, n12only = 0); 90 for P+Alone (of which nboth = 86, n0only = 3, n12only = 1); 86 for Control (of which nboth = 85, n0only = 1, n12only = 0). This analysis excluded 1 measurement taken after 800 days of follow-up.

© 2024 Bryant-Stephens T et al. *JAMA Pediatrics*.

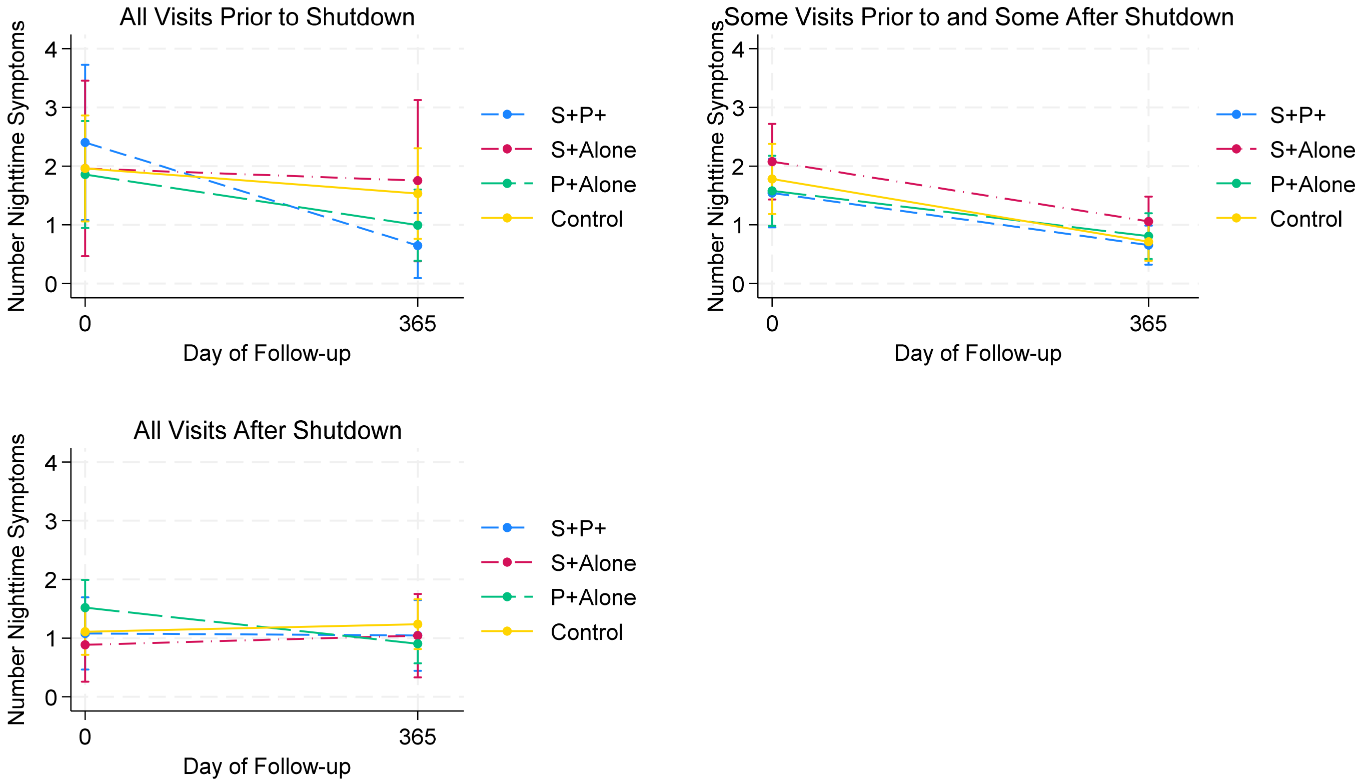

eFigure 11. Graphs of fitted Values of Number ED Visits in previous 365 days versus planned month of visit. Fitted values are from a mixed-effects binomial model (n=365) with random intercepts for child. (The model with random intercepts for both child and school did not converge.) The models included time (day of follow-up), indicator variables for each intervention, and time by intervention interaction terms. For Participants with all visits prior to shutdown: Number of participants in each treatment group = 23 for S+P+ (of which nboth = 23, n0only = 0, n12only = 0); 14 for S+Alone (of which nboth = 14, n0only = 0, n12only = 0); 37 for P+Alone (of which nboth = 37, n0only = 0, n12only = 0); 40 for Control (of which nboth = 40, n0only = 0, n12only = 0). For Participants with some visits prior to shutdown and some visits after shutdown: Number of participants in each treatment group = 64 for S+P+ (of which nboth = 63, n0only = 1, n12only = 0); 74 for S+Alone (of which nboth = 74, n0only = 0, n12only = 0); 62 for P+Alone (of which nboth = 61, n0only = 1, n12only = 0); 75 for Control (of which nboth = 72, n0only = 3, n12only = 0). For Participants with all visits after shutdown: Number of participants in each treatment group = 33 for S+P+ (of which nboth = 33, n0only = 0, n12only = 0); 24 for S+Alone (of which nboth = 24, n0only = 0, n12only = 0); 90 for P+Alone (of which nboth = 90, n0only = 0, n12only = 0); 86 for Control (of which nboth = 86, n0only = 0, n12only = 0). The number of ED visits in the year prior is from EHR data and was measured at baseline and at day 365 (Month 12) on all participants, including those participants who did not have other outcomes measured at 12 months.

© 2024 Bryant-Stephens T et al. *JAMA Pediatrics*.

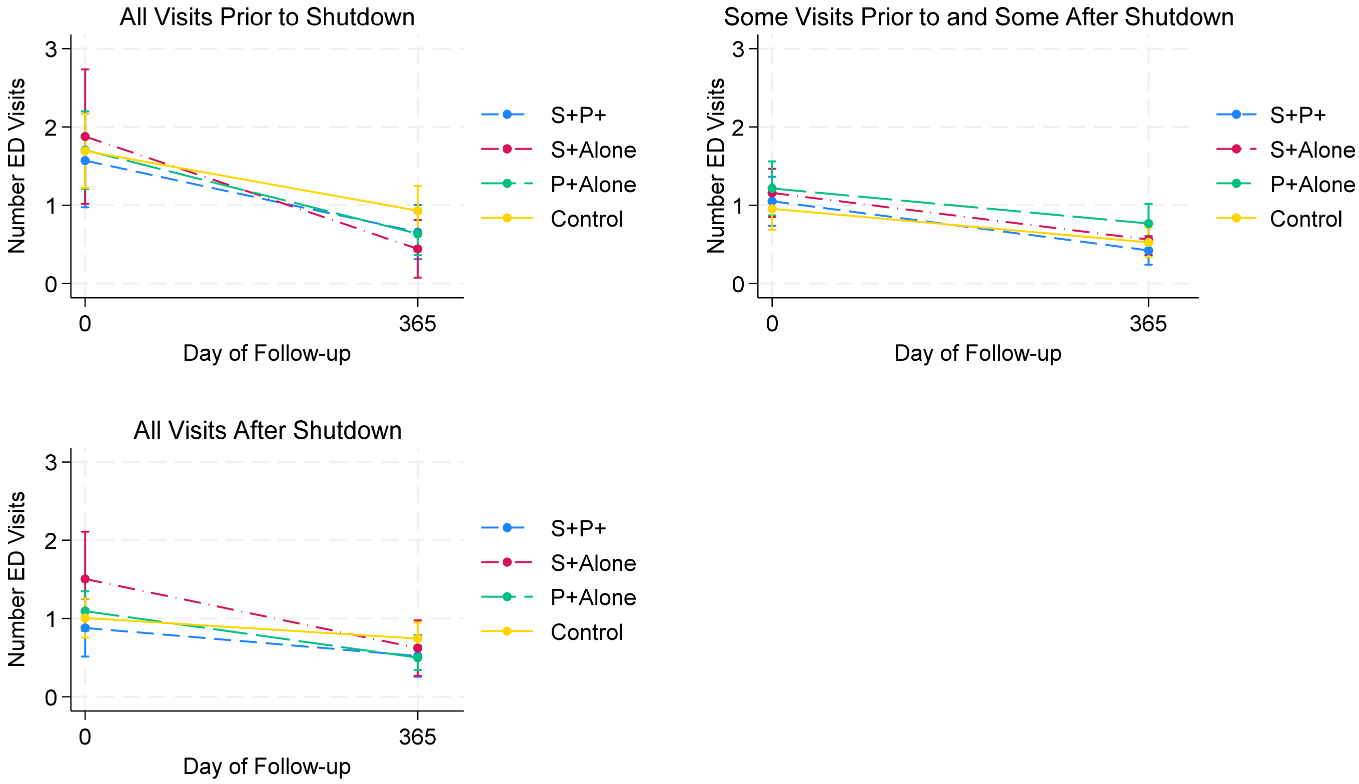

eFigure 12. Graphs of fitted Values of Number HOSP Visits in previous 365 days versus planned month of visit. Fitted values are from a mixed-effects binomial model (n=365) with random intercepts for child. (The model with random intercepts for both child and school did not converge.) The models included time (day of follow-up), indicator variables for each intervention, and time by intervention interaction terms. For Participants with all visits prior to shutdown: Number of participants in each treatment group = 23 for S+P+ (of which nboth = 23, n0only = 0, n12only = 0); 14 for S+Alone (of which nboth = 14, n0only = 0, n12only = 0); 37 for P+Alone (of which nboth = 37, n0only = 0, n12only = 0); 40 for Control (of which nboth = 40, n0only = 0, n12only = 0). For Participants with some visits prior to shutdown and some visits after shutdown: Number of participants in each treatment group = 64 for S+P+ (of which nboth = 63, n0only = 1, n12only = 0); 74 for S+Alone (of which nboth = 74, n0only = 0, n12only = 0); 62 for P+Alone (of which nboth = 62, n0only = 0, n12only = 0); 75 for Control (of which nboth = 72, n0only = 3, n12only = 0). For Participants with all visits after shutdown: Number of participants in each treatment group = 33 for S+P+ (of which nboth = 33, n0only = 0, n12only = 0); 24 for S+Alone (of which nboth = 24, n0only = 0, n12only = 0); 90 for P+Alone (of which nboth = 90, n0only = 0, n12only = 0); 86 for Control (of which nboth = 86, n0only = 0, n12only = 0).
The number of HOSP visits in the year prior is from EHR data and was measured at baseline and at day 365 (Month 12) on all participants, including those participants who did not have other outcomes measured at 12 months.

© 2024 Bryant-Stephens T et al. *JAMA Pediatrics*.

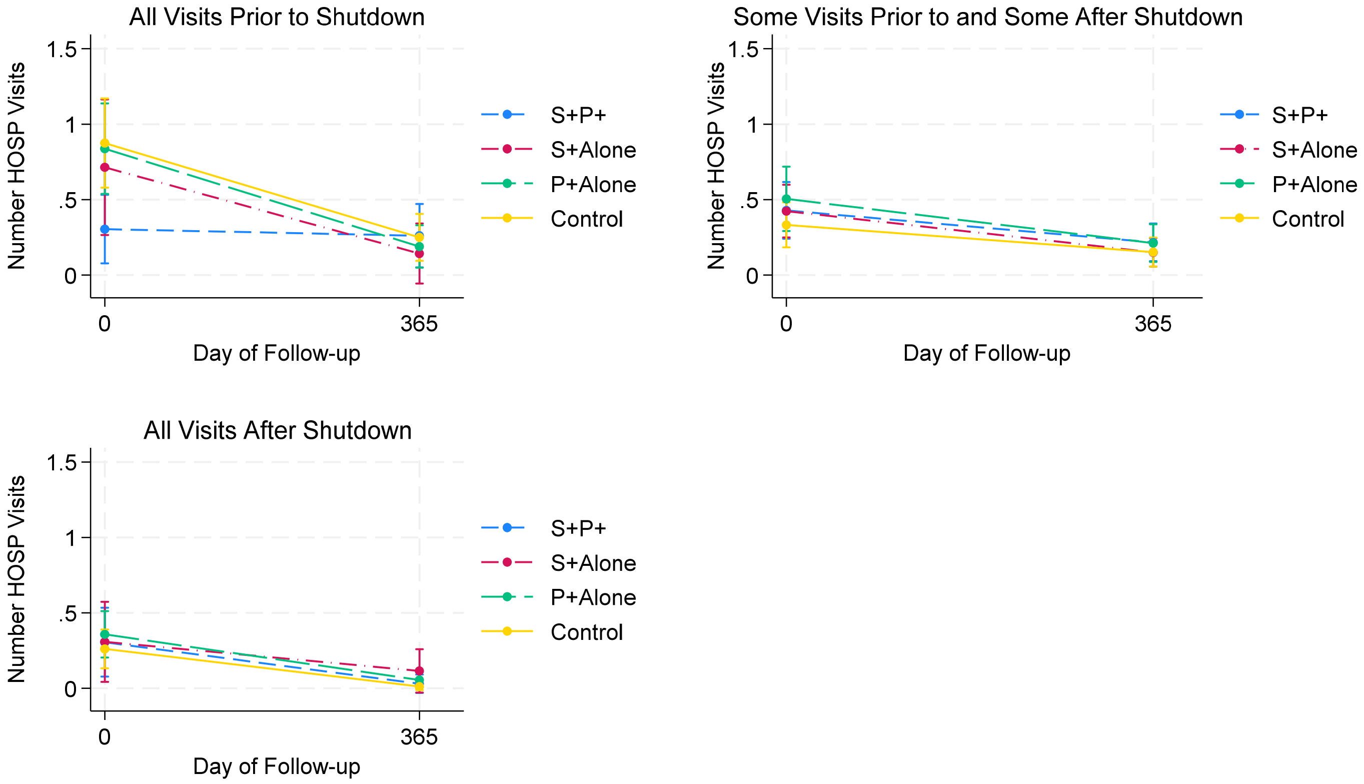

eFigure 13. Graphs of fitted Values of Asthma Control versus planned month of visit. Fitted values are from a linear GEEmixed-effects model with random intercepts for school and child. The models included time (day of follow-up), indicator variables for each intervention, and time by intervention interaction terms. For Participants with all visits prior to shutdown: Number of participants in each treatment group = 23 for S+P+ (of which nboth = 23, n0only = 0, n12only = 0); 14 for S+Alone (of which nboth = 14, n0only = 0, n12only = 0); 37 for P+Alone (of which nboth = 37, n0only = 0, n12only = 0); 40 for Control (of which nboth = 40, n0only = 0, n12only = 0). For Participants with some visits prior to shutdown and some visits after shutdown: Number of participants in each treatment group = 64 for S+P+ (of which nboth = 61, n0only = 3, n12only = 0); 74 for S+Alone (of which nboth = 70, n0only = 4, n12only = 0); 62 for P+Alone (of which nboth = 58, n0only = 4, n12only = 0); 74 for Control (of which nboth = 70, n0only = 4, n12only = 0). For Participants with all visits after shutdown: Number of participants in each treatment group = 33 for S+P+ (of which nboth = 32, n0only = 1, n12only = 0); 24 for S+Alone (of which nboth = 24, n0only = 0, n12only = 0); 89 for P+Alone (of which nboth = 87, n0only = 2, n12only = 0); 86 for Control (of which nboth = 83, n0only = 2, n12only = 1).
This analysis excluded 1 measurement taken after 800 days of follow-up.

© 2024 Bryant-Stephens T et al. *JAMA Pediatrics*.

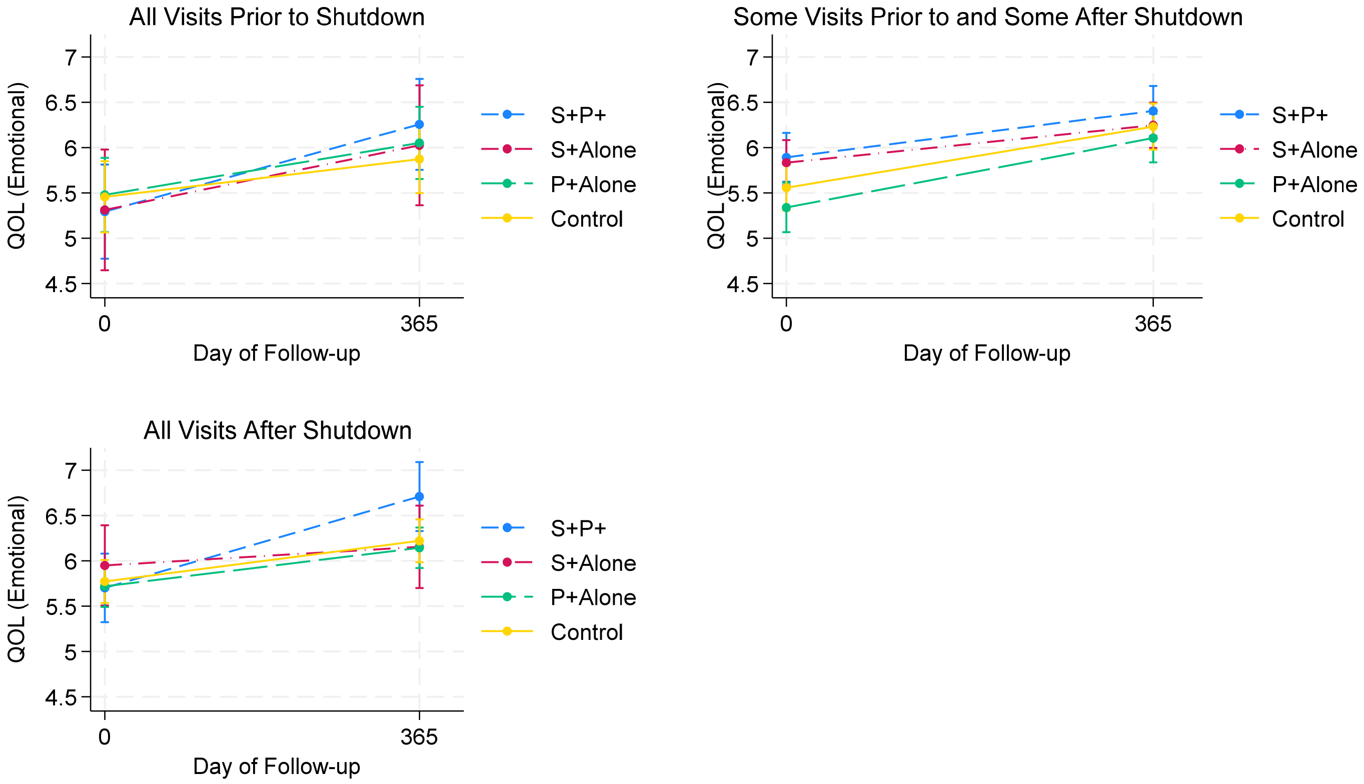

eFigure 14. Graphs of fitted Values of Quality of Life (Activity) versus planned month of visit. Fitted values are from a linear mixed-effects model with random intercepts for school and child. The models included time (day of follow-up), indicator variables for each intervention, and time by intervention interaction terms. For Participants with all visits prior to shutdown: Number of participants in each treatment group = 23 for S+P+ (of which nboth = 23, n0only = 0, n12only = 0); 14 for S+Alone (of which nboth = 14, n0only = 0, n12only = 0); 37 for P+Alone (of which nboth = 37, n0only = 0, n12only = 0); 40 for Control (of which nboth = 40, n0only = 0, n12only = 0). For Participants with some visits prior to shutdown and some visits after shutdown: Number of participants in each treatment group = 64 for S+P+ (of which nboth = 61, n0only = 3, n12only = 0); 74 for S+Alone (of which nboth = 70, n0only = 4, n12only = 0); 62 for P+Alone (of which nboth = 58, n0only = 4, n12only = 0); 74 for Control (of which nboth = 70, n0only = 4, n12only = 0). For Participants with all visits after shutdown: Number of participants in each treatment group = 33 for S+P+ (of which nboth = 32, n0only = 1, n12only = 0); 24 for S+Alone (of which nboth = 24, n0only = 0, n12only = 0); 89 for P+Alone (of which nboth = 86, n0only = 3, n12only = 0); 86 for Control (of which nboth = 83, n0only = 2, n12only = 1). This analysis excluded 1 measurement taken after 800 days of follow-up.

© 2024 Bryant-Stephens T et al. *JAMA Pediatrics*.

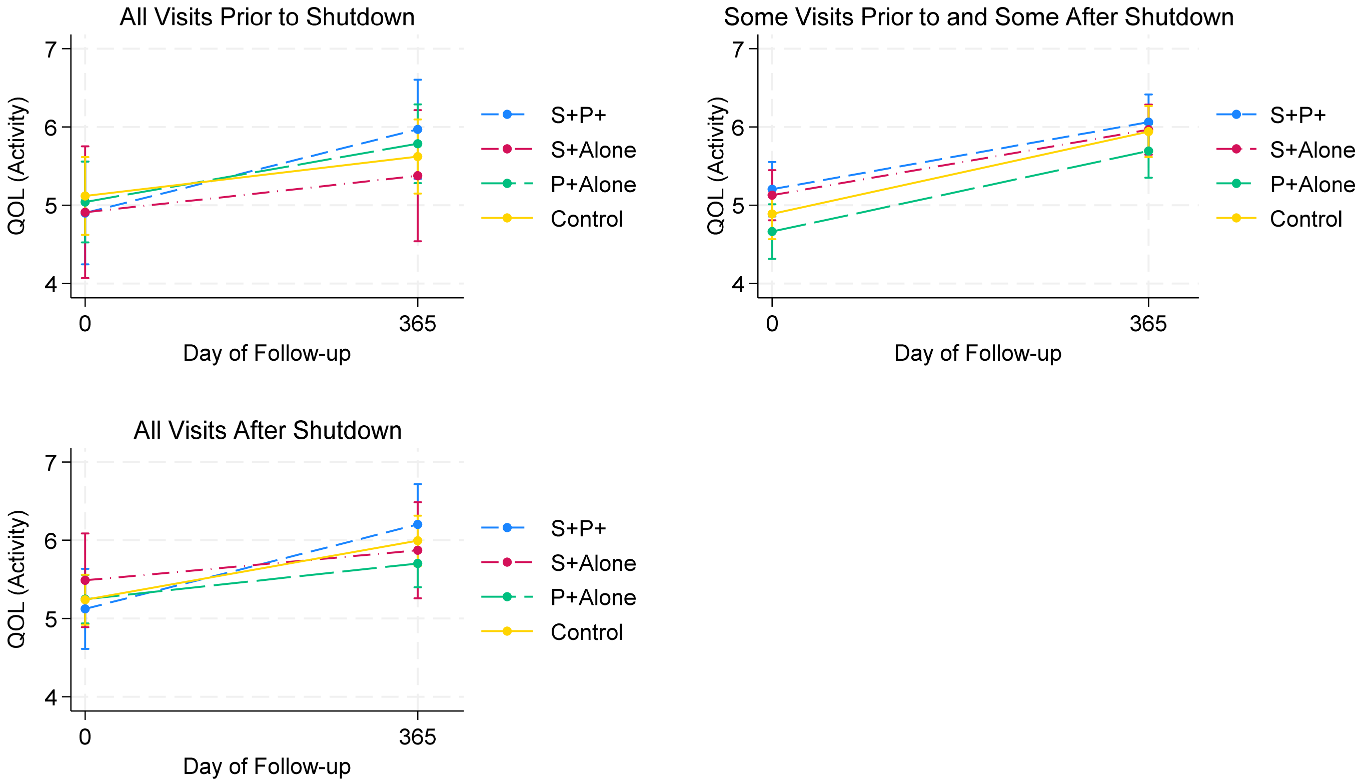

eFigure 15. Graphs of fitted Values of Number Steroid Courses in previous 365 days versus planned month of visit. Fitted values are from a mixed-effects Poisson model with random intercepts for child. The models included time (day of follow-up), indicator variables for each intervention, and time by intervention interaction terms. For Participants with all visits prior to shutdown: Number of participants in each treatment group = 23 for S+P+ (of which nboth = 23, n0only = 0, n12only = 0); 14 for S+Alone (of which nboth = 14, n0only = 0, n12only = 0); 37 for P+Alone (of which nboth = 37, n0only = 0, n12only = 0); 40 for Control (of which nboth = 40, n0only = 0, n12only = 0). For Participants with some visits prior to shutdown and some visits after shutdown: Number of participants in each treatment group = 64 for S+P+ (of which nboth = 63, n0only = 1, n12only = 0); 74 for S+Alone (of which nboth = 74, n0only = 0, n12only = 0); 62 for P+Alone (of which nboth = 62, n0only = 0, n12only = 0); 75 for Control (of which nboth = 72, n0only = 3, n12only = 0). For Participants with all visits after shutdown: Number of participants in each treatment group = 33 for S+P+ (of which nboth = 33, n0only = 0, n12only = 0); 24 for S+Alone (of which nboth = 24, n0only = 0, n12only = 0); 90 for P+Alone (of which nboth = 90, n0only = 0, n12only = 0); 86 for Control (of which nboth = 86, n0only = 0, n12only = 0). The number of Steroid visits in the year was available at baseline and at day 365 (Month 12) on all participants, including those participants who did not have other outcomes measured at 12 months.

© 2024 Bryant-Stephens T et al. *JAMA Pediatrics*.

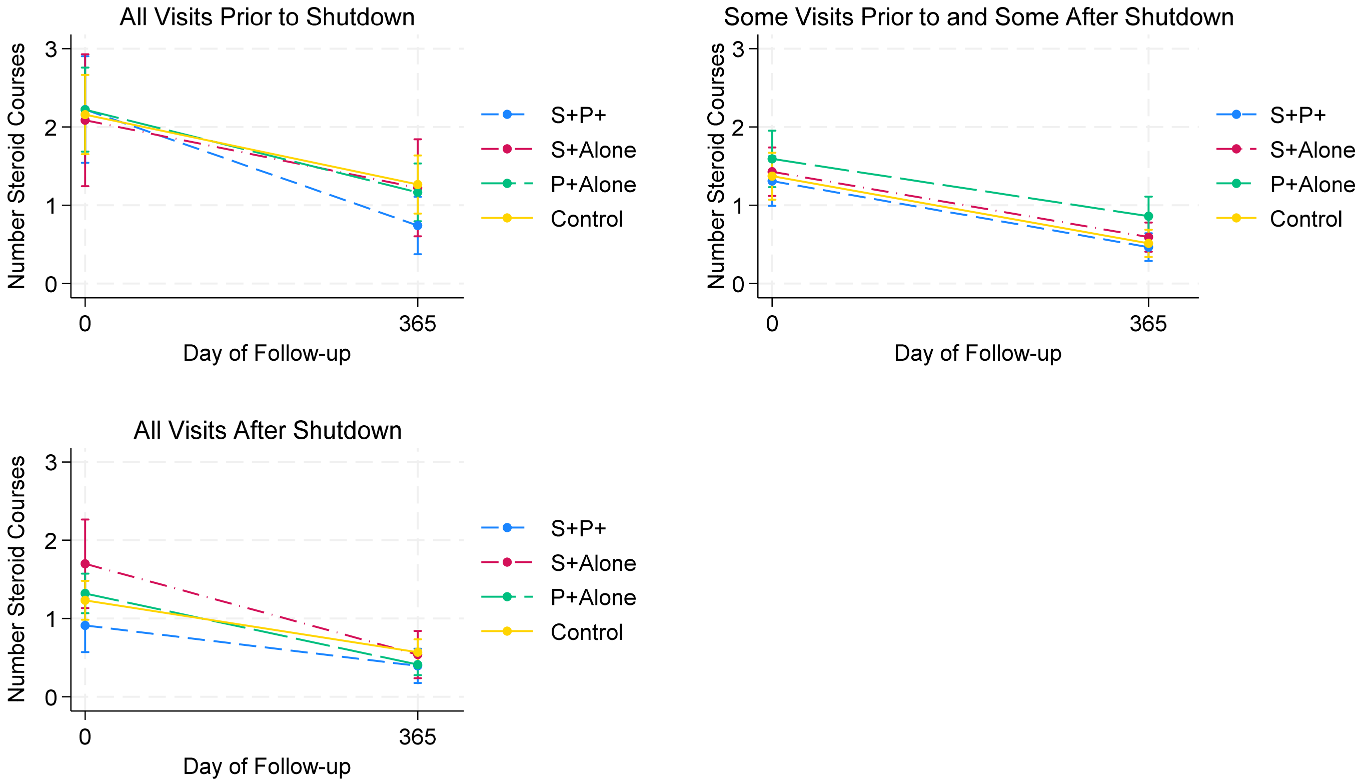

Section Three. Tables.

eTable 1. Fitted Values for Secondary Outcomes at Baseline and 12 Months. Results are provided for all participants (No Adjustment for COVID Pandemic Phase results below).

**Quality of Life – Emotional Functioning Domain**					
	P+ (N=308)	P- (N=312)			
Baseline	5.62	5.69			
Month Twelve	6.25	6.17			
Month Twelve minus Baseline	.63	.48	P+ minus P- difference = .15 (95% CI = -.05 to .34; p = .143)		
	S+ (N=232)	S- (N=388)			
Baseline	5.76	5.59			
Month Twelve	6.33	6.13			
Month Twelve minus Baseline	.58	.55	S+ minus S- difference = .03 (95% CI = -.17 to .23; p = .777)		
	S+P+ (N=120) to S+ Alone (N = 112)	S+P+ (N = 120) to P+ Alone (N = 188)	S+ Alone (N=112) to	P+ Alone (N=188) to	S+P+ Alone (N=120) to

© 2024 Bryant-Stephens T et al. *JAMA Pediatrics*.

			Control (N=200)	Control (N=200)	Control (N=200)
Baseline	5.73 vs 5.79	5.73 vs 5.54	5.79 vs 5.63	5.54 vs 5.63	5.73 vs 5.63
Month 12	6.46 vs 6.2	6.46 vs 6.11	6.2 vs 6.15	6.11 vs 6.15	6.46 vs 6.15
Month 12 minus Baseline	.73 vs .41	.73 vs .57	.41 vs .52	.57 vs .52	.73 vs .52
Difference in (Month 12 minus Baseline)	Diff = .32 (95% CI = 0 to .65; p = .052)	Diff = .16 (95% CI = -.12 to .45; p = .263)	Diff = -.11 (95% CI = -.4 to .18; p = .455)	Diff = .05 (95% CI = -.2 to .29; p = .697)	Diff = .21 (95% CI = -.08 to .5; p = .15)
**Quality of Life – Activity Limitations Domain**					
	P+ (N=308)	P- (N=312)			
Baseline	5.06	5.12			
Month Twelve	5.86	5.89			
Month Twelve minus Baseline	.8	.77	P+ minus P- difference = .03 (95% CI = -.24 to .29; p = .837)		
	S+ (N=232)	S- (N=388)			
Baseline	5.15	5.05			
Month Twelve	5.98	5.81			
Month Twelve minus Baseline	.83	.75	S+ minus S- difference = .08 (95% CI = -.2 to .36; p = .569)		
	S+P+ (N=120) to S+ Alone (N = 112)	S+P+ (N = 120) to P+ Alone (N = 188)	S+ Alone (N=112) to Control (N=200)	P+ Alone (N=188) to Control (N=200)	S+P+ Alone (N=120) to Control (N=200)
Baseline	5.12 vs 5.17	5.12 vs 5.01	5.17 vs 5.09	5.01 vs 5.09	5.12 vs 5.09
Month 12	6.08 vs 5.88	6.08 vs 5.72	5.88 vs 5.89	5.72 vs 5.89	6.08 vs 5.89
Month 12 minus Baseline	.96 vs .71	.96 vs .71	.71 vs .81	.71 vs .81	.96 vs .81
Difference in (Month 12 minus Baseline)	Diff = .25 (95% CI = -.19 to .69; p = .263)	Diff = .25 (95% CI = -.14 to .63; p = .205)	Diff = -.1 (95% CI = -.49 to .3; p = .622)	Diff = -.1 (95% CI = -.43 to .24; p = .567)	Diff = .15 (95% CI = -.24 to .54; p = .444)
**Total Courses of Oral Corticosteroids**					
	P+ (N=313)	P- (N=313)			
Baseline	1.52	1.50			
Month Twelve	.63	.68			
Month Twelve minus Baseline	-.89	-.82	P+ minus P- difference = -.07 (95% CI = -.3 to .17; p = .587)		
	S+ (N=233)	S- (N=393)			
Baseline	1.46	1.54			
Month Twelve	.57	.70			
Month Twelve minus Baseline	-.89	-.84	S+ minus S- difference = -.05 (95% CI = -.31 to .19; p = .674)		

© 2024 Bryant-Stephens T et al. *JAMA Pediatrics*.

	S+P+ (N=121) to S+ Alone (N = 112)	S+P+ (N = 121) to P+ Alone (N = 192)	S+ Alone (N=112) to Control (N=201)	P+ Alone (N=192) to Control (N=201)	S+P+ Alone (N=121) to Control (N=201)
Baseline	1.36 vs 1.57	1.36 vs 1.61	1.57 vs 1.46	1.61 vs 1.46	1.36 vs 1.46
Month 12	.49 vs .66	.49 vs .71	.66 vs .69	.71 vs .69	.49 vs .69
Month 12 minus Baseline	-.87 vs -.91	-.87 vs -.90	-.91 vs -.78	-.90 vs -.78	-.87 vs -.78
Difference in (Month 12 minus Baseline)	Diff = .04 (95% CI = -.35 to .42; p = .853)	Diff = -.03 (95% CI = -.31 to .37; p = .867)	Diff = -.13 (95% CI = -.49 to .22; p = .463)	Diff = -.13 (95% CI = -.43 to .18; p = .42)	Diff = -.1 (95% CI = -.43 to .23; p = .564)

*p<0.05

****Longitudinal Models for the Primary and Secondary Outcomes**:

QOL Emotional, measured at 0 and 12 months: Mixed-effects linear model with random intercepts for school and child. The models included time, indicator variables for each intervention, and time by intervention interaction terms. Time was modeled as day of follow-up.

QOL Activity, measured at 0 and 12 months: Mixed-effects linear model with random intercepts for school and child. The models included time, indicator variables for each intervention, and time by intervention interaction terms. Time was modeled as day of follow-up.

Systemic Steroid Courses: Mixed-effects generalized Poisson model with random intercept for each school and child. The models included time (day = 0 or 365), indicator variables for each intervention, and time by intervention interaction terms.

eTable 2. Fitted Values of Secondary Outcomes at 12 Months, Stratified by Study Completion Prior to COVID Pandemic Phase.

Daytime Symptoms					
	P+ (N=59)	P- (N=53)			
Baseline	3.46	2.42			
Month Twelve	1.19	1.95			
Month Twelve minus Baseline	-2.28	-.47	P+ minus P- difference = -1.81 (95% CI = -2.57 to -1.04; p < 0.0005)		
	S+ (N=36)	S- (N=76)			
Baseline	2.88	3.05			
Month Twelve	.92	1.85			
Month Twelve minus Baseline	-1.96	-1.2	S+ minus S- difference = -.76 (95% CI = -1.64 to .12; p = .091)		
	S+P+ (N=23) to S+ Alone (N = 13)	S+P+ (N = 23) to P+ Alone (N = 36)	S+ Alone (N=13) to Control (N=40)	P+ Alone (N =36) to Control (N=40)	S+P+ Alone (N=23) to Control (N=40)

© 2024 Bryant-Stephens T et al. *JAMA Pediatrics*.

Baseline	3.25 vs 1.78	3.25 vs 3.57	1.78 vs 2.61	3.57 vs 2.61	3.25 vs 2.61
Month 12	.69 vs 1.52	.69 vs 1.51	1.52 vs 2.09	1.51 vs 2.09	.69 vs 2.09
Month 12 minus Baseline	-2.56 vs -.26	-2.56 vs -2.06	-.26 vs -.52	-2.06 vs -.52	-2.56 vs -.52
Difference in (Month 12 minus Baseline)	Diff = -2.31 (95% CI = -3.79 to -.82; p = .002)	Diff = -.5 (95% CI = -1.8 to .79; p = .446)	Diff = .26 (95% CI = -.9 to 1.43; p = .658)	Diff = -1.54 (95% CI = -2.45 to -.62; p = .001)	Diff = -2.04 (95% CI = -3.22 to -.86; p = .001)
Nighttime Symptoms					
	P+ (N=60)	P- (N=53)			
Baseline	2.04	1.95			
Month Twelve	.86	1.56			
Month Twelve minus Baseline	-1.18	-.38	P+ minus P- difference = -.8 (95% CI = -1.42 to -.19; p = .011)		
	S+ (N=37)	S- (N=76)			
Baseline	2.23	1.89			
Month Twelve	1.06	1.26			
Month Twelve minus Baseline	-1.18	-.63	S+ minus S- difference = -.55 (95% CI = -1.25 to .15; p = .126)		
	S+P+ (N=23) to S+ Alone (N = 14)	S+P+ (N = 23) to P+ Alone (N = 37)	S+ Alone (N=14) to Control (N=39)	P+ Alone (N =37) to Control (N=39)	S+P+ Alone (N=23) to Control (N=39)
Baseline	2.4 vs 1.96	2.4 vs 1.86	1.96 vs 1.96	1.86 vs 1.96	2.4 vs 1.96
Month 12	.65 vs 1.75	.65 vs .99	1.75 vs 1.53	.99 vs 1.53	.65 vs 1.53
Month 12 minus Baseline	-1.76 vs -.21	-1.76 vs -.86	-.21 vs -.43	-.86 vs -.43	-1.76 vs -.43
Difference in (Month 12 minus Baseline)	Diff = -1.55 (95% CI = -2.81 to -.29; p = .016)	Diff = -.9 (95% CI = -2 to .21; p = .111)	Diff = .22 (95% CI = -.70 to 1.15; p = .637)	Diff = -.43 (95% CI = -1.12 to .26; p = .22)	Diff = -1.33 (95% CI = -2.39 to -.26; p = .015)
Number ED Visits					
	P+ (N=60)	P- (N=54)			

© 2024 Bryant-Stephens T et al. *JAMA Pediatrics*.

Baseline	1.66	1.72			
Month Twelve	.6	.79			
Month Twelve minus Baseline	-1.06	-.94	P+ minus P- difference = -.12 (95% CI = -.72 to .47; p = .687)		
	S+ (N=37)	S- (N=77)			
Baseline	1.69	1.69			
Month Twelve	.55	.76			
Month Twelve minus Baseline	-1.15	-.93	S+ minus S- difference = -.22 (95% CI = -.85 to .41; p = .499)		
	S+P+ (N=23) to S+ Alone (N = 14)	S+P+ (N = 23) to P+ Alone (N = 37)	S+ Alone (N=14) to Control (N=40)	P+ Alone (N =37) to Control (N=40)	S+P+ Alone (N=23) to Control (N=40)
Baseline	1.58 vs 1.88	1.58 vs 1.71	1.88 vs 1.67	1.71 vs 1.67	1.58 vs 1.67
Month 12	.61 vs .43	.61 vs .6	.43 vs .91	.6 vs .91	.61 vs .91
Month 12 minus Baseline	-.96 vs -1.45	-.96 vs -1.12	-1.45 vs -.76	-1.12 vs -.76	-.96 vs -.76
Difference in (Month 12 minus Baseline)	Diff = .48 (95% CI = -.6 to 1.56; p = .383)	Diff = .15 (95% CI = -.67 to .97; p = .718)	Diff = -.68 (95% CI = -1.7 to .33; p = .186)	Diff = -.35 (95% CI = -1.08 to .37; p = .34)	Diff = -.2 (95% CI = -1.01 to .61; p = .626)
Hospital Visits					
	P+ (N=60)	P- (N=54)			
Baseline	.63	.83			
Month Twelve	.22	.22			
Month Twelve minus Baseline	-.42	-.61	P+ minus P- difference = .19 (95% CI = -.17 to .56; p = .294)		
	S+ (N=37)	S- (N=77)			
Baseline	.46	.86			
Month Twelve	.22	.22			
Month Twelve minus Baseline	-.24	-.64	S+ minus S- difference = .39 (95% CI = .04 to .75; p = .029)		

© 2024 Bryant-Stephens T et al. *JAMA Pediatrics*.

	S+P+ (N=23) to S+ Alone (N = 14)	S+P+ (N = 23) to P+ Alone (N = 37)	S+ Alone (N=14) to Control (N=40)	P+ Alone (N =37) to Control (N=40)	S+P+ Alone (N=23) to Control (N=40)
Baseline	.3 vs .71	.3 vs .84	.71 vs .88	.84 vs .88	.3 vs .88
Month 12	.26 vs .14	.26 vs .19	.14 vs .25	.19 vs .25	.26 vs .25
Month 12 minus Baseline	-.04 vs -.57	-.04 vs -.65	-.57 vs -.63	-.65 vs -.63	-.04 vs -.63
Difference in (Month 12 minus Baseline)	Diff = .53 (95% CI = -.05 to 1.11; p = .073)	Diff = .61 (95% CI = .15 to 1.06; p = .008)	Diff = .05 (95% CI = -.54 to .64; p = .859)	Diff = -.02 (95% CI = -.49 to .44; p = .921)	Diff = .58 (95% CI = .13 to 1.03; p = .012)
QOL Emotional					
	P+ (N=60)	P- (N=54)			
Baseline	5.41	5.42			
Month Twelve	6.13	5.91			
Month Twelve minus Baseline	.72	.49	P+ minus P- difference = .24 (95% CI = -.26 to .74; p = .347)		
	S+ (N=37)	S- (N=77)			
Baseline	5.3	5.47			
Month Twelve	6.17	5.96			
Month Twelve minus Baseline	.88	.49	S+ minus S- difference = .39 (95% CI = -.15 to .92; p = .157)		
	S+P+ (N=23) to S+ Alone (N = 14)	S+P+ (N = 23) to P+ Alone (N = 37)	S+ Alone (N=14) to Control (N=40)	P+ Alone (N =37) to Control (N=40)	S+P+ Alone (N=23) to Control (N=40)
Baseline	5.29 vs 5.31	5.29 vs 5.48	5.31 vs 5.46	5.48 vs 5.46	5.29 vs 5.46
Month 12	6.26 vs 6.03	6.26 vs 6.05	6.03 vs 5.87	6.05 vs 5.87	6.26 vs 5.87
Month 12 minus Baseline	.96 vs .71	.96 vs .57	.71 vs .42	.57 vs .42	.96 vs .42
Difference in (Month 12 minus Baseline)	Diff = .25 (95% CI = -.68 to 1.17; p = .598)	Diff = .39 (95% CI = -.32 to 1.09; p = .279)	Diff = .3 (95% CI = -.55 to 1.14; p = .493)	Diff = .16 (95% CI = -.44 to .75; p = .61)	Diff = .55 (95% CI = -.14 to 1.23; p = .119)
QOL Activity					

© 2024 Bryant-Stephens T et al. *JAMA Pediatrics*.

	P+ (N=60)	P- (N=54)			
Baseline	4.99	5.06			
Month Twelve	5.86	5.56			
Month Twelve minus Baseline	.87	.5	P+ minus P- difference = .37 (95% CI = -.28 to 1.02; p = .261)		
	S+ (N=37)	S- (N=77)			
Baseline	4.9	5.08			
Month Twelve	5.76	5.7			
Month Twelve minus Baseline	.86	.61	S+ minus S- difference = .24 (95% CI = -.46 to .95; p = .495)		
	S+P+ (N=23) to S+ Alone (N = 14)	S+P+ (N = 23) to P+ Alone (N = 37)	S+ Alone (N=14) to Control (N=40)	P+ Alone (N =37) to Control (N=40)	S+P+ Alone (N=23) to Control (N=40)
Baseline	4.9 vs 4.91	4.9 vs 5.04	4.91 vs 5.12	5.04 vs 5.12	4.9 vs 5.12
Month 12	5.97 vs 5.38	5.97 vs 5.79	5.38 vs 5.62	5.79 vs 5.62	5.97 vs 5.62
Month 12 minus Baseline	1.07 vs .47	1.07 vs .74	.47 vs .5	.74 vs .5	1.07 vs .5
Difference in (Month 12 minus Baseline)	Diff = .6 (95% CI = -.61 to 1.81; p = .33)	Diff = .32 (95% CI = -.6 to 1.25; p = .49)	Diff = -.04 (95% CI = -1.14 to 1.07; p = .949)	Diff = .24 (95% CI = -.54 to 1.02; p = .547)	Diff = .57 (95% CI = -.33 to 1.46; p = .216)
Total Courses of Oral Corticosteroids					
	P+ (N=60)	P- (N=54)			
Baseline	2.22	2.14			
Month Twelve	1	1.25			
Month Twelve minus Baseline	-1.22	-.89	P+ minus P- difference = -.33 (95% CI = -1.02 to .35; p = .336)		
	S+ (N=37)	S- (N=77)			
Baseline	2.17	2.19			
Month Twelve	.92	1.22			
Month Twelve minus Baseline	-1.25	-.97	S+ minus S- difference = -.28 (95% CI = -1 to .44; p = .449)		

© 2024 Bryant-Stephens T et al. *JAMA Pediatrics*.

	S+P+ (N=23) to S+ Alone (N = 14)	S+P+ (N = 23) to P+ Alone (N = 37)	S+ Alone (N=14) to Control (N=40)	P+ Alone (N =37) to Control (N=40)	S+P+ Alone (N=23) to Control (N=40)
Baseline	2.22 vs 2.09	2.22 vs 2.22	2.09 vs 2.16	2.22 vs 2.16	2.22 vs 2.16
Month 12	.74 vs 1.22	.74 vs 1.17	1.22 vs 1.27	1.17 vs 1.27	.74 vs 1.27
Month 12 minus Baseline	-1.48 vs -.86	-1.48 vs -1.06	-.86 vs -.89	-1.06 vs -.89	-1.48 vs -.89
Difference in (Month 12 minus Baseline)	Diff = -.62 (95% CI = -1.83 to .6; p = .318)	Diff = -.43 (95% CI = -1.38 to .52; p = .38)	Diff = .03 (95% CI = -1.1 to 1.16; p = .959)	Diff = -.16 (95% CI = -1 to .67; p = .702)	Diff = -.59 (95% CI = -1.52 to .34; p = .216)

*p<0.05

****Longitudinal Models for the Primary and Secondary Outcomes**:

Daytime Symptoms in past 2 weeks, at 0 and 12 months: Mixed-effects generalized binomial model (n=14) with random intercepts for child. (The model with random intercepts for both child and school did not converge.) The models included time (day of follow-up), indicator variables for each intervention, and time by intervention interaction terms.

Nighttime Symptoms in past 2 weeks, at 0 and 12 months: Mixed-effects generalized binomial model (n=14) with random intercepts for child. (The model with random intercepts for both child and school did not converge.) The models included time (day of follow-up), indicator variables for each intervention, and time by intervention interaction terms.

Number ED Visits One Year Prior, at 0 and 12 months: Mixed-effects negative binomial model (n=365) with random intercepts for child. (The model with random intercepts for both child and school did not converge.) The models included time (day =0 or 365), indicator variables for each intervention, and time by intervention interaction terms.

Number Hospitalizations One Year Prior, at 0 and 12 months: Mixed-effects generalized binomial model (n=365) with random intercepts for child. (The model with random intercepts for both child and school did not converge.) The models included time (day =0 or 365), indicator variables for each intervention, and time by intervention interaction terms.

QOL Emotional, measured at 0 and 12 months: Mixed-effects linear model with random intercepts for school and child. The models included time, indicator variables for each intervention, and time by intervention interaction terms. Time was modeled as day of follow-up.

QOL Activity, measured at 0 and 12 months: Mixed-effects linear model with random intercepts for school and child. The models included time, indicator variables for each intervention, and time by intervention interaction terms. Time was modeled as day of follow-up.

Systemic Steroid Courses: Mixed-effects generalized Poisson model with random intercept for each school and child. The models included time (day = 0 or 365), indicator variables for each intervention, and time by intervention interaction terms.

© 2024 Bryant-Stephens T et al. *JAMA Pediatrics*.
