# Supplementary material for: Community Health Workers Linking Clinics and Schools and Asthma Control: A Randomized Clinical Trial
Source: JAMA Pediatr. 2024 Oct 21;178(12):1260–9. doi: 10.1001/jamapediatrics.2024.3967 (PMC11581744; doi:10.1001/jamapediatrics.2024.3967)
Supplement: Supplement 4. — Data Sharing Statement. [file jamapediatr-e243967-s004.pdf]

Data Sharing Statement

Bryant-Stephens. Community Health Workers Linking Clinics and Schools and Asthma Control.
JAMA Pediatr. Published October 21, 2024. doi:10.1001/jamapediatrics.2024.3967

Data
Data available: No
